# Smurf2 knockdown attenuates the progression of diabetic nephropathy by inhibiting mesangial cell proliferation and fibrosis through suppressing EYA2 ubiquitination

**DOI:** 10.1080/0886022X.2025.2520904

**Published:** 2025-06-24

**Authors:** Lizhen Chen, Yuqing Chen, Fei Liu, Yinhao Liu

**Affiliations:** a Department of Diabetes Nephrology, Zhangzhou Hospital of Traditional Chinese Medicine, Zhangzhou, China; b Department of Pharmacy, Zhangzhou Health Vocational College, Zhangzhou, China

**Keywords:** Diabetic nephropathy, mesangial cell, ubiquitination, Smurf2, EYA2

## Abstract

Diabetic nephropathy (DN) is a serious microvascular complication of diabetes and the main cause of end-stage renal disease. Smurf2 is a member of ubiquitin ligases. In this study, we aimed to investigate the mechanism by which Smurf2 mediated the development of DN. C57BL/6 mice were intraperitoneally injected into streptozotocin to construct a DN mouse model, and mouse mesangial cells (MCs) were treated with high glucose (HG) to establish a cell model. A cell counting kit 8, EDU staining and Western blot assay were performed to assess cell proliferation and fibrosis. The interaction between Smurf2 and EYA2 was identified by immunoprecipitation, and the ubiquitination of EYA2 was detected by Western blot. In addition, the kidney injury of DN mice was evaluated by hematoxylin-eosin and Masson staining and the detection of biochemical parameters. Results suggested that Smurf2 expression was increased in DN mouse model and HG-treated MCs; Smurf2 knockdown inhibited cell proliferation and fibrosis in HG-treated MCs. Mechanically, Smurf2 knockdown inhibited the ubiquitination on EYA2, leading to the suppression of EYA2 degradation and an upregulation in EYA2 protein levels. Moreover, EYA2 knockdown restored cell proliferation and fibrosis in HG-treated MCs inhibited by Smurf2 knockdown. Additionally, Smurf2 knockdown inhibited kidney injury and fibrosis in DN mouse model. In conclusion, we demonstrated that Smurf2 knockdown attenuated the progression of DN by inhibiting MCs proliferation and fibrosis through suppressing EYA2 ubiquitination, which may provide a novel insight into the pathogenesis of DN.

## Introduction

Diabetic nephropathy (DN) is one of the most common complications of diabetes and the leading cause of chronic kidney failure worldwide, which is a type of common microvascular complication, significantly increasing the mortality rate of patients with diabetes [1,2]. The treatments of DN include target-based pharmacotherapy, cell therapies, and dietary regulation, but the therapeutic effect is still limited [3]. The pathogenesis of DN is not clear yet, which may regulated by multiple factors. Therefore, elucidating the pathogenesis of DN is essential for the development of effective treatments.

DN affects all the cell types of the kidney, including glomerular podocytes, interstitial fibroblasts, tubular epithelia, and mesangial cells (MCs) [4–7]. Among them, irreversible fibrosis and hyperproliferation of MCs are the main pathological manifestations of DN [8,9]. MCs are necessary in maintaining glomerular structural integrity and regulating glomerular filtration [10]. A previous study reported that the abnormal proliferation of MCs exists in the early stage of DN [11]. Therefore, inhibition of MCs proliferation and fibrosis may alleviate the progression of DN.

Ubiquitination is a post-translational modification, which is mediated by three kinds of enzymes, ubiquitin**-**activating enzymes (E1), ubiquitin**-**conjugating enzymes (E2), and ubiquitin ligases (E3) [12]. Ubiquitination occurs by the combined action of E1, E2, and E3 to covalently attach a 76 amino acid ubiquitin protein to the target protein, thereby regulating protein stability and degradation and affecting intracellular signaling and metabolic regulation [13,14]. Several studies have demonstrated that ubiquitination regulates the stability of substrate proteins, thereby influencing the progression of DN through mechanisms such as the modulation of ferroptosis and mitochondrial dysfunction [15–17]. Smurf2 is an E3 ubiquitin ligase belonging to the subfamily of neural precursor cell-expressed developmentally down-regulated protein 4, it has been demonstrated to regulate the development of multiple diseases, such as cancers and pulmonary fibrosis [18,19]. Smurf2 initially identified as a regulator of Smad protein stability in the TGF-β/BMP signaling pathway [20]. Notably, TGF-β superfamily of signaling proteins plays an important role in the development and progression of diabetic kidney diseases [21]. Activated TGF-β can further stimulate podocytes exposed to hyperglycemia and inflammatory microenvironments into a pro-fibrotic state [22]. Therefore, we speculated that Smurf2 may also participate in the development of DN. Kim et al. [23] revealed the mechanism by which Smurf2 mediates fibrosis of MCs by regulating ChREBP ubiquitination. However, the regulation of ubiquitination on DN still needs to be further studied.

Eyes absent homolog (EYA) is a transcriptional co-activator with a phosphatase function, which controls cell proliferation and DNA damage repair in diseases [24]. EYA2 is a member of the EYA protein family, and it is characterized by a tissue**-**specific expression in different types of tumors. Current research on EYA2 has mostly focused on cancer and found that it is tissue-specifically expressed in different tumors and regulates tumor development [25,26]. However, whether EYA2 regulates the development of DN remains unclear.

In this study, we investigated the effects of EYA2 ubiquitination regulated by Smurf2 on MC proliferation and fibrosis. These results may provide some novel insights into the pathogenesis of DN.

## Methods

### Animal study

Animal study was approved by the Ethics Committee of MDKN Biotechnology Co., Lt (ethical number: MDKN-2023-187). A total of 24 C57BL/6 mice were housed at 21-**23 °C**, with a relative humidity of 60–70% and a 12-h light/dark cycle condition. After one week of adaptive feeding, mice were randomly divided into four groups (6 mice/group, based on our lab’s prior experience and previously published studies [27,28]): the control group, the DN group, the DN+LV-shNC group, and the DN+LV-shSmurf2 group. The day before establishment of DN mouse model, mice in the DN+LV-shNC group and the DN+LV-shSmurf2 group were injected with lentivirus carrying shNC or shSmurf2 plasmids through the tail vein, and other mice were injected with saline.

DN mouse model was established according to the method as previously reported [29]. The mice were fasted for 4 h before each injection. Mice were intraperitoneally injected with 50 mg/kg streptozotocin (STZ) at day 1 and day 3 (two injections in total). Mice in the control group were injected with same volume of citrate buffer in the same way. Blood samples were collected from the tail vein 10 days after the second injection to measure blood glucose levels. The successful establishment of the DN model was confirmed by measuring blood glucose levels (≥16.67 mmol/L), combined with histopathological and biochemical analysis. After detection of proteinuria, mice were anesthetized with 0.1% pentobarbital sodium for blood collection, and then were sacrificed by cervical dislocation for kidney collection.

### Cell culture and treatment

The mouse MCs (SV40 MES 13) provided by American Type Culture Collection (ATCC, Manassas, VA, USA) were cultured in Dulbecco’s modified eagle medium (DMEM, Gibco, Grand Island, NY, USA) supplement with 20% fetal bovine serum (FBS; Gibco). Cells were divided into two groups, normal glucose (NG) group and high glucose (HG) group. In the HG group, cells were treated with 25 mmol/L glucose for 24 h to simulate the growth conditions under DN.

### Cell transfection

Short hairpin RNA targeting Smurf2 (shSmurf2), shRNA targeting EYA2 (shEYA2) and shRNA negative control (shNC) were obtained from GenePharma (Shanghai, China). The plasmids were transfected into MCs using Lipofectamine 2000 reagent (11668030, Invitrogen, Carlsbad, CA, USA) according to the guidelines. The transfected cells were cultured for 48 h at 37 °C for subsequent experiments.

### Quantitative real-time PCR (qPCR)

Total RNA was extracted by Trizol reagent (15596026, Invitrogen) and reverse transcribed into cDNA using a RevertAid first strand cDNA synthesis kit (K1621, Thermo Scientific, Waltham, MA, USA). qPCR was carried out with SYBR green master mix (A46110, Thermo Scientific) on CFX96 system (Bio-Rad, Hercules, CA, USA). Relative mRNA expression was calculated using the 2^−ΔΔCt^ method as normalized to β-actin. The primers for qPCR are as follows: Smurf2, 5′-AAACAGTTGCTTGGGAAGTCA-3′ (sense) and 5′-TGCTCAACACAGAAGGTATGGT-3′ (antisense); EYA2, 5′-AGGCACCACCCTATACAGC-3′ (sense) and 5′-TGAAGCTCGGTCCATAGCTCA-3′ (antisense).

### Cell viability

Cell viability was measured using a cell counting kit-8 (CCK8; C0038, Beyotime, Shanghai, China). Briefly, cells (2 × 10^3^/well) were seeded in 96-well plates and cultured for 48 h. Then, each well was added with 10 μl CCK8 solution and incubated for 1 h. Afterwards, the absorbance was measured using a microplate reader at the wavelength of 450 nm.

### EDU staining

An EdU Apollo567 *in vitro* kit (CA1170, Solarbio, Beijing, China) was used to evaluate cell proliferation. Cells (1 × 10^5^/well) were seeded in 24-well plate and cultured for 48 h. Next, EDU solution (10 μM) was added to each well for 2 h of incubation, and then, cells were fixed with 4% polyformaldehyde (P0099, Beyotime) fixing solution for 30 min at room temperature. Afterwards, cells were stained with 100 µL 1 × Apollo for 30 min protected from light. The nucleus was stained by 4′,6-diamidino-2-phenylindole (DAPI; C1005, Beyotime) for 5 min and the EDU**-**positive cells were observed by a fluorescence microscope.

### Western blot assay

Total protein was isolated by radio immunoprecipitation assay (RIPA) lysis buffer (89901, Thermo Scientific) and quantified using a BCA kit (P0010, Beyotime). The protein samples were loaded on 10% SDS-polyacrylamide gel for electrophoresis and then transferred to PVDF membranes, blocked with 5% skimmed milk for 1 h, and incubated overnight at 4 °C with anti-fibronectin (1: 1000, ab268020, Abcam, Cambridge, UK), anti-collagen I (1: 1000, ab138492), anti-α-SMA (1: 1000, ab5694), anti-EYA2 (1: 2000, ab95875), anti-Smurf2 (1: 1000, ab53316), anti-ubiquitin (1: 1000, EP8589) and anti-β-actin (1: 5000, ab8227). Afterwards, membranes were washed with Tris**-**buffered saline-Tween (TBST) for three times and incubated with secondary antibody (1: 10000, ab6721, Abcam, Cambridge, UK) for 1 h. Blots were visualized using the ECL reagent (36223ES60, Yeasen, Shanghai, China).

### Immunoprecipitation (IP) assay

The interaction between Smurf2 and EYA2 was evaluated using a Pierce classic IP kit (26146, Thermo Scientific). Cells were lysed with the lysis buffer for 5 min, and the lysate was incubated overnight with anti-Flag or anti-HA at 4 °C. After incubation, the mixture was then incubated with protein A/G magnetic beads for 1 h. Beads were washed using the lysis buffer and collected, and the antigen-antibody mixture was eluted. The levels of proteins were measured using western blot assay.

### EYA2 ubiquitination detection

His-tagged ubiquitin (UB) wild type (WT), His-UB K48 or K63 mutation plasmids, HA-Smurf2, and Flag-EYA2 were co-transfected into MCs. After 48 h, the cell lysates were collected and IP with anti-Flag magnetic beads. Afterward, the ubiquitination was evaluated using western blot assay.

### Protein stability detection

To assess the protein stability of EYA2, MCs were treated with 10 μM cycloheximide (CHX, HY-12320, MedChemExpress, Monmouth Junction, NJ, USA) or 10 μM CHX + 10 μM MG132 (MKBio, Shanghai, China) for 0, 4, 8, 12, 16, 20, and 24 h. The protein expression was evaluated by western blot assay.

### Hematoxylin–eosin (HE) staining

The kidney tissues from different groups of mice were stained by an HE staining kit (C0105S, Beyotime). Kidney tissues were first fixed with 4% paraformaldehyde at **4 °C** for 24 h, then dehydrated, transparent, embedded with paraffin, and cut into 5 μm thick paraffin sections. After respectively dewaxing and dehydrating with xylene and ethanol, the sections were stained with hematoxylin for 10 min and eosin for 2 min. Finally, paraffin sections were sealed with resin and observed using an optical microscope.

### Masson staining

The pathological changes in mice kidneys were assessed using a Masson’s trichrome staining kit (C0189S, Beyotime). Paraffin sections after dewaxing and dehydrating were stained with 50 μl hematoxylin for 5 min, 50 μl ponceau-acid magenta for 10 min, and 50 μl brilliant green for 1 min, respectively. The sections were observed using an optical microscope.

### Blood glucose detection

The blood glucose of different groups of mice was measured using a glucose assay kit (S0201S, Beyotime). The blood of mice (5 μl) and glucose assay reagent (185 μl) were added into PCR tubes and mixed using a vortex mixer. The samples were heated at 95 °C for 8 min on a PCR apparatus and cooled to 4 °C. The absorbance was measured using a microplate reader at the wavelength of 630 nm.

### Proteinuria detection

The mice were placed in metabolic cages for urine collection to measure 24-h proteinuria. The proteinuria was measured using an urine protein content assay kit (BC5655, Solarbio). The experiment was performed according to the instructions. The absorbance was measured using a microplate reader at the wavelength of 560 nm.

### Serum creatinine detection

The serum creatinine was evaluated using a creatinine assay kit (D799853, Sangon, Shanghai, China). The serums (6 μl) were first incubated with 180 μl reagent 1 at 37 °C for 5 min and then incubated with 60 μl reagent 2 for 5 min. After each incubation, the absorbance was measured using a microplate reader at the wavelength of 546 nm.

### Blood urea nitrogen detection

An urea nitrogen assay kit (BC1530, Solarbio) was performed to measure blood urea nitrogen in different groups of mice. Briefly, the serums were mixed with 60 μl reagent 1 and 110 μl reagent 2 and incubated at 37 °C for 10 min. Then, the mixture was incubated with 120 μl reagent 3 and 90 μl reagent 4 at 37 °C for 30 min. Finally, the mixture was mixed with 90 μl distilled water and the absorbance was measured using a microplate reader at the wavelength of 630 nm.

### Relative kidney weight calculation

After blood collection, the mice were sacrificed, and both kidneys were removed and weighed. The ratio of kidney weight to mice body weight was relative kidney weight.

### Statistical analysis

The data were analyzed and processed by GraphPad Prism 7 software. Results were expressed as mean ± standard deviation of at least three replicates. Student’s t-test was used to compare the two groups, and one-way analysis of variance (ANOVA) was used to compare the multiple groups. *p* < 0.05 was considered as statistically significant.

## Results

### Smurf2 expression is increased in DN mouse model and HG-treated MCs

**Figure 1.**
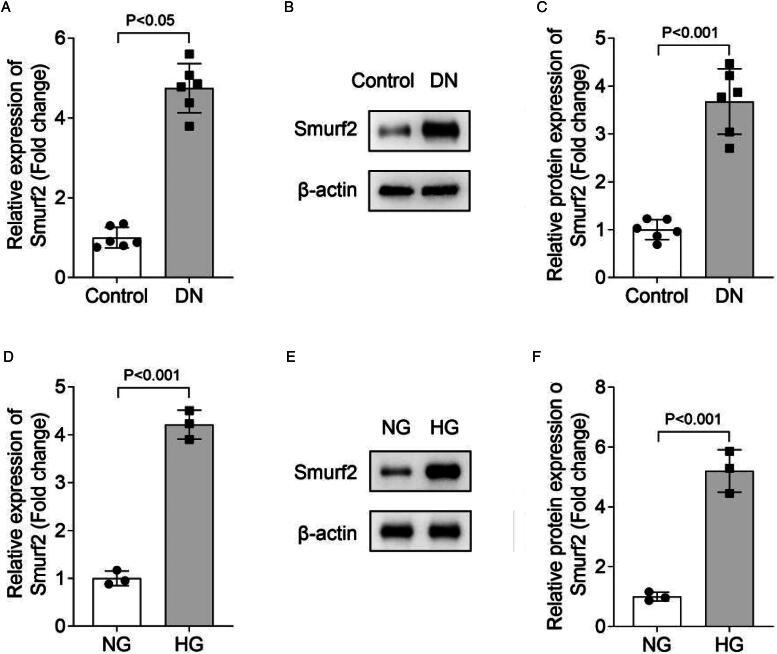
The expression of Smurf2 was increased in DN mice and HG-treated MCs. (A) Smurf2 mRNA expression in mice was measured using qPCR. (B and C) The protein levels of Smurf2 in mice were detected by Western blot. (D) The expression of Smurf2 in MCs was measured using qPCR. (E and F) The protein levels of Smurf2 in MCs were detected by Western blot.

Before the study, we first compared renal function and fibrosis between mice in DN group and control group through histological evaluation by HE staining and analysis of several biochemical indicators. Results showed that mice in DN group exhibited more severe renal injury and fibrosis, indicating successful establishment of the DN mouse model (Figure S1). To elucidate the effect of Smurf2 in DN, we first detect the mRNA expression and protein levels of Smurf2 in DN mouse model and MCs with HG treatment. Compared with the control group, the mRNA and protein level of Smurf2 in DN mice was significantly increased (*n* = 6, Figure 1(A–C)). Moreover, HG treatment markedly elevated Smurf2 mRNA expression and protein level in MCs (Figure 1(D–E)). In conclusion, we demonstrated that the expression of Smurf2 was increased in both DN mouse model and HG-treated MCs, indicating that Smurf2 may promote the development of DN.

### Smurf2 knockdown inhibits cell proliferation and fibrosis in HG-treated MCs

**Figure 2.**
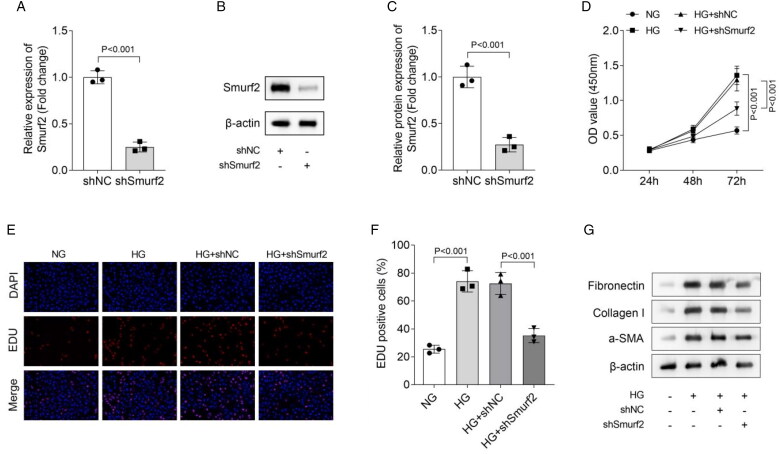
Smurf2 Knockdown inhibited cell proliferation and fibrosis in HG-treated MCs. (A) Smurf2 expression was measured by qPCR. (B and C) Smurf2 protein levels were detected by Western blot. (D) The cell viability was assessed using a CCK8 kit. (E and F) The cell proliferation was evaluated by EDU staining. (G) The protein levels of fibronectin, collagen I and α-SMA were measured using Western blot assay.

Next, we transfected shSmurf2 into MCs to investigate the function of Smurf2. Results showed that Smurf2 knockdown significantly inhibited Smurf2 mRNA expression and protein level in MCs (Figure 2(A–C)). Then, we measured cell viability of MCs. Results indicated that HG treatment promoted cell viability, which was reversed by Smurf2 knockdown (Figure 2(D)). EDU staining also suggested that HG significantly increased MCs proliferation, which was inhibited by Smurf2 knockdown (Figure 2(E,F)). Moreover, we measured the protein levels of fibrosis biomarkers fibronectin, Collagen I and α-SMA in MCs. We confirmed that HG upregulated the expression of fibronectin, Collagen I and α-SMA, which was partially restored by knockdown of Smurf2 (Figure 2(G)). These results indicated that Smurf2 knockdown restrained cell fibrosis in HG-treated MCs. In conclusion, we demonstrated that Smurf2 knockdown inhibited the proliferation and fibrosis in HG-treated MCs.

### Smurf2 knockdown increases EYA2 protein expression and stability through inhibiting EYA2 ubiquitination

**Figure 3.**
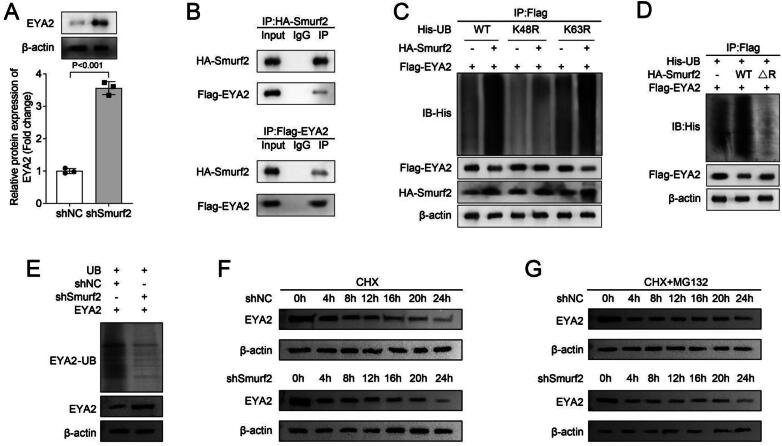
Smurf2 Knockdown increased EYA2 protein expression and stability through inhibiting EYA2 ubiquitination. (A) The protein level of EYA2 was measured by western blot assay. (B) IP was performed to evaluate the interaction between Smurf2 and EYA2. (C-E) EYA2 ubiquitination was assessed by Western blot and IP. (F and G) Western blot was performed to evaluate EYR protein stability after treatment of MCs with 10 μM CHX or 10 μM MG132 for 0, 8, 16, 24 h.

Through microarray analysis (results were not presented in this study), our previous research found that EYA2 expression was decreased in patients with DN. Therefore, we wondered whether Smurf2 mediated EYA2 stability through the ubiquitin-proteasome pathway. We first demonstrated that the protein level of EYA2 was upregulated in MCs with Smurf2 knockdown (Figure 3(A)). IP revealed the interaction between Smurf2 and EYA2 (Figure 3(B)). Then, we detected ubiquitination in MCs with Flag-EYA2, His-tagged ubiquitin wild-type (WT), or mutation plasmids (K48 or K63 mutants), and HA-Smurf2 or not. Results showed that Smurf2 promoted EYA2 ubiquitination in MCs, but downregulated the protein level of EYA2. Moreover, we confirmed that K48 mutation inhibited EYA2 ubiquitination (Figure 3(C)). These results indicated that EYA2 was the substrate of Smurf2. In addition, we demonstrated that the mutation of Smurf2 RING domain (ΔR) inhibited the ubiquitination of EYA2, suggesting Smurf2 acted as an E3 ubiquitin ligase to mediate ubiquitination of EYA2 (Figure 3(D)). Additionally, Smurf2 knockdown inhibited EYA2 ubiquitination but upregulated the protein level of EYA2 (Figure 3(E)). This evidence further corroborated that Smurf2 mediates the ubiquitination of EYA2. Subsequently, we detected the protein stability of EYA2 in MCs. Compared with the shNC group, Smurf2 knockdown inhibited EYA2 protein degradation (Figure 3(F)), while CHX and MG132 co-treatment not affected the protein levels of EYA2 at different times (Figure 3(G)). Taken together, we demonstrated that Smurf2 knockdown inhibited EYA2 ubiquitination to promote EYA2 protein stability, thereby upregulating the protein level of EYA2.

### EYA2 knockdown reverses the inhibition of cell proliferation and fibrosis in HG-treated MCs induced by Smurf2 knockdown

**Figure 4.**
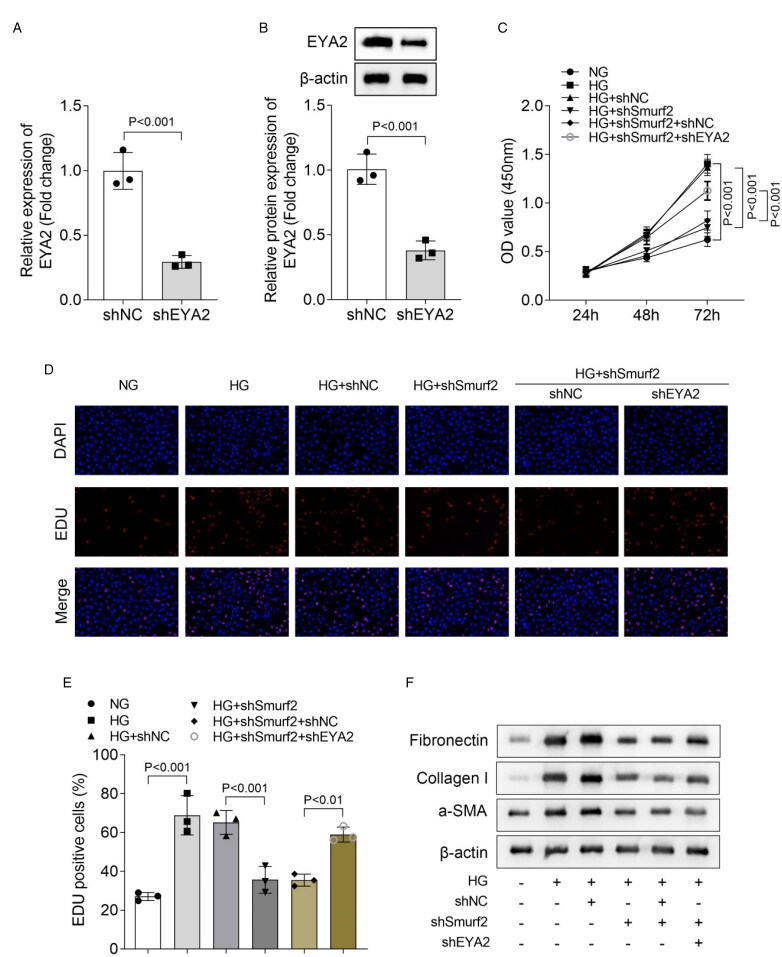
EYA2 Knockdown reversed the inhibition of cell proliferation and fibrosis in HG-treated MCs with Smurf2 knockdown. (A) The expression of EYA2 was measured by qPCR. (B) The protein levels of EYA2 were detected by Western blot. (C) The cell viability was assessed using a CCK8 kit. (D and E) The cell proliferation was evaluated by EDU staining. (F) The protein levels of fibronectin, collagen I and α-SMA were measured using Western blot assay.

Then, the function of EYA2 was confirmed through rescue experiments. The mRNA expression and protein level of EYA2 in MCs was decreased through transfection of MCs (Figure 4(A,B)). We found that EYA2 knockdown increased cell viability in HG-treated MCs with Smurf2 knockdown (Figure 4(C)). Moreover, cell proliferation inhibited by Smurf2 knockdown in HG-treated MCs was restored by knockdown of EYA2 (Figure 4(D,E)). In addition, the protein levels of fibrosis biomarkers fibronectin, Collagen I and α-SMA were upregulated by knockdown of EYA2 in HG-treated MCs with Smurf2 knockdown (Figure 4(F)). Furthermore, we performed these experiments on HG-treated human glomerular MCs, and found that knockdown of EYA2 reversed the cell fibrosis and cell proliferation inhibited by Smurf2 knockdown in the cells (Figure S2). These results indicated that EYA2 knockdown reversed the inhibition of cell proliferation and cell fibrosis in HG-treated MCs induced by Smurf2 knockdown.

### Smurf2 knockdown relieves the injury and fibrosis of kidney in DN mouse model

**Figure 5.**
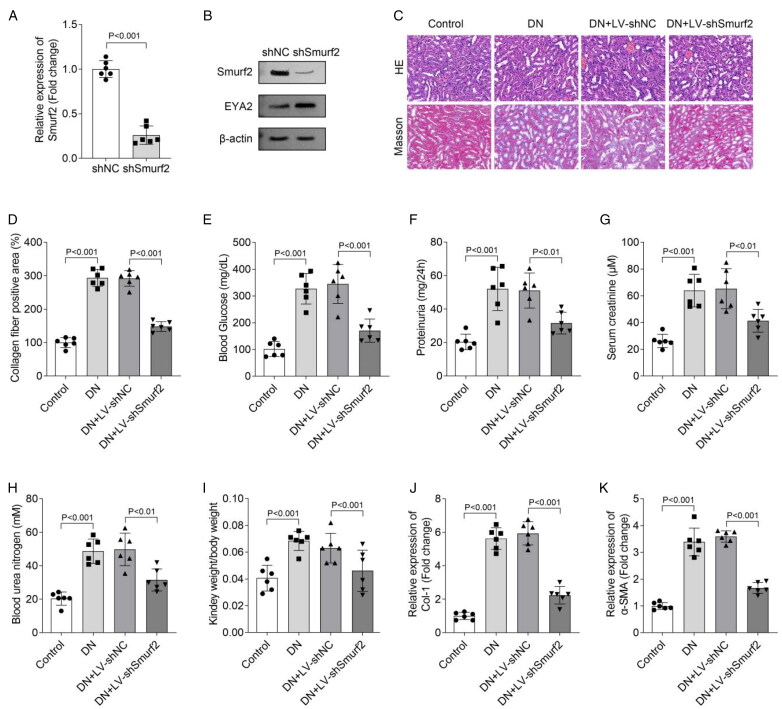
Smurf2 Knockdown relieved the injury and fibrosis of kidney in DN mouse model. (A) Smurf2 expression in kidney of mice was measured by qPCR. (B) The protein levels of Smurf2 and EYA2 were detected by Western blot. (C) HE staining and masson staining were performed to evaluate pathological changes in the kidney. (D) Quantification of the collagen fiber-positive area. (E) The blood glucose was measured using a glucose assay kit. (F) The proteinuria was measured using an urine protein content assay kit. (G) The serum creatinine was evaluated by a creatinine assay kit. (H) The blood urea nitrogen was measured using an urea nitrogen assay kit. (I) The relative kidney weight was calculated by kidney weight/body weight. (J and K) qPCR was performed to measure the expression of Col-1 and α-SMA in kidney of mice.

Finally, we investigated the function of Smurf2 in DN mice. Results showed that the expression of Smurf2 *in vivo* was significantly downregulated after Smurf2 knockdown (Figure 5A). Moreover, western blot suggested that Sumrf2 knockdown reduced the protein level of Sumrf2 but upregulated the protein level of EYA2 (Figure 5(B)). HE staining suggested that the glomerular lesions were aggravated in DN mouse model, while it was relieved with Smurf2 knockdown. Moreover, Masson staining showed that DN mice displayed more fibrosis than that in the control group, while it was partially restored by Smurf2 knockdown (Figure 5(C,D)). In addition, we measured several biochemical parameters in mice, including the blood glucose, proteinuria, serum creatinine, blood urea nitrogen, and the relative kidney weight. Compared with the control group, these parameters were all significantly increased in DN mice, while the elevation was partially inhibited by Smurf2 knockdown (*n* = 6, Figure 5(E–I)). Moreover, qPCR suggested that Col-1 and α-SMA mRNA expression was significantly increased in DN mice, which was reduced by Smurf2 knockdown (Figure 5(J,K)). In conclusion, we demonstrated that Smurf2 knockdown relieved kidney injury in DN mouse model.

## Discussion

MCs proliferation and fibrosis are main characteristics of DN, and many studies have studied its mechanism from the perspective of genes. For example, Kim et al. [30] reported that lysophosphatidic acid increases MCs proliferation in DN *via* Rac1/MAPK/KLF5 signaling. Ge et al. [31] revealed that circ_0000064 promotes the proliferation and fibrosis of MCs *via* miR-143 in DN. In addition, some studies have reported the effects of protein modifications, such as ubiquitination, on the development of DN. For example, Chen et al. [15] demonstrated that Ginkgolide B improves DN through inhibiting GPX4 ubiquitination. Li et al. [32] reported that altered DNA methylation of TRIM13 in DN suppresses mesangial collagen synthesis by promoting ubiquitination of CHOP. Liu et al. [33] revealed that TIPE1 promotes the ubiquitination and proteasomal degradation of PHB2, destabilizing PHB2 and thereby exacerbating renal tubular injury, which in turn aggravates diabetic nephropathy. Moreover, Zhao et al. [34] demonstrated that podocyte OTUD5 alleviates DN through deubiquitinating TAK1 and reducing podocyte inflammation and injury. Smurf2, a member of E3 ubiquitin ligases, is also involved in the progression of DN. The effect of smurf2 on the degradation of SnoN by ubiquitination has been demonstrated to be a key factor in DN [35]. Few studies investigate the relationship between Smurf2 and biological behaviors of MCs in DN, only Kim et al. [23] revealed that inhibition of ChREBP ubiquitination *via* downregulation of Smurf2 contributes to lysophosphatidic acid-induced fibrosis in renal MCs. In this study, we found that Smurf2 expression was increased in DN mouse model and HG-treated MCs. At the cellular level, knockdown of Smurf2 inhibited cell viability and proliferation, as well as the protein levels of fibrosis biomarkers in HG-treated MCs. In DN mouse model, Smurf2 knockdown inhibited renal fibrosis in mice and reduced the contents of blood glucose, proteinuria, serum creatinine, blood urea nitrogen and the relative kidney weight. We speculated that the improvement of kidney function was caused by Smurf2 knockdown-induced decreased blood glucose, but there was no direct evidence to support our suppose, which need to be explored in the future work.

In recent years, researches about EYA2 mainly focus on its regulation of cancers. For instance, Liu et al. [25] demonstrated that EYA2 suppresses the progression of hepatocellular carcinoma *via* SOCS3-mediated blockade of JAK/STAT signaling. Wolin et al. [36] revealed that inhibition of EYA2 prevents medulloblastoma progression. Shen et al. [37] proved that ubiquitin ligase SCFFBXW7 promotes EYA2 degradation thus enhancing antitumor immune responses. However, whether EYA2 regulates DN progression remains unclear, and whether Smurf2 mediates EYA2 ubiquitination has not been reported. In our present study, we first demonstrated that Smurf2 knockdown increased EYA2 protein expression and stability through inhibiting EYA2 ubiquitination. Moreover, EYA2 knockdown reversed the inhibition of cell proliferation and fibrosis induced by Smurf2 knockdown in HG-treated MCs, suggesting that EYA2 knockdown may promote the development of DN by promoting cell proliferation and fibrosis of MCs.

However, limitations still exists in our study. While phenotypic consistency was confirmed using multiple independent shRNA sequences, systematic validation of potential off-target effects was not performed. Future studies should incorporate CRISPR/Cas9-based gene editing or transcriptome-wide sequencing to exclude off-target interference and strengthen the reliability of conclusions.

In conclusion, we revealed that Smurf2 knockdown inhibited MC proliferation and fibrosis through suppressing EYA2 ubiquitination, thereby alleviating the progression of DN. Additionally, the mechanisms by which Smurf2 regulates DN may involve multiple aspects, including direct regulation of MC cycle and fibrosis-related signaling pathways, or systemic metabolic regulation that impacts the overall pathological progression of DN. These mechanisms are not limited to the regulation of EYA2 stability *via* ubiquitination by Smurf2. Targeting the inhibition of Smurf2 expression may be pivotal in treating DN. Additionally, directly enhancing EYA2 function or preventing its ubiquitination and degradation could represent another therapeutic avenue, particularly for DN patients with hyperactivated Smurf2. Therefore, agents targeting Smurf2, such as specific inhibitors or monoclonal antibodies, might become a breakthrough in the precision treatment of DN, especially for patients not adequately responding to conventional glucose control therapies. Future research should focus on elucidating the expression patterns of the Smurf2-EYA2 axis in human DN tissues and validate its potential as a biomarker or therapeutic target through clinical trials, ultimately facilitating the application of personalized anti-fibrotic therapies in DN management.

## Supplementary Material

figures (1).zip

## Data Availability

The datasets used and/or analyzed during the current study are available from the corresponding author on reasonable request.
