# Supplementary figures and images for: Smurf2 knockdown attenuates the progression of diabetic nephropathy by inhibiting mesangial cell proliferation and fibrosis through suppressing EYA2 ubiquitination
Source: Ren Fail. 2025 Jun 24;47(1):2520904. doi: 10.1080/0886022X.2025.2520904 (PMC12893500; doi:10.1080/0886022X.2025.2520904)

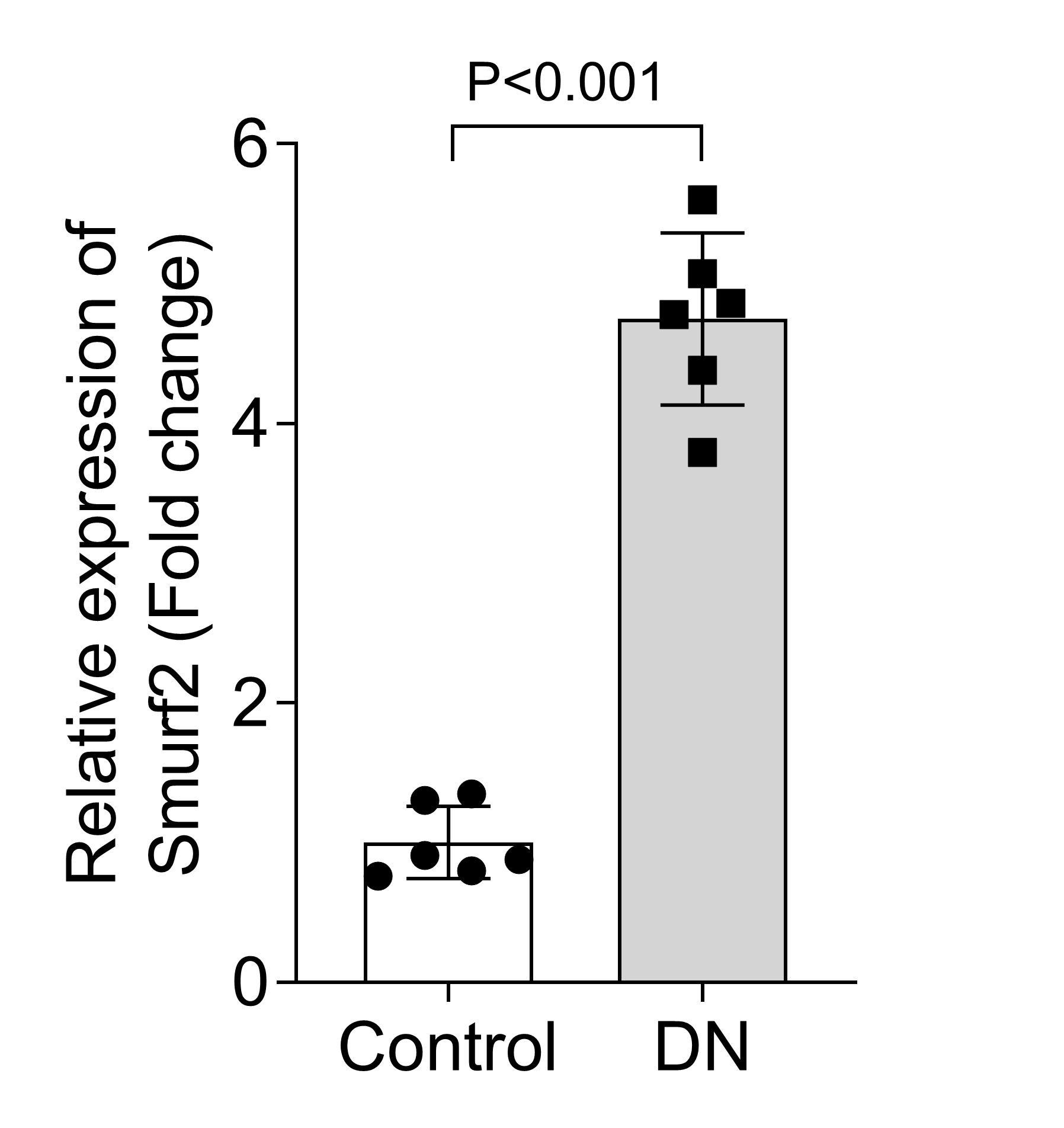

Supplement: figures (1).zip [file IRNF_A_2520904_SM9339.zip › Fig.1/1A.tif]

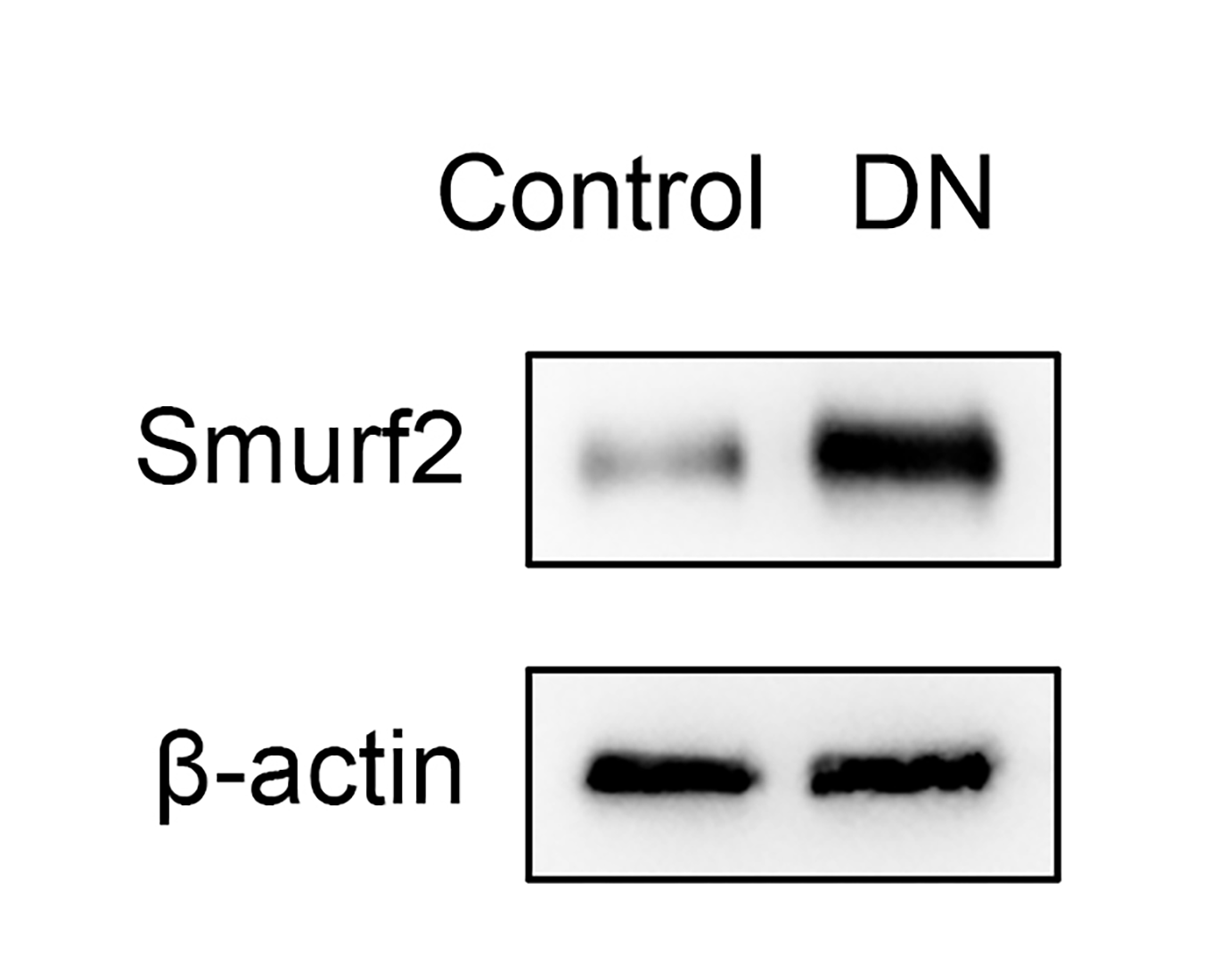

Supplement: figures (1).zip [file IRNF_A_2520904_SM9339.zip › Fig.1/1B.tif]

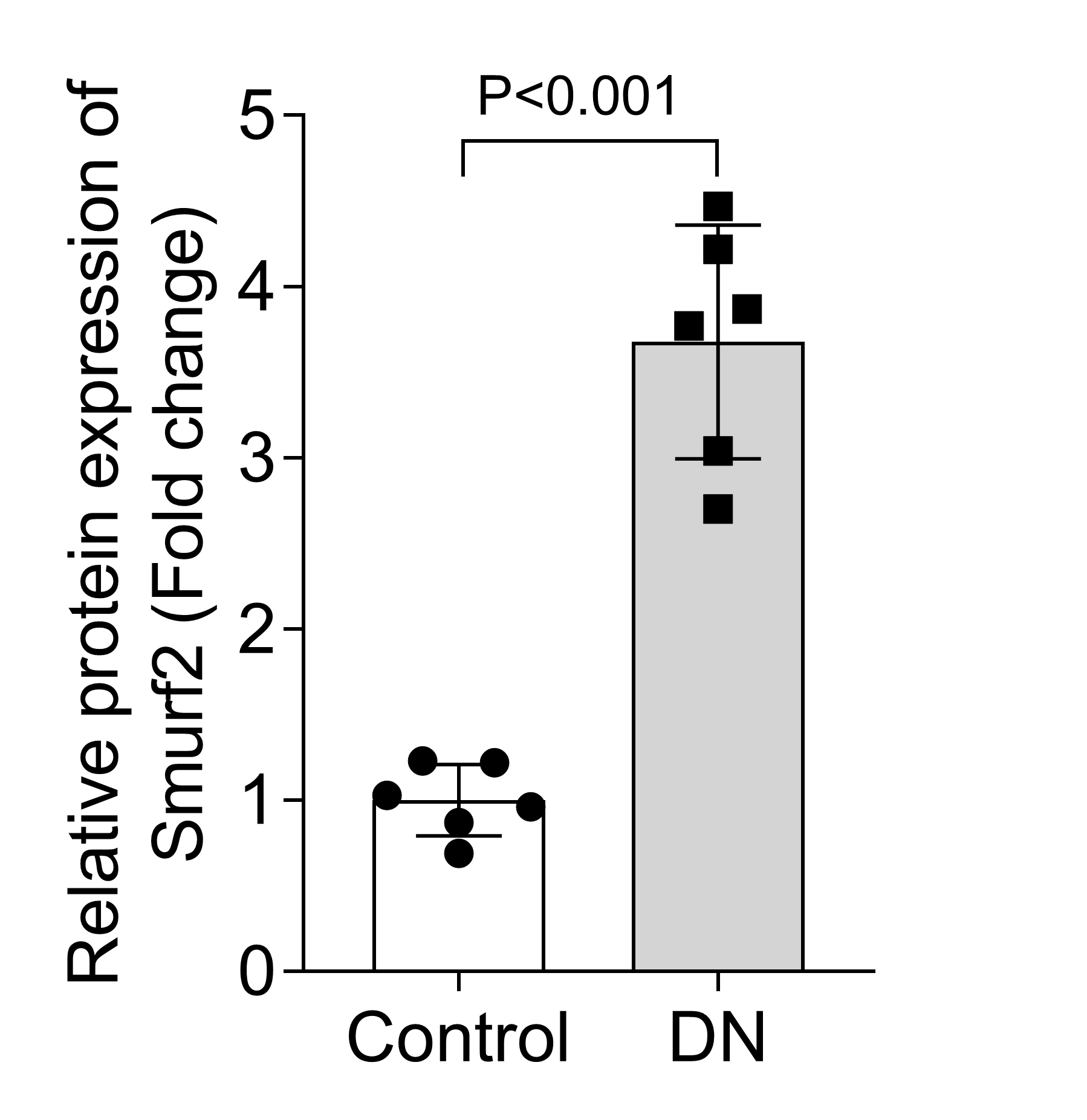

Supplement: figures (1).zip [file IRNF_A_2520904_SM9339.zip › Fig.1/1C.tif]

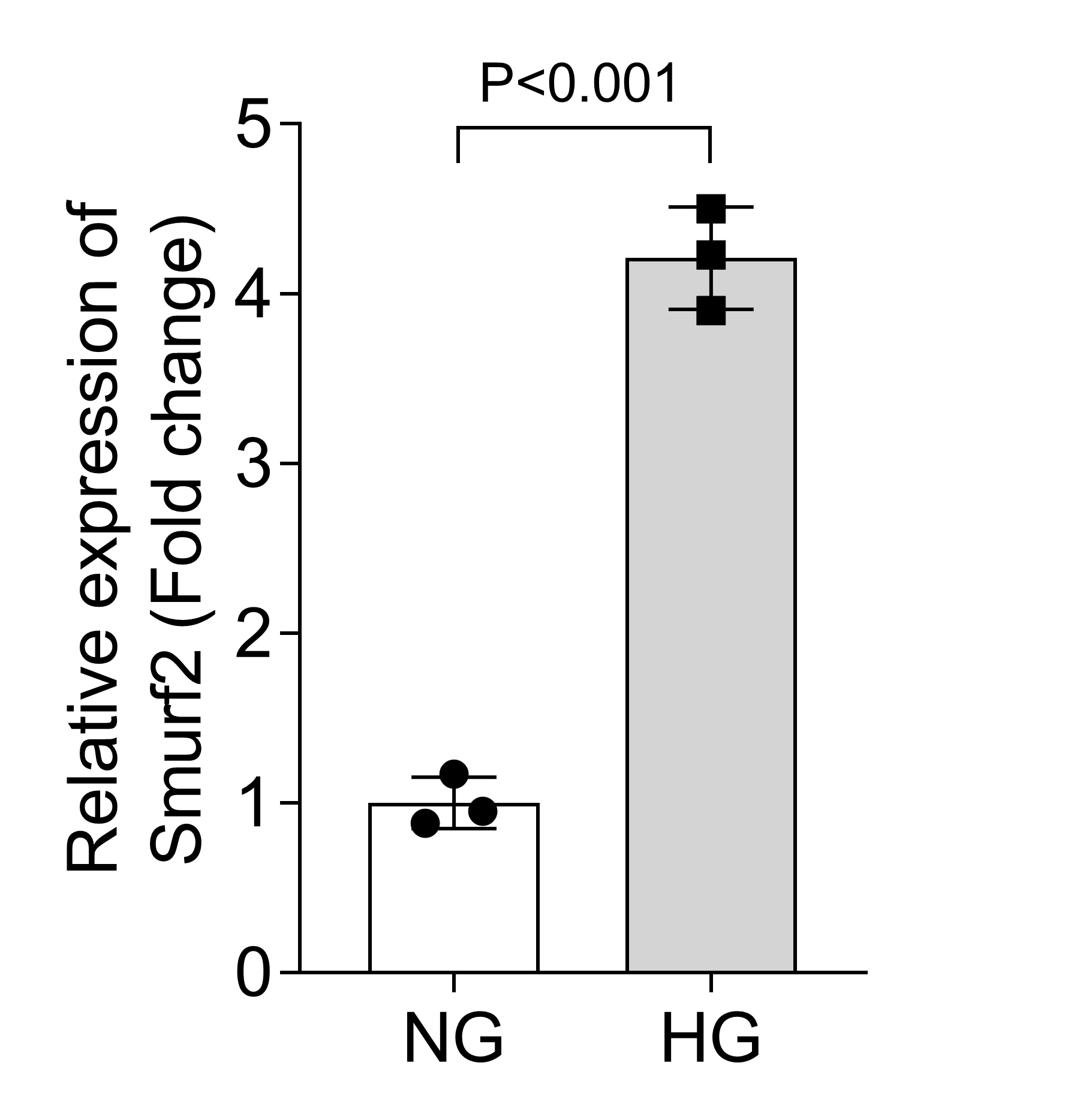

Supplement: figures (1).zip [file IRNF_A_2520904_SM9339.zip › Fig.1/1D.tif]

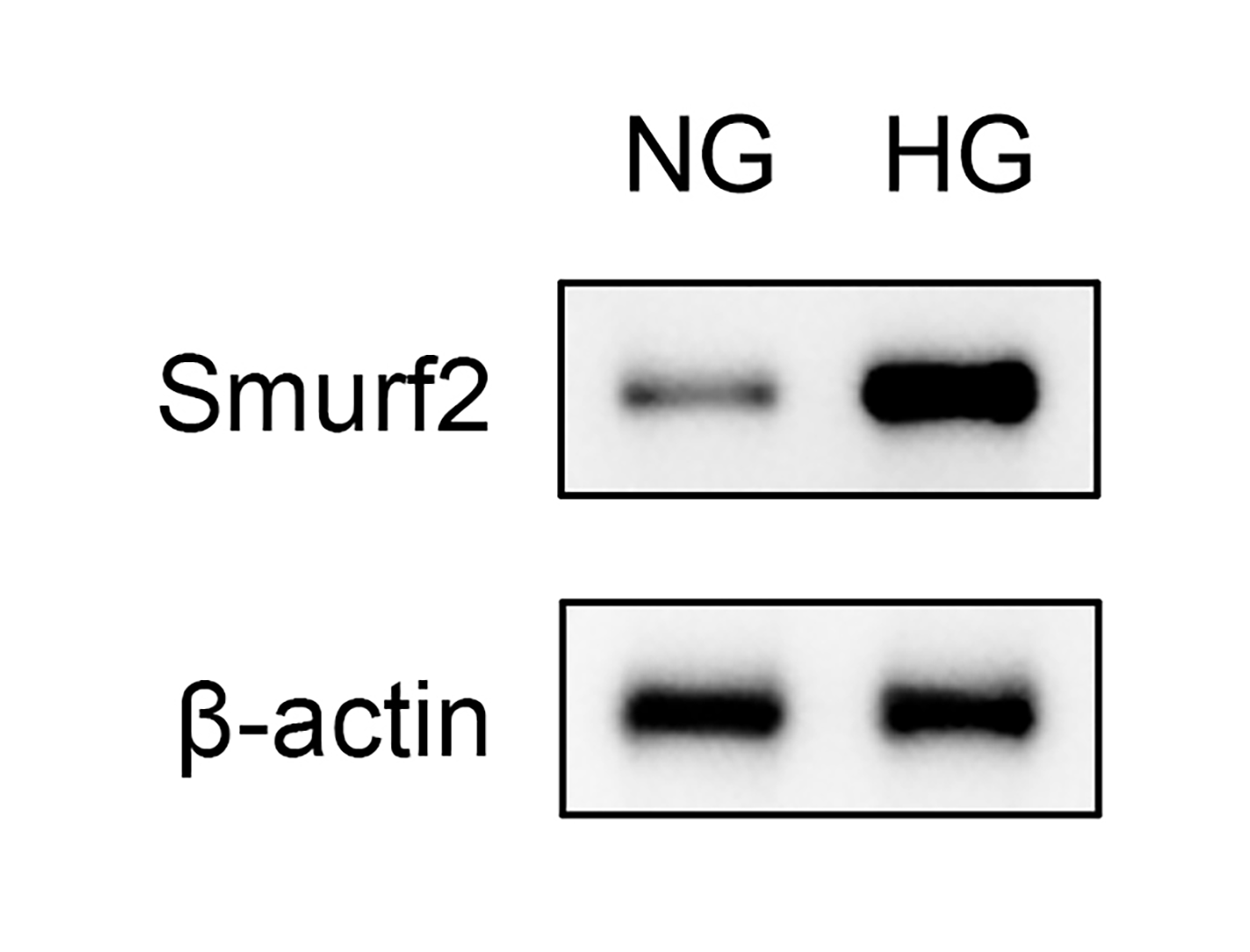

Supplement: figures (1).zip [file IRNF_A_2520904_SM9339.zip › Fig.1/1E.tif]

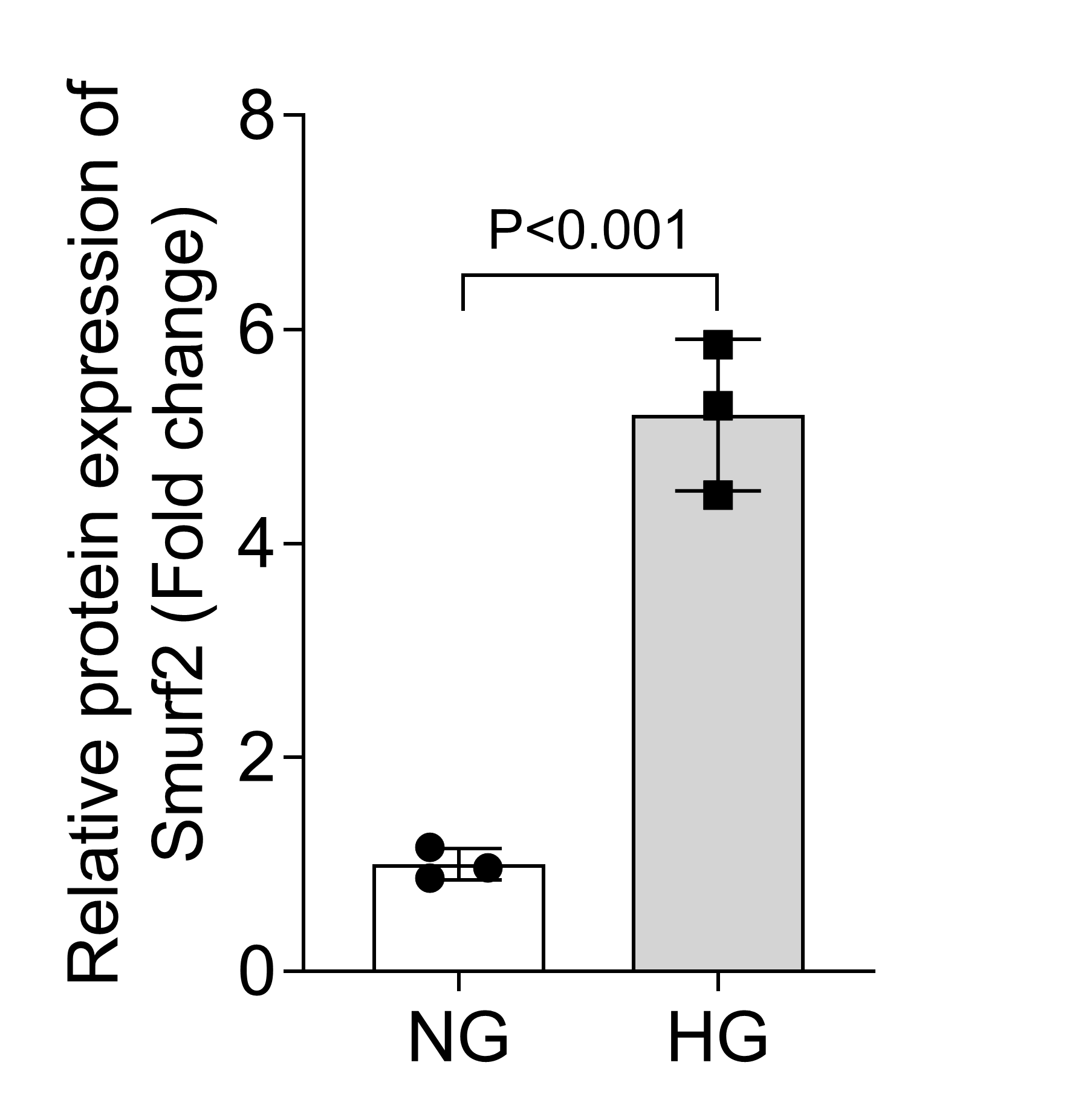

Supplement: figures (1).zip [file IRNF_A_2520904_SM9339.zip › Fig.1/1F.tif]

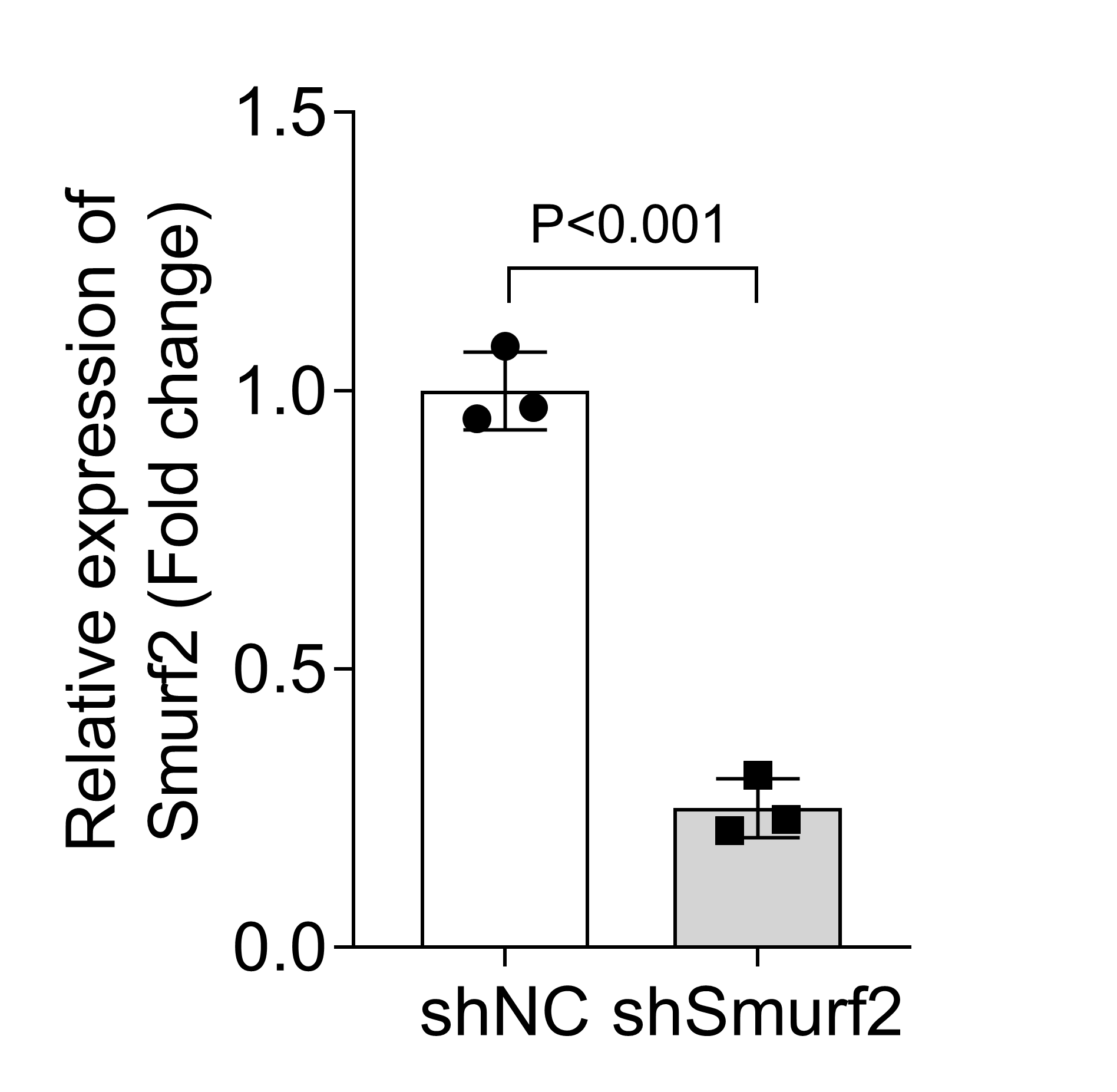

Supplement: figures (1).zip [file IRNF_A_2520904_SM9339.zip › Fig.2/2A.tif]

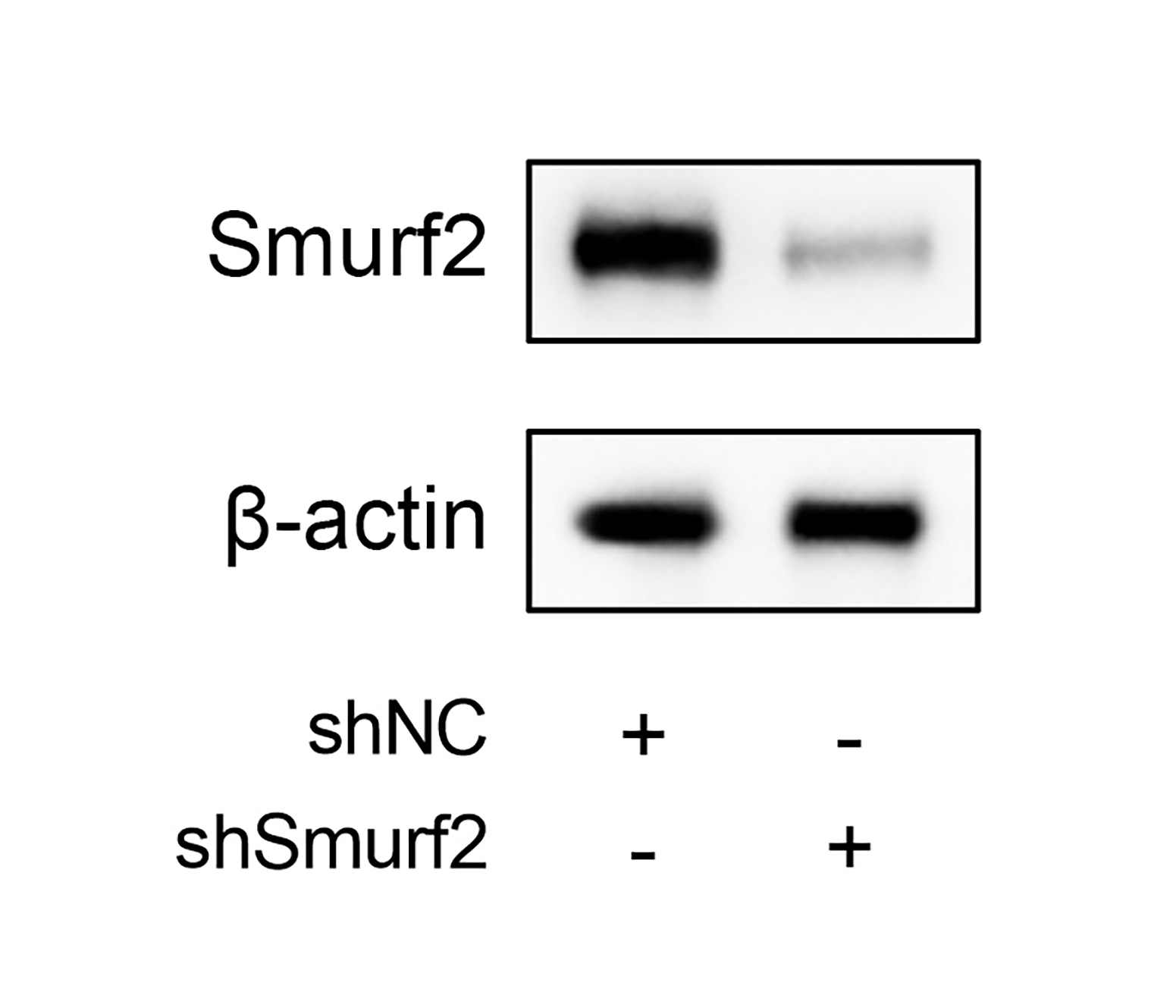

Supplement: figures (1).zip [file IRNF_A_2520904_SM9339.zip › Fig.2/2B.tif]

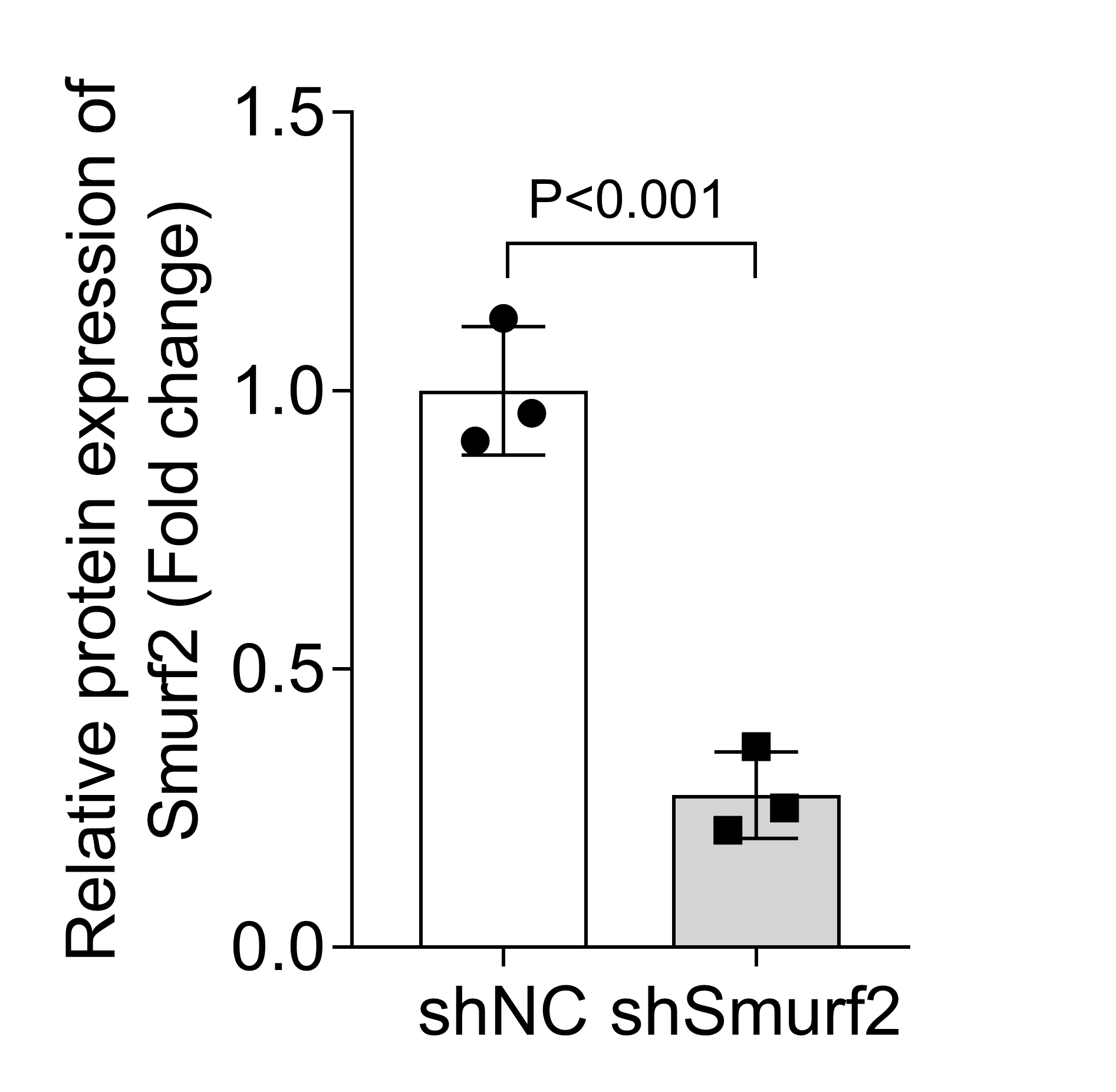

Supplement: figures (1).zip [file IRNF_A_2520904_SM9339.zip › Fig.2/2C.tif]

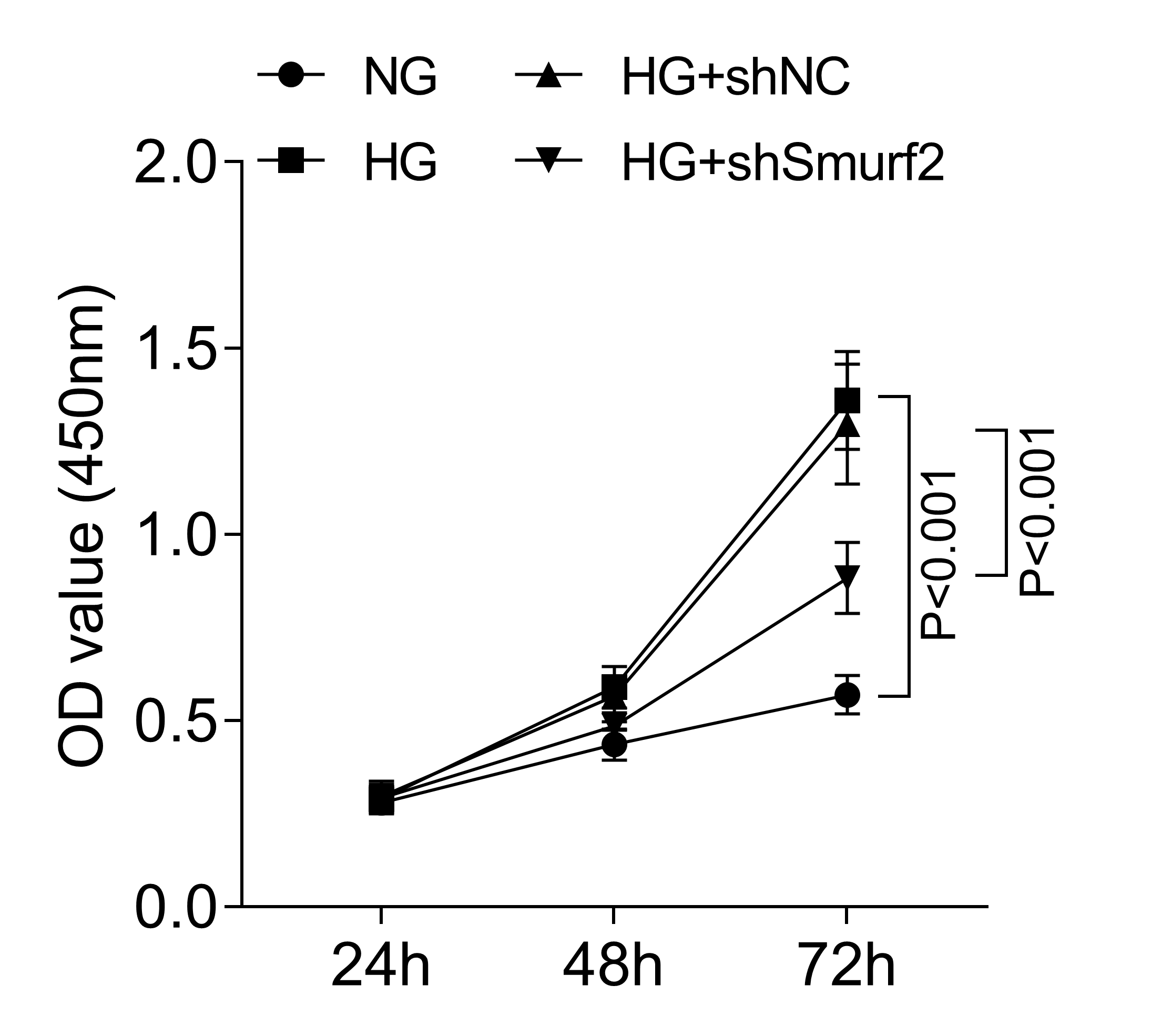

Supplement: figures (1).zip [file IRNF_A_2520904_SM9339.zip › Fig.2/2D.tif]

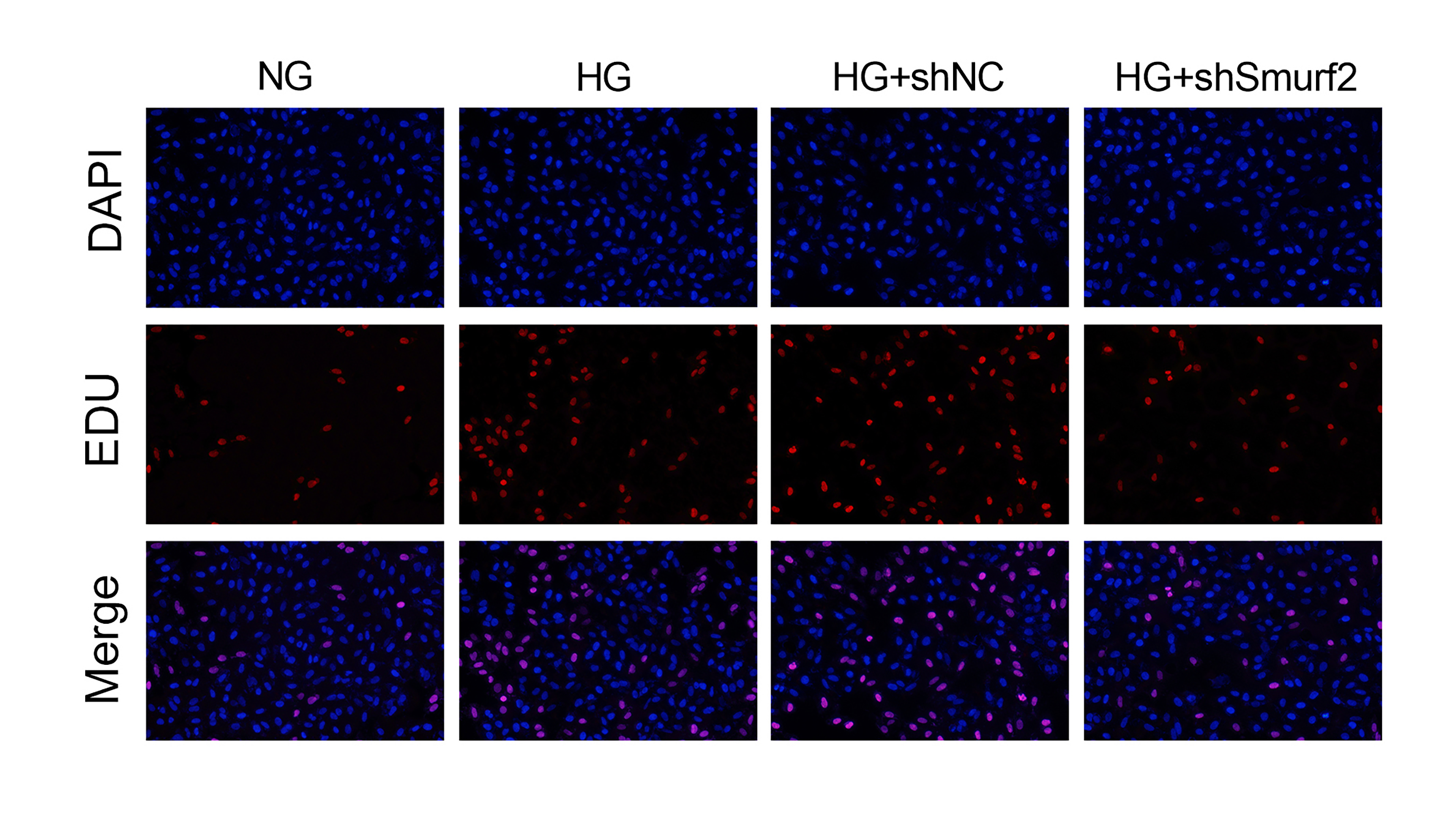

Supplement: figures (1).zip [file IRNF_A_2520904_SM9339.zip › Fig.2/2E.tif]

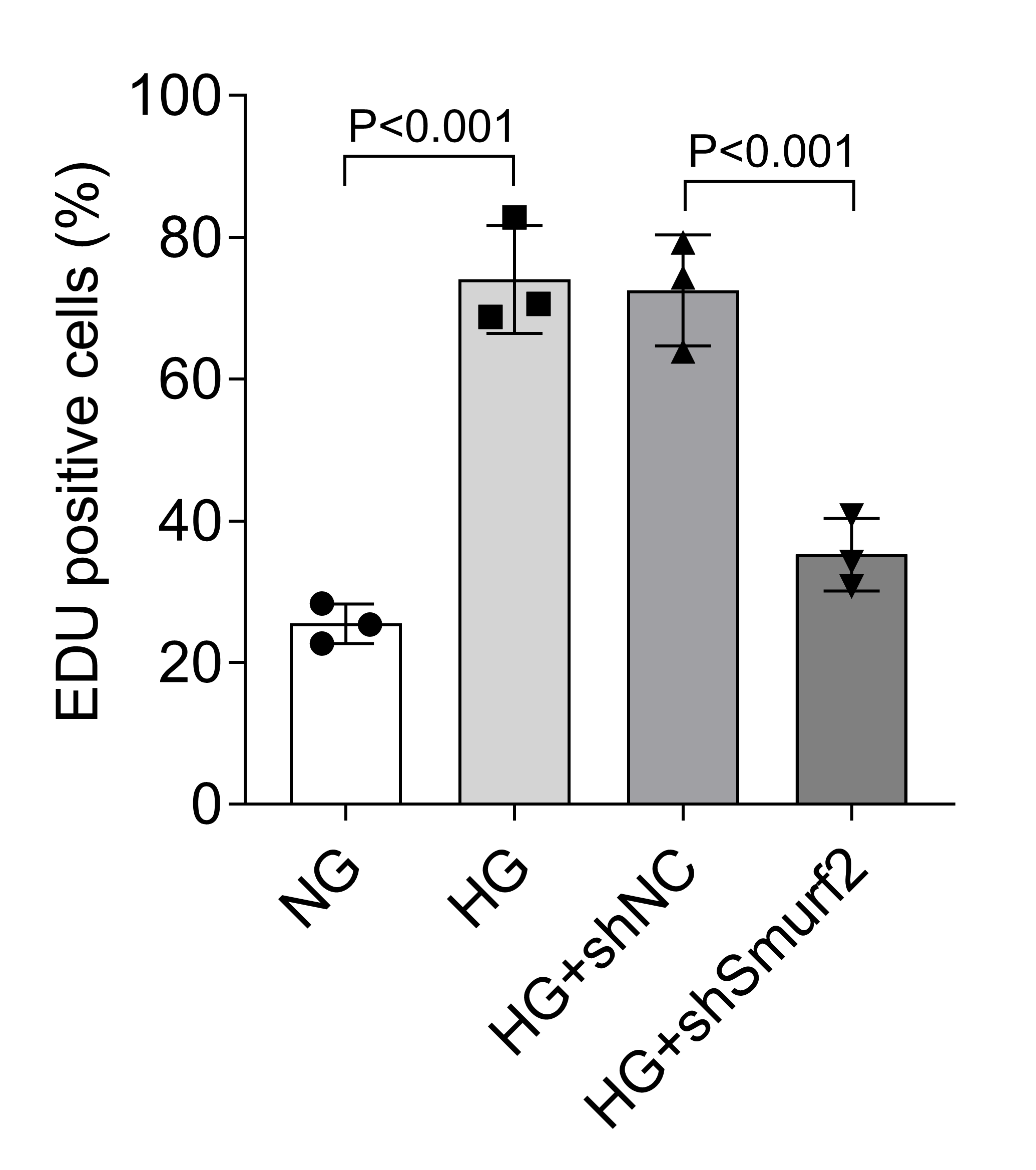

Supplement: figures (1).zip [file IRNF_A_2520904_SM9339.zip › Fig.2/2F.tif]

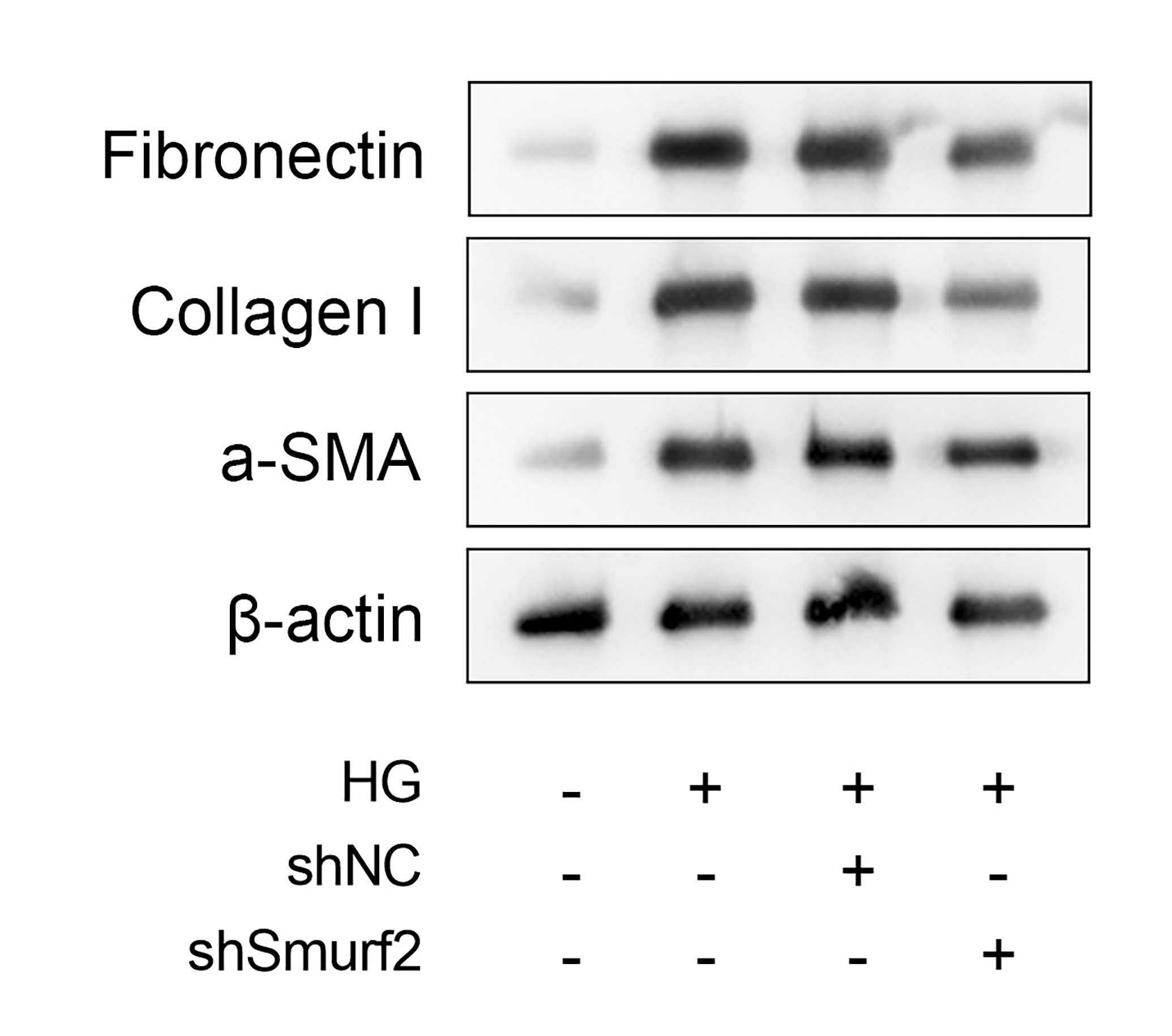

Supplement: figures (1).zip [file IRNF_A_2520904_SM9339.zip › Fig.2/2G.tif]

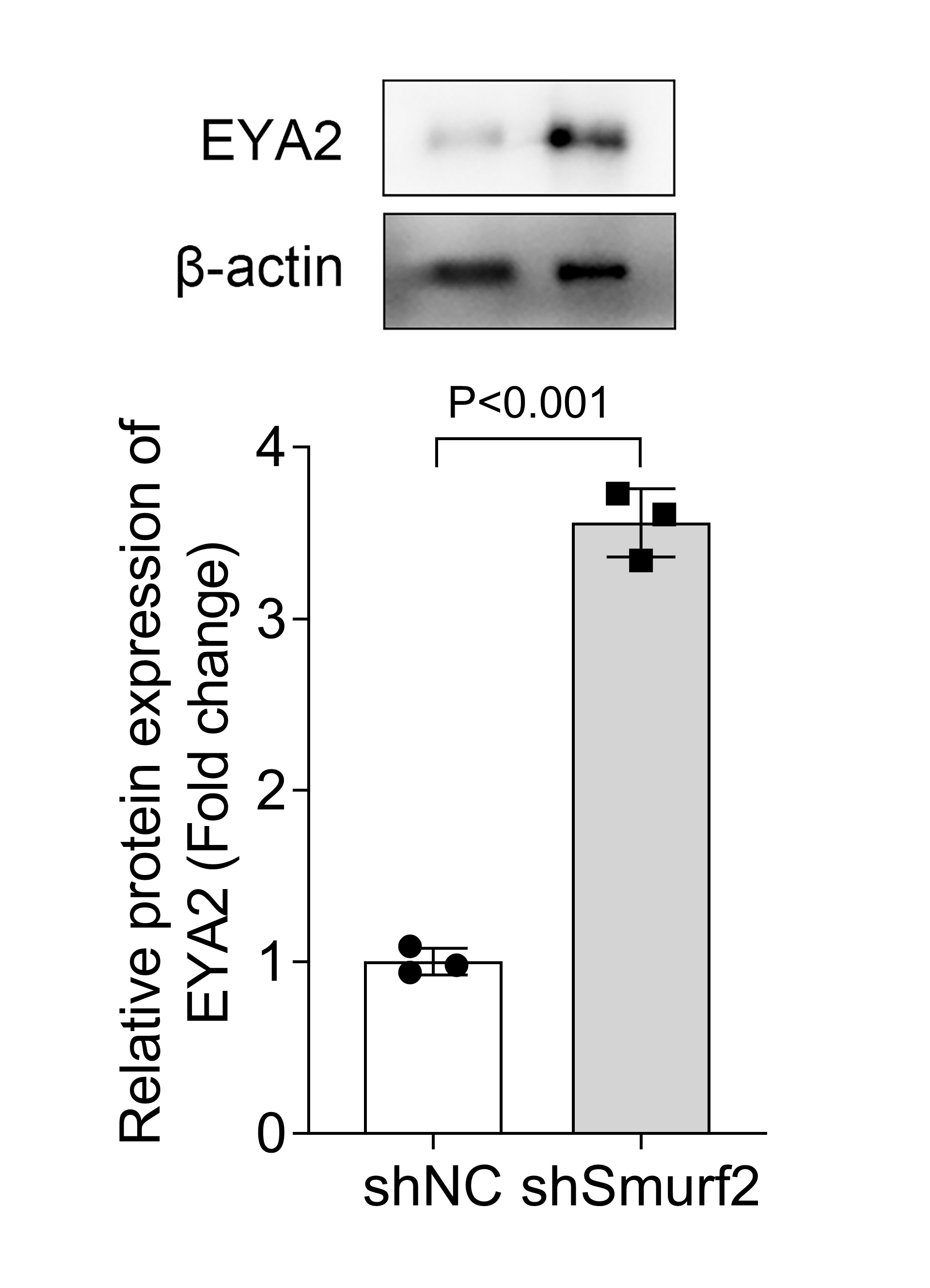

Supplement: figures (1).zip [file IRNF_A_2520904_SM9339.zip › Fig.3/3A.tif]

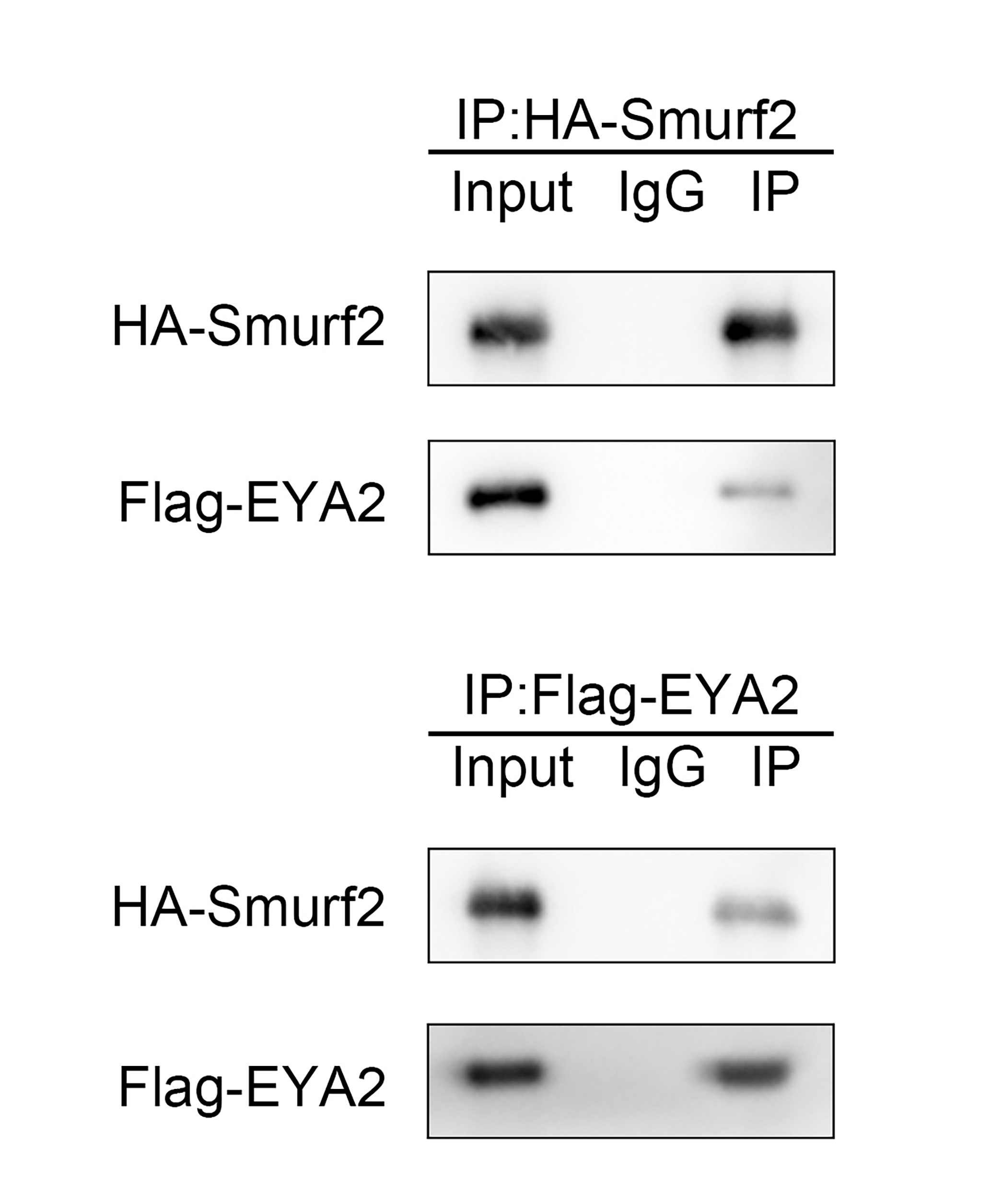

Supplement: figures (1).zip [file IRNF_A_2520904_SM9339.zip › Fig.3/3B.tif]

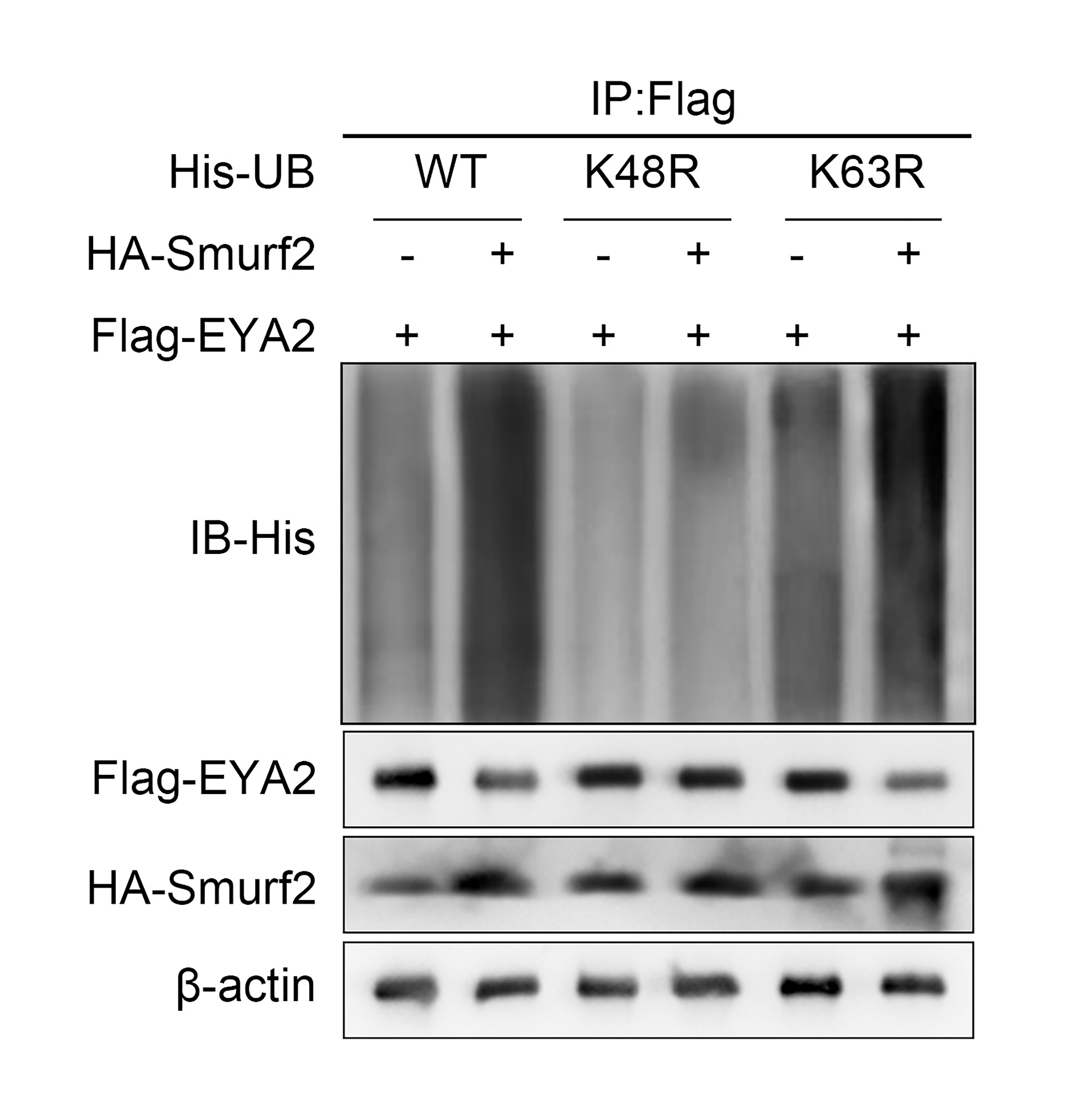

Supplement: figures (1).zip [file IRNF_A_2520904_SM9339.zip › Fig.3/3C.tif]

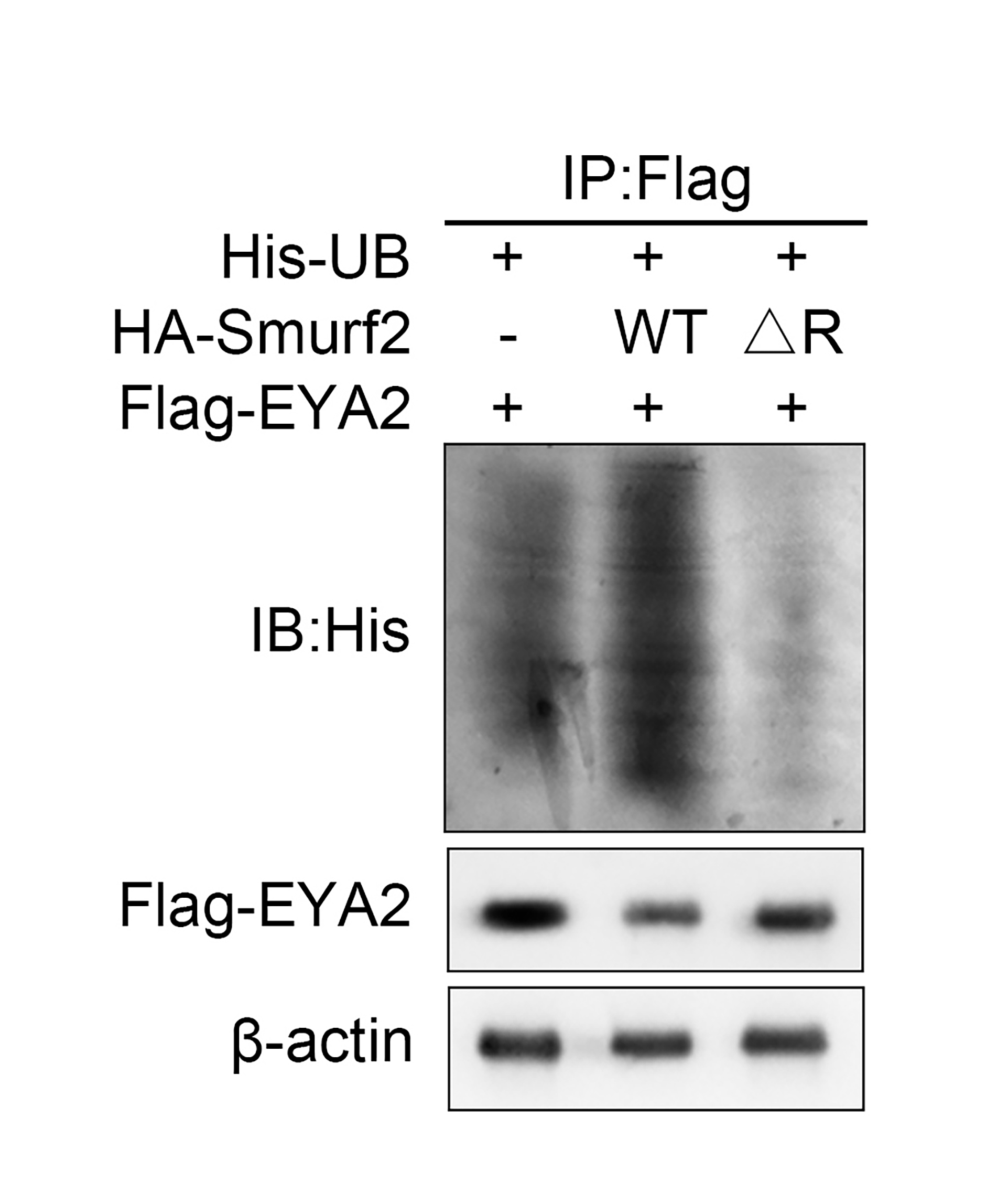

Supplement: figures (1).zip [file IRNF_A_2520904_SM9339.zip › Fig.3/3D.tif]

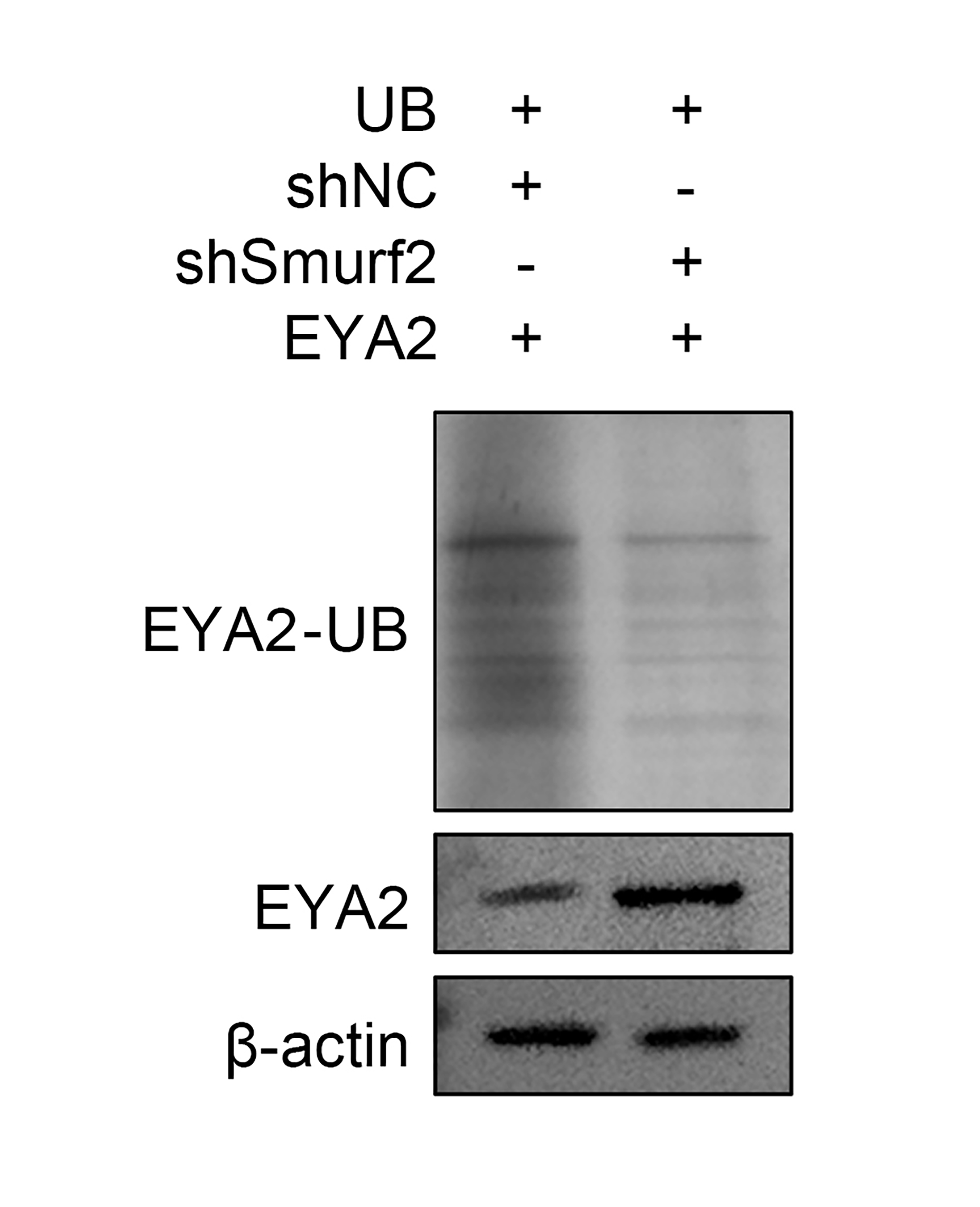

Supplement: figures (1).zip [file IRNF_A_2520904_SM9339.zip › Fig.3/3E.tif]

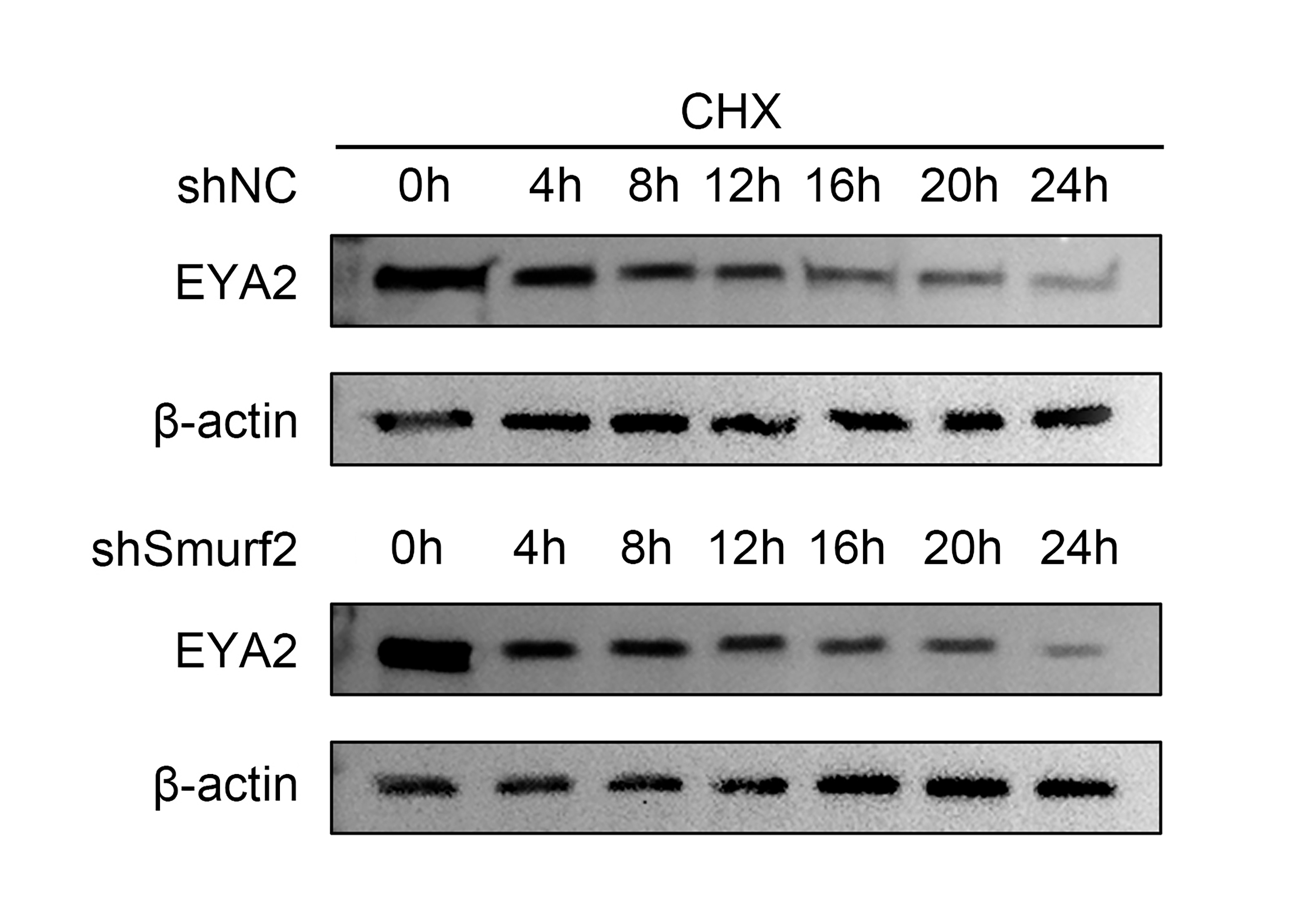

Supplement: figures (1).zip [file IRNF_A_2520904_SM9339.zip › Fig.3/3F.jpg]

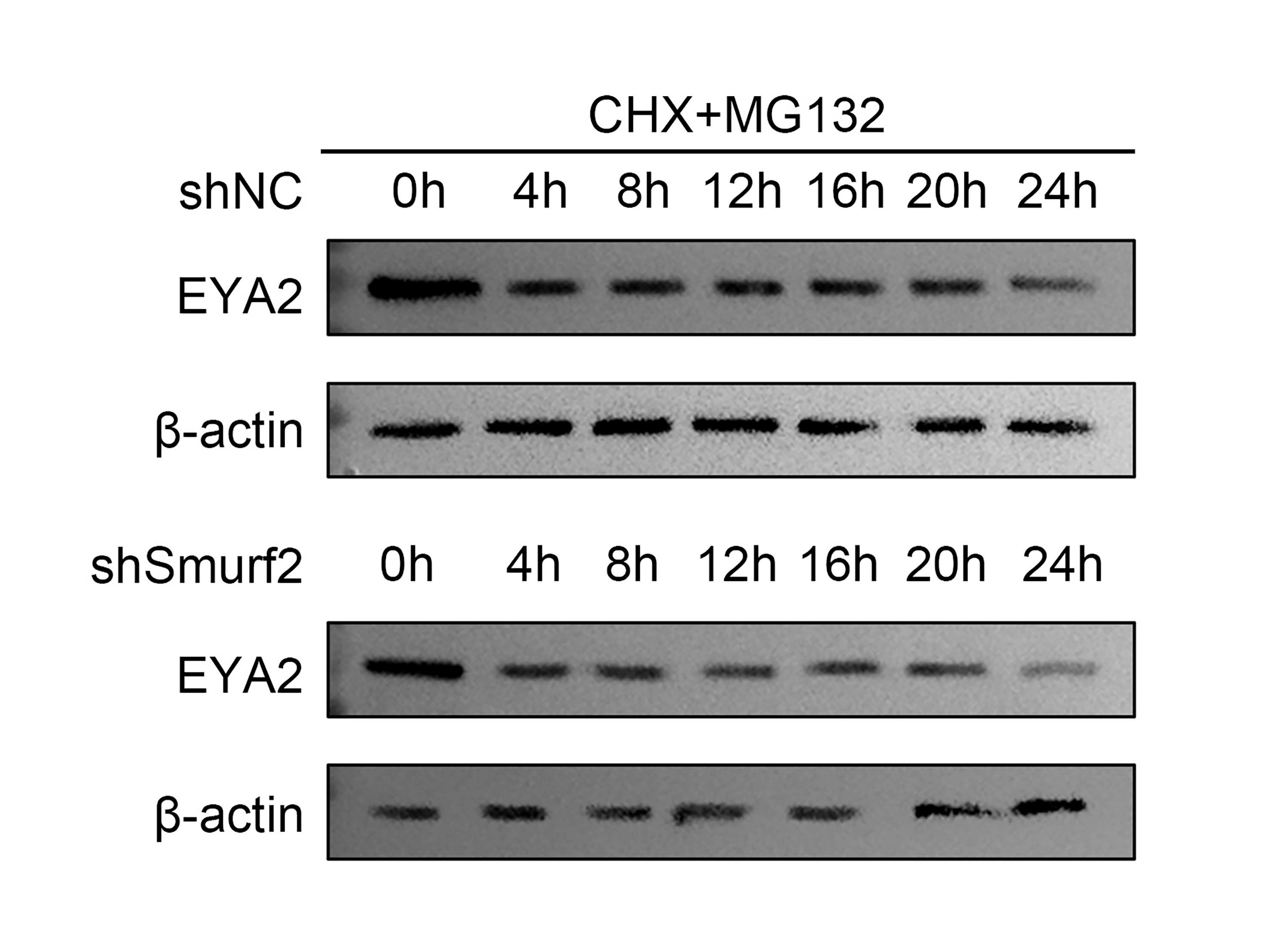

Supplement: figures (1).zip [file IRNF_A_2520904_SM9339.zip › Fig.3/3G.jpg]

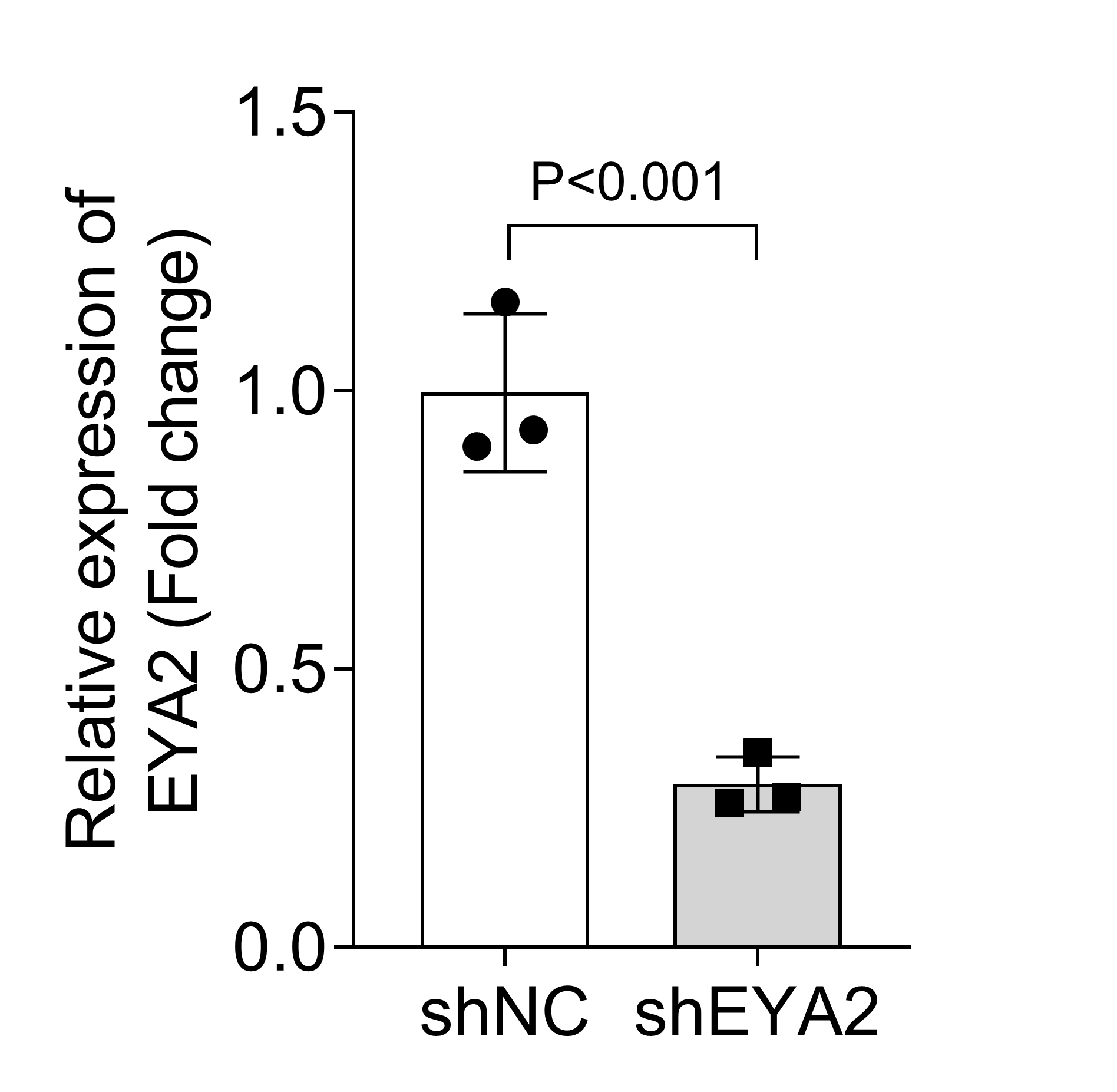

Supplement: figures (1).zip [file IRNF_A_2520904_SM9339.zip › Fig.4/4A.tif]

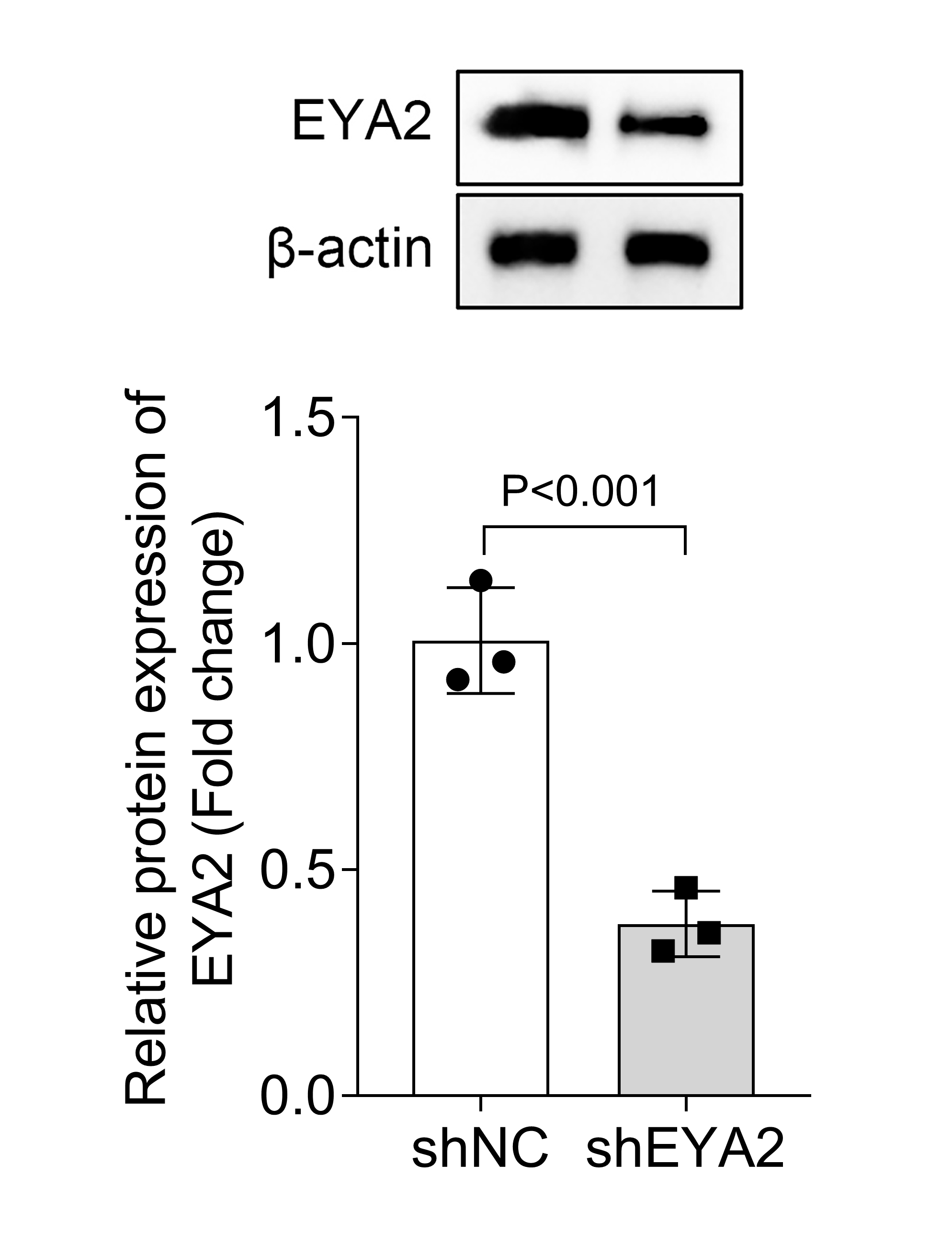

Supplement: figures (1).zip [file IRNF_A_2520904_SM9339.zip › Fig.4/4B.tif]

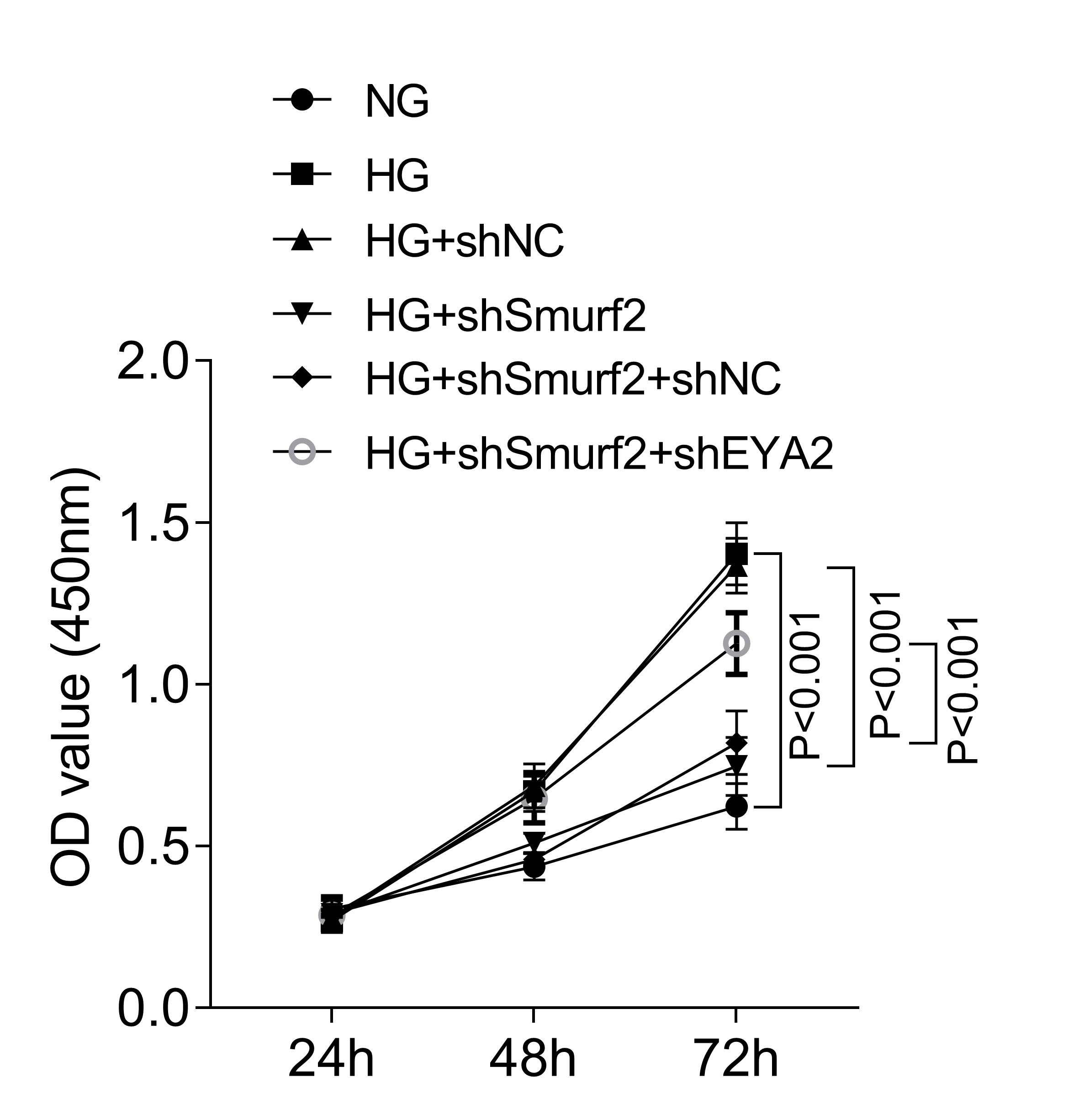

Supplement: figures (1).zip [file IRNF_A_2520904_SM9339.zip › Fig.4/4C.tif]

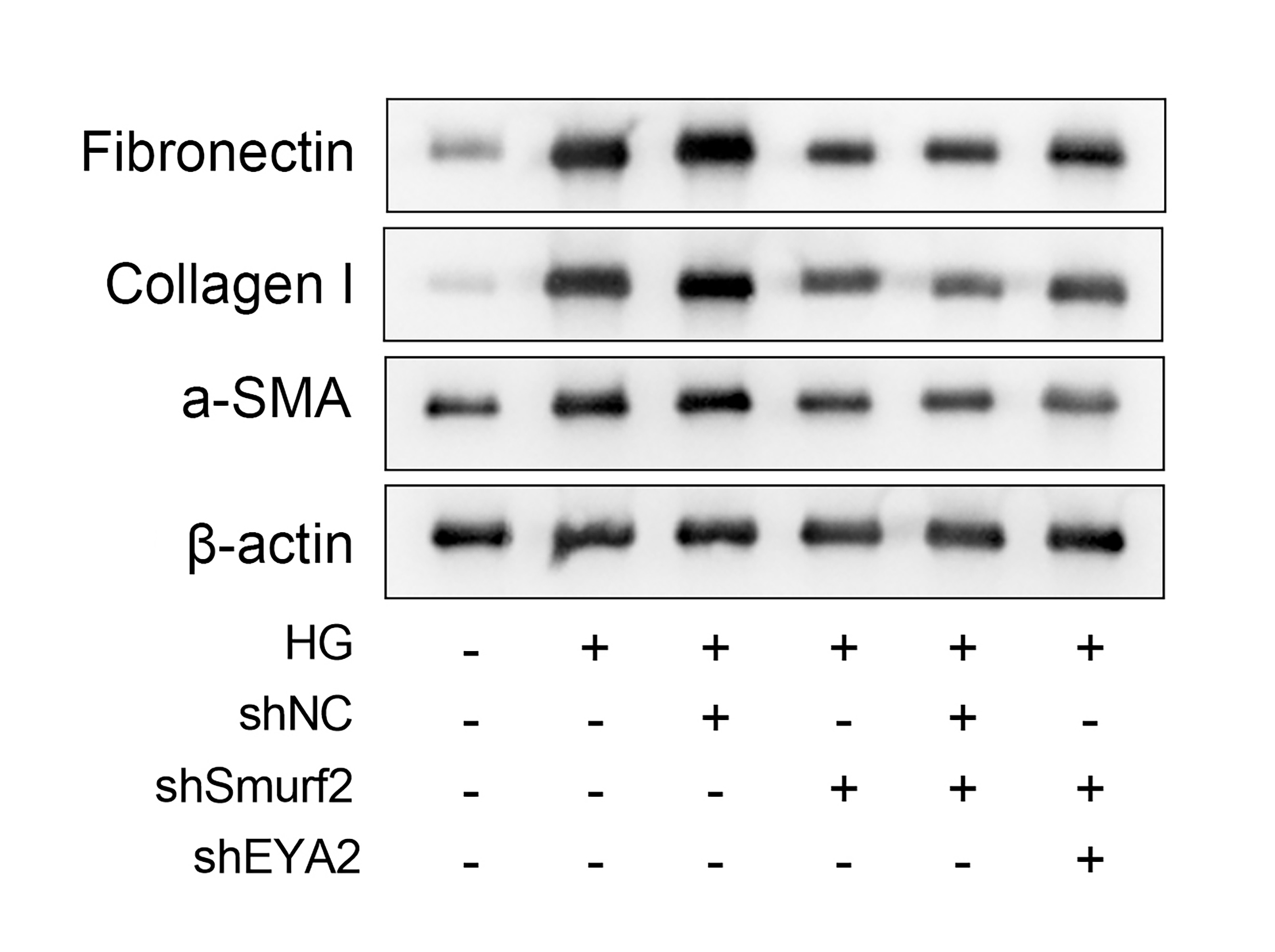

Supplement: figures (1).zip [file IRNF_A_2520904_SM9339.zip › Fig.4/4D.tif]

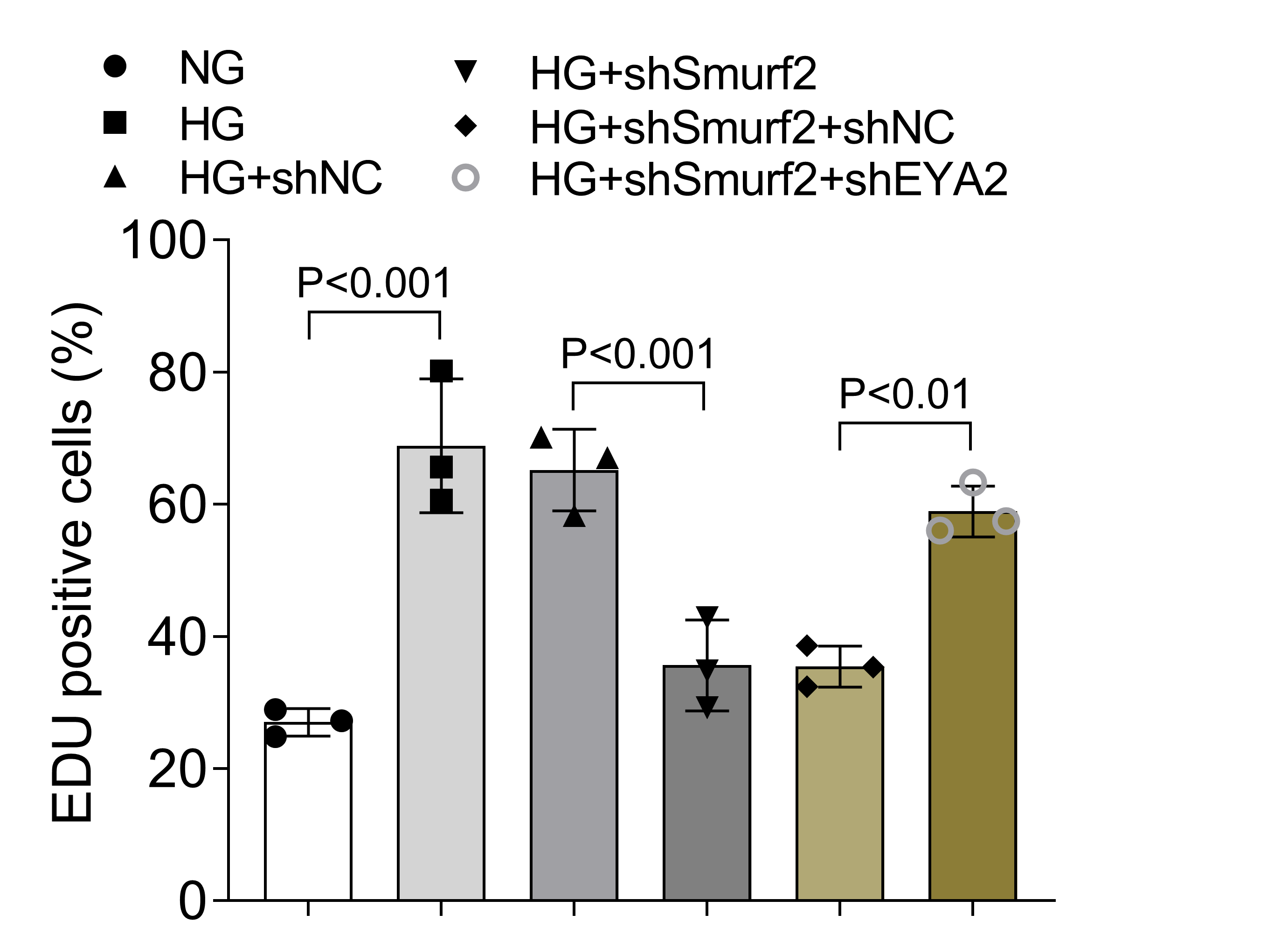

Supplement: figures (1).zip [file IRNF_A_2520904_SM9339.zip › Fig.4/4E.tif]

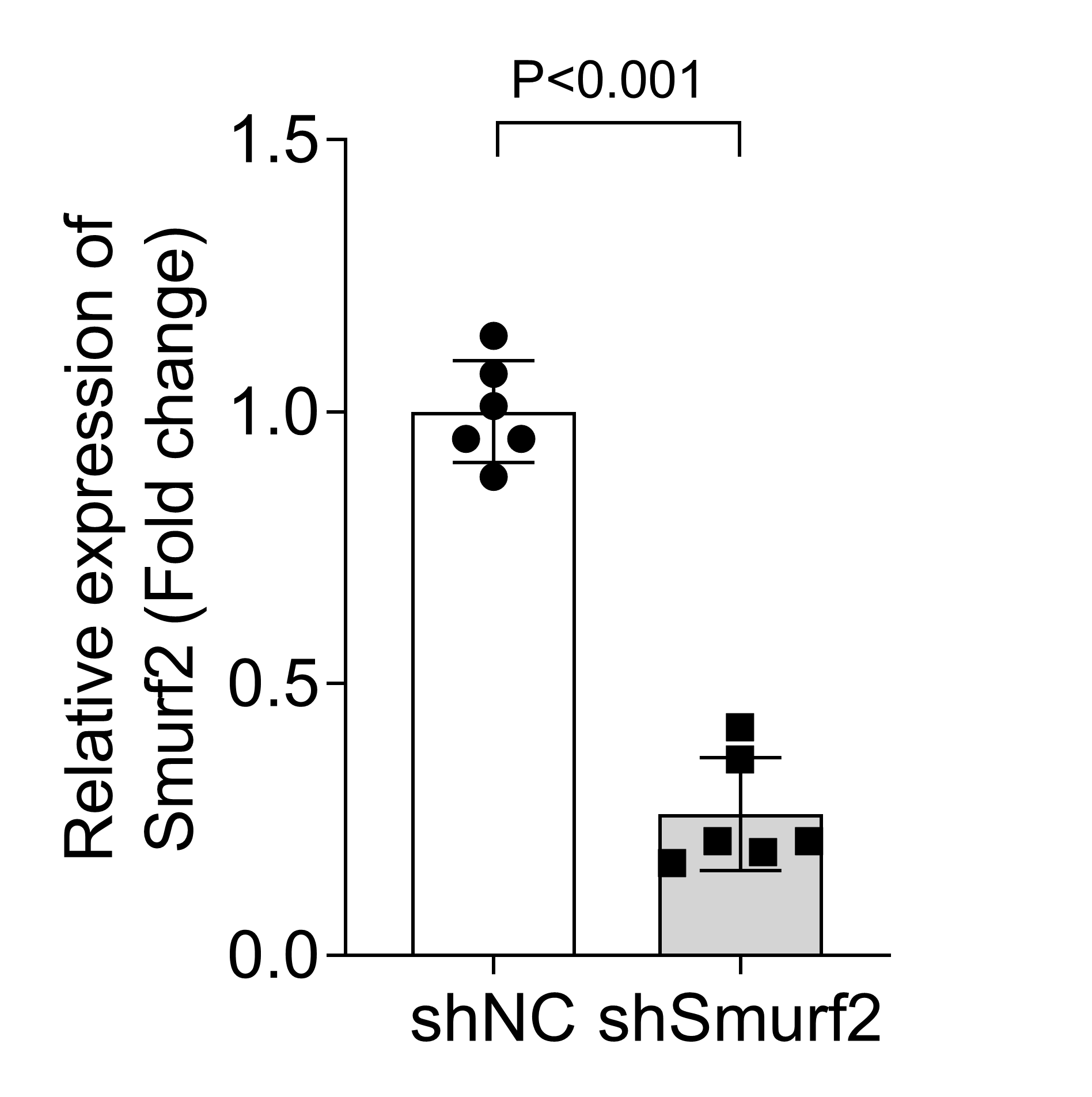

Supplement: figures (1).zip [file IRNF_A_2520904_SM9339.zip › Fig.5/5A.tif]

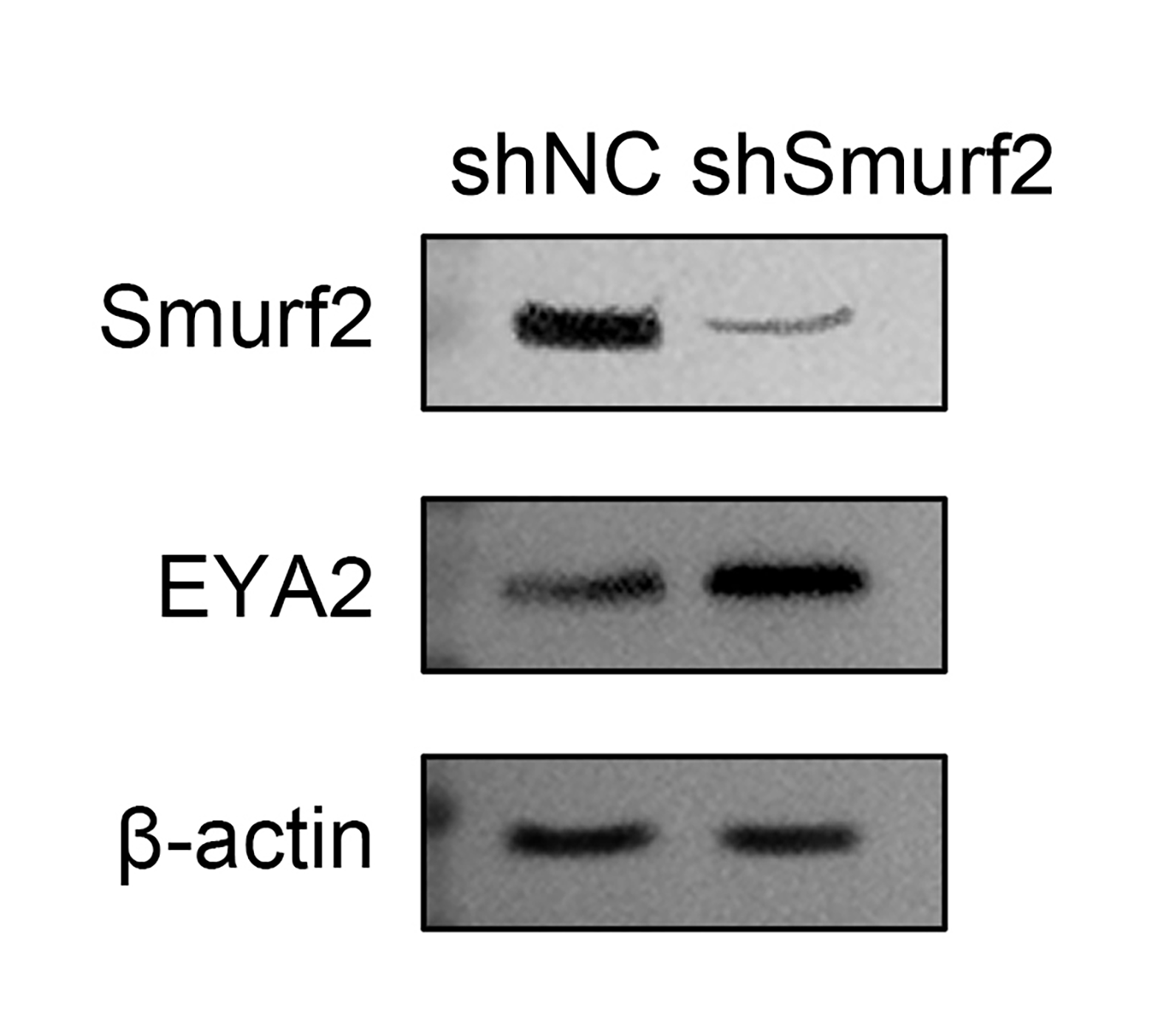

Supplement: figures (1).zip [file IRNF_A_2520904_SM9339.zip › Fig.5/5B.tif]

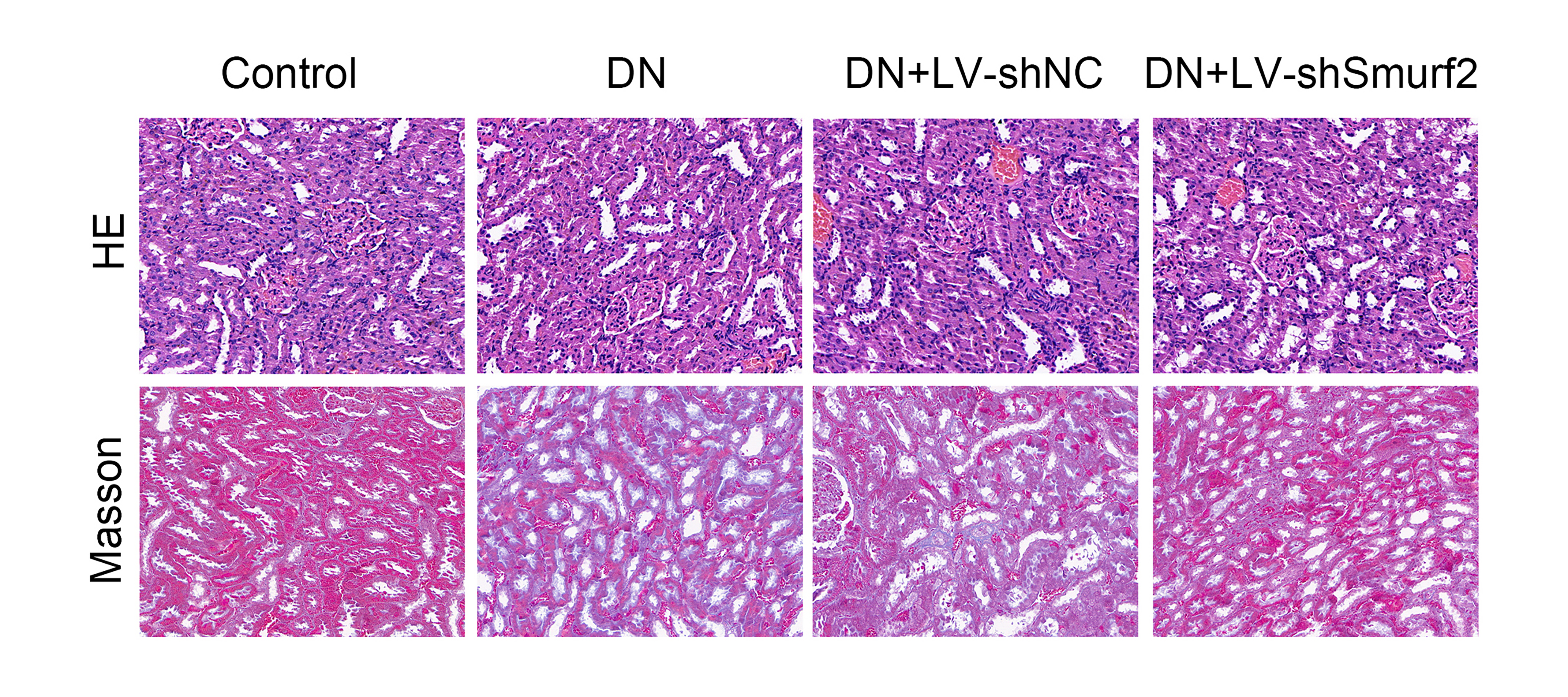

Supplement: figures (1).zip [file IRNF_A_2520904_SM9339.zip › Fig.5/5C.tif]

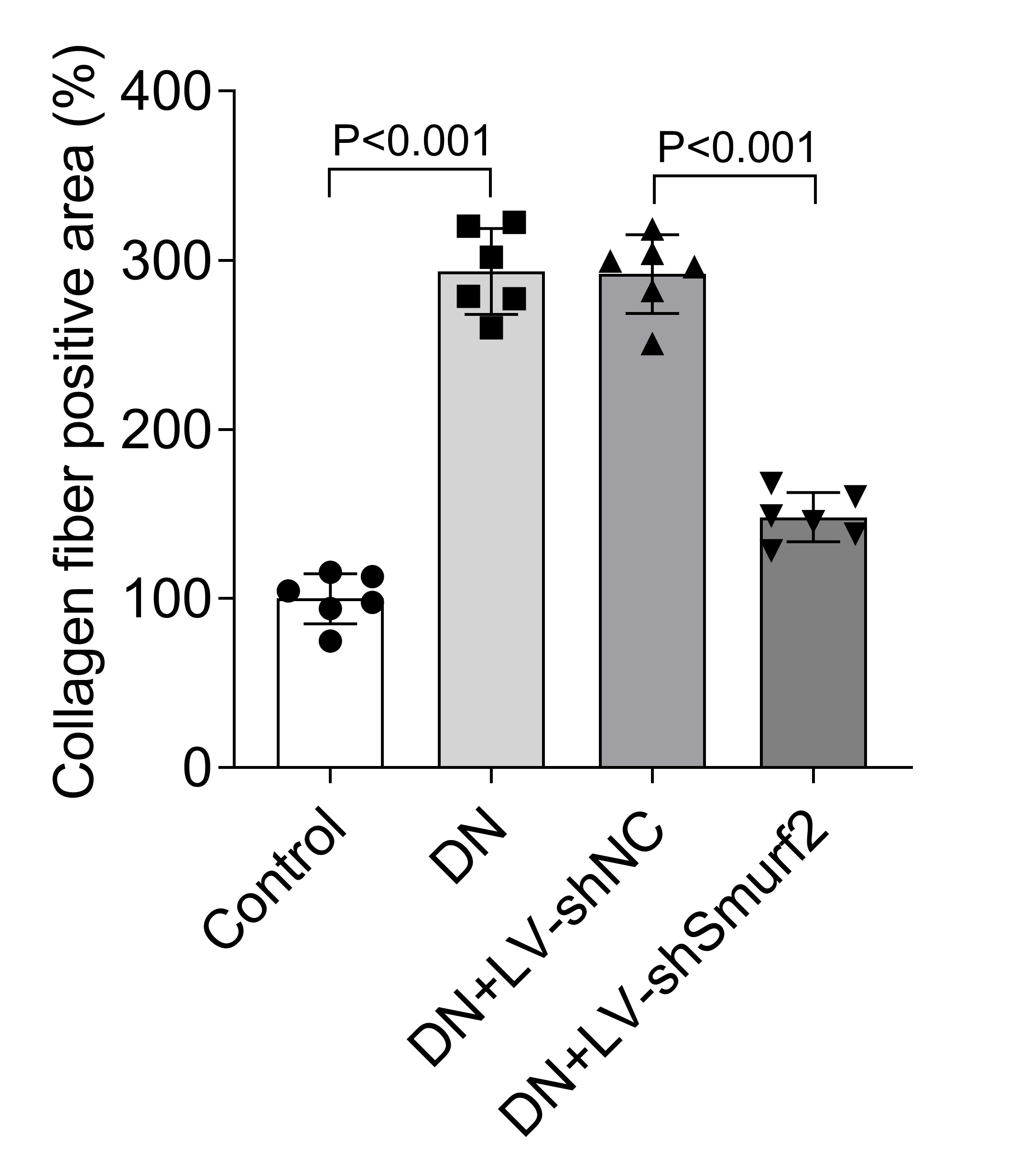

Supplement: figures (1).zip [file IRNF_A_2520904_SM9339.zip › Fig.5/5D.tif]

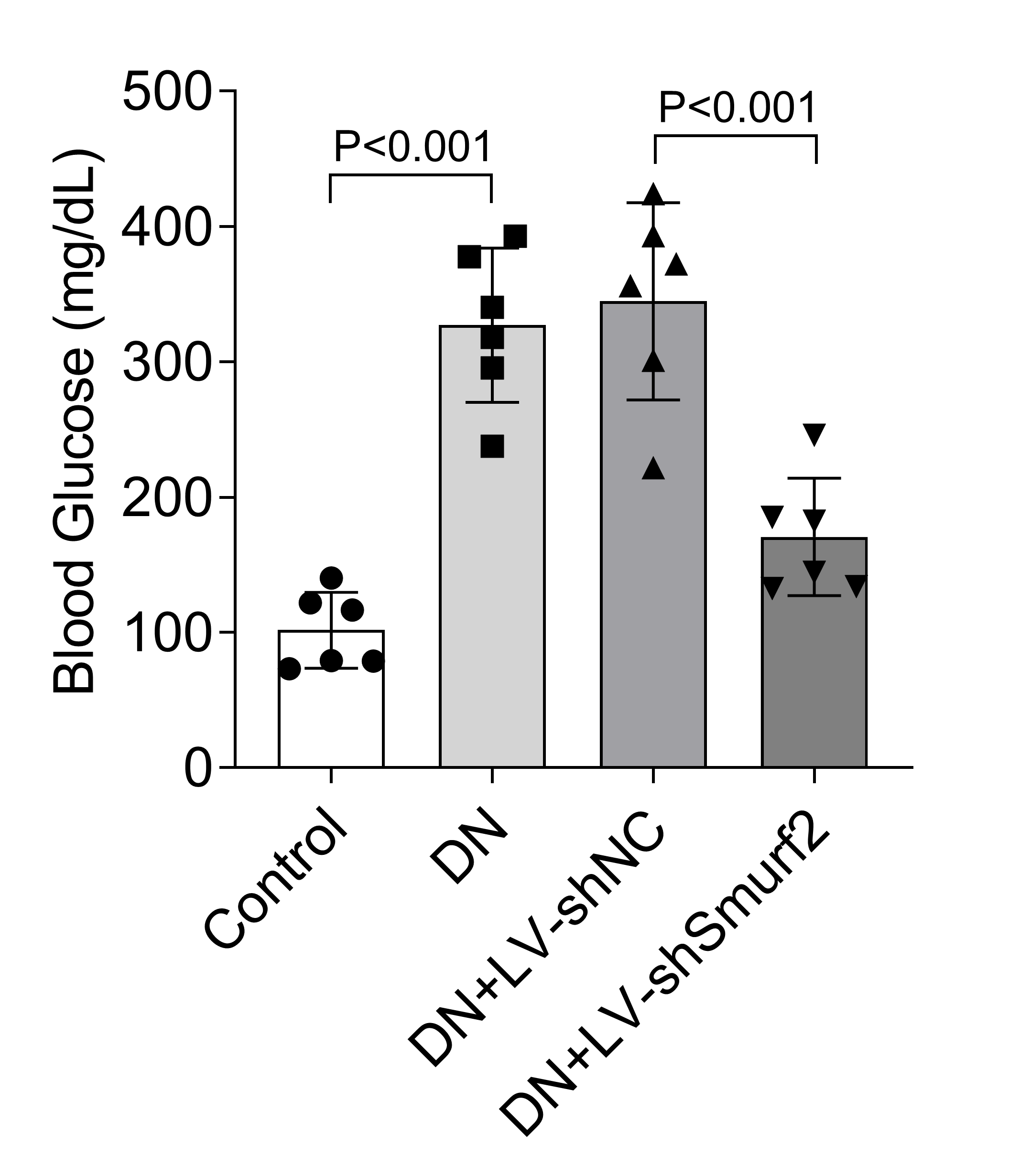

Supplement: figures (1).zip [file IRNF_A_2520904_SM9339.zip › Fig.5/5E.tif]

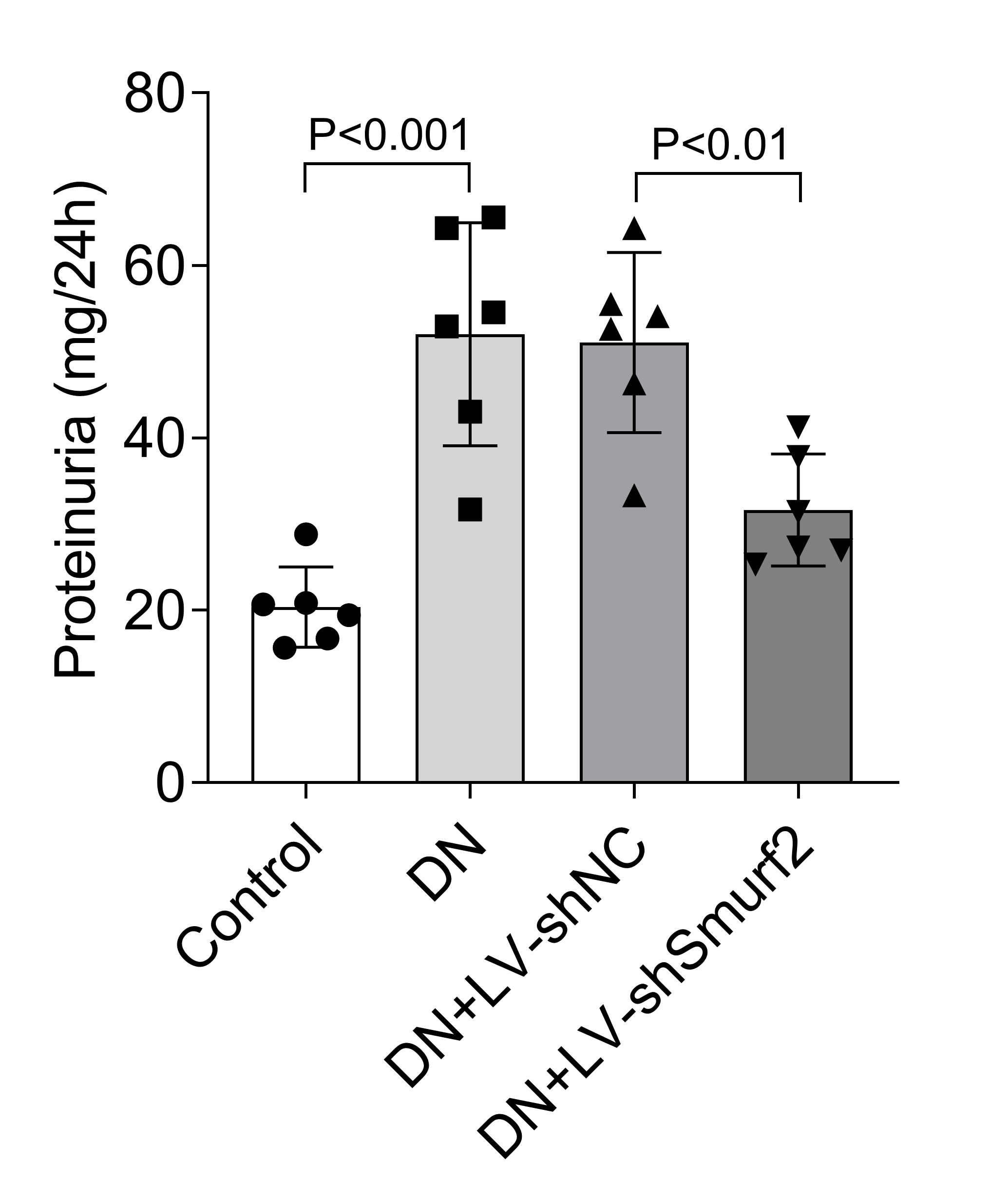

Supplement: figures (1).zip [file IRNF_A_2520904_SM9339.zip › Fig.5/5F.tif]

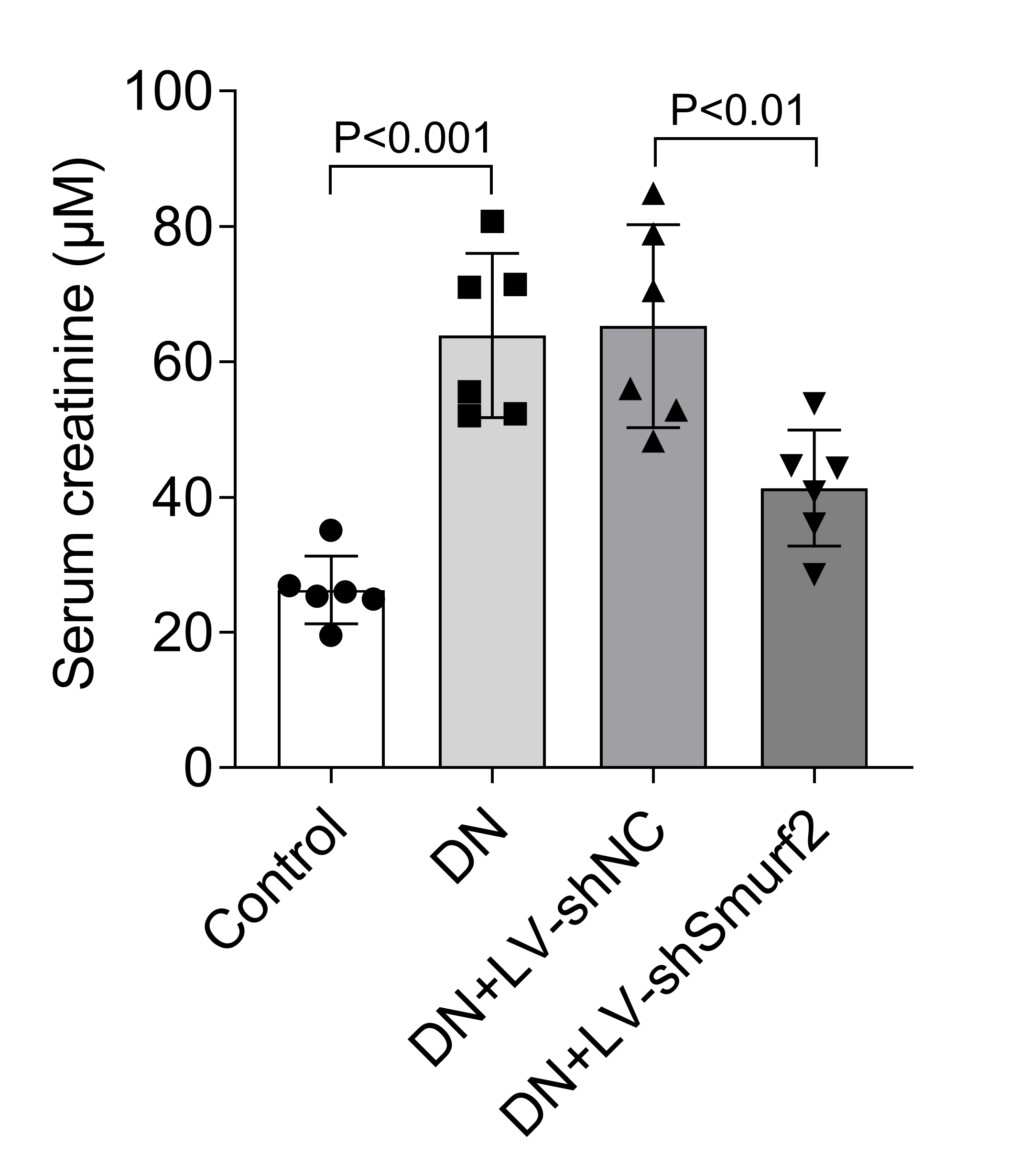

Supplement: figures (1).zip [file IRNF_A_2520904_SM9339.zip › Fig.5/5G.tif]

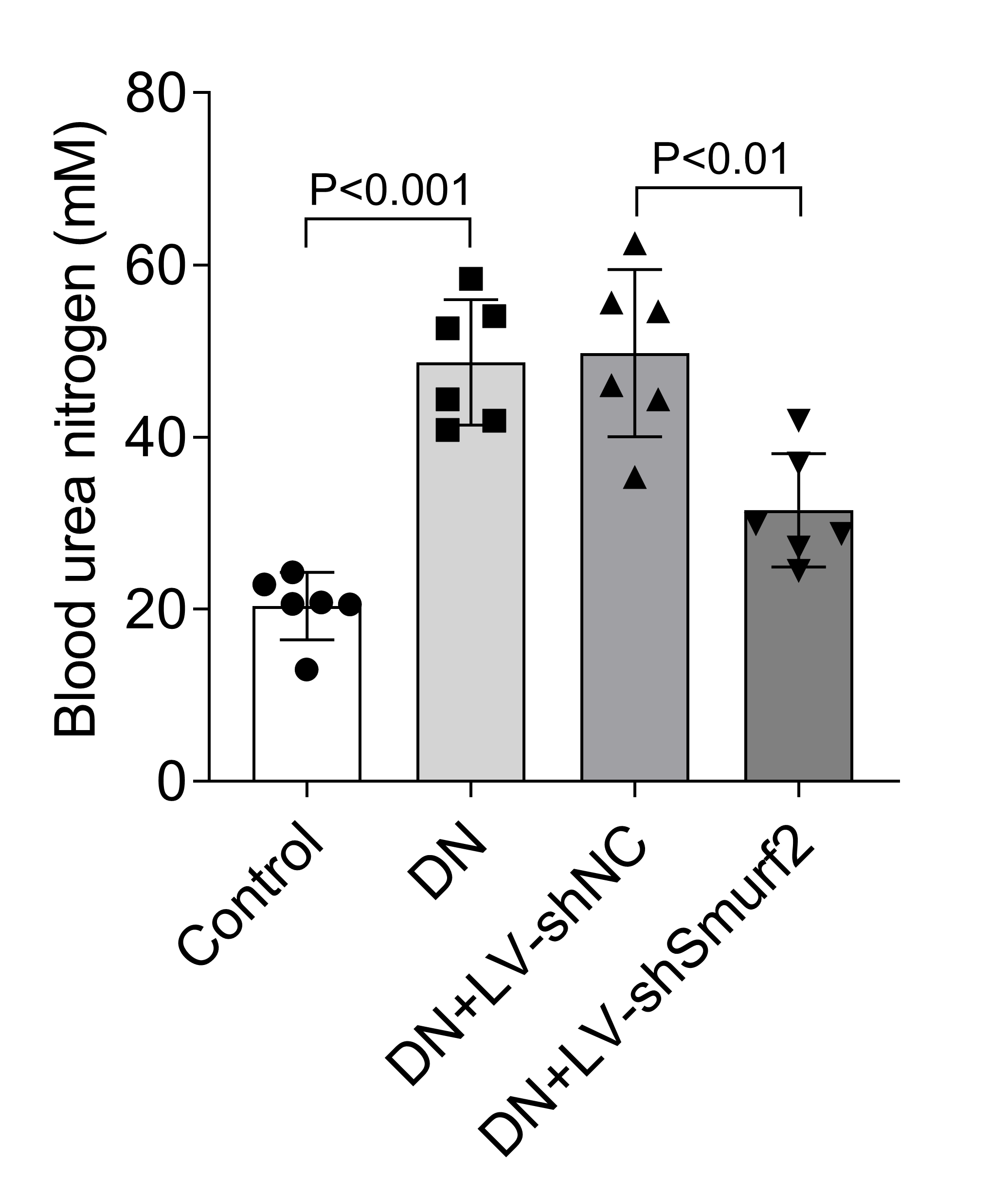

Supplement: figures (1).zip [file IRNF_A_2520904_SM9339.zip › Fig.5/5H.tif]

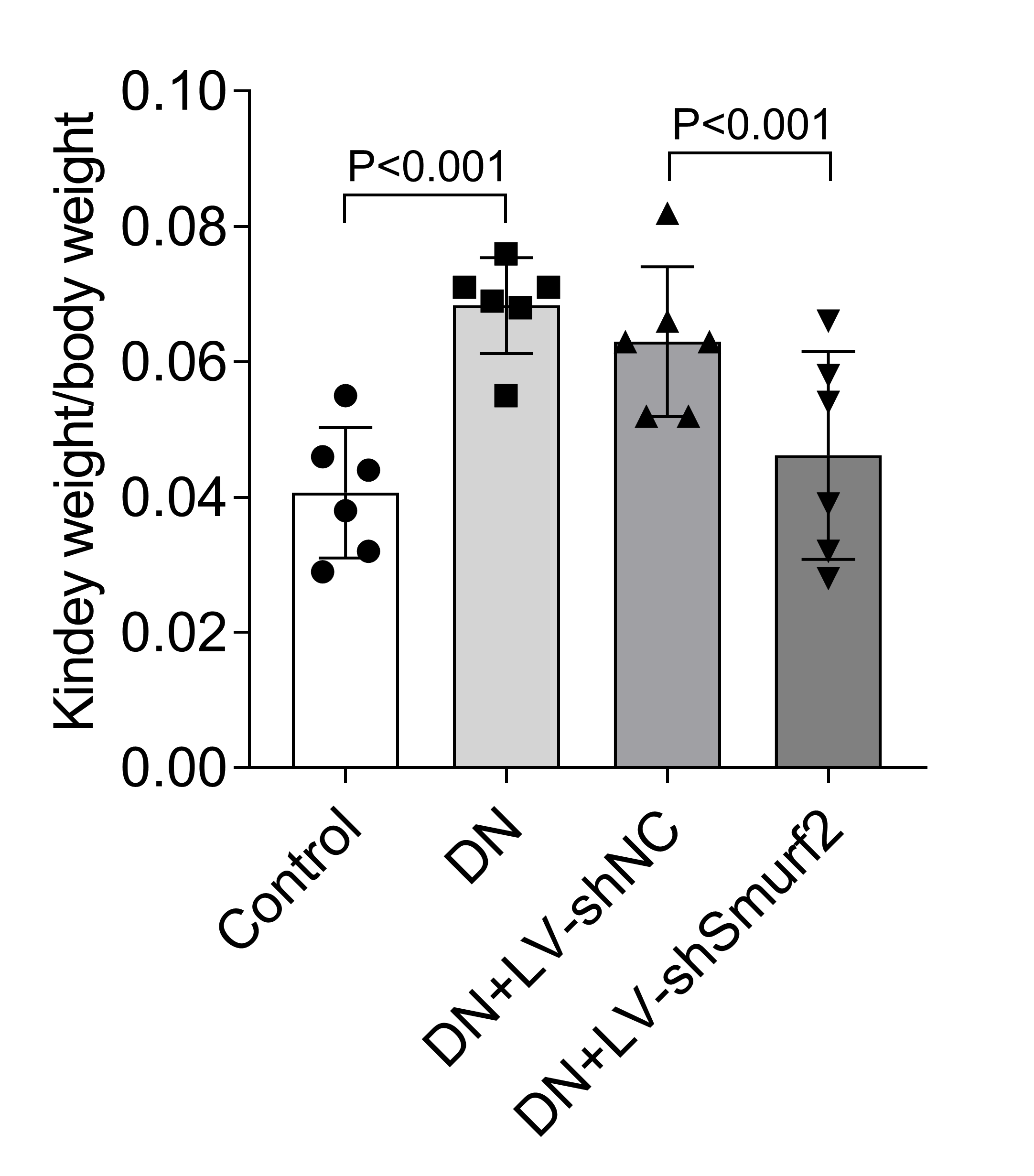

Supplement: figures (1).zip [file IRNF_A_2520904_SM9339.zip › Fig.5/5I.tif]

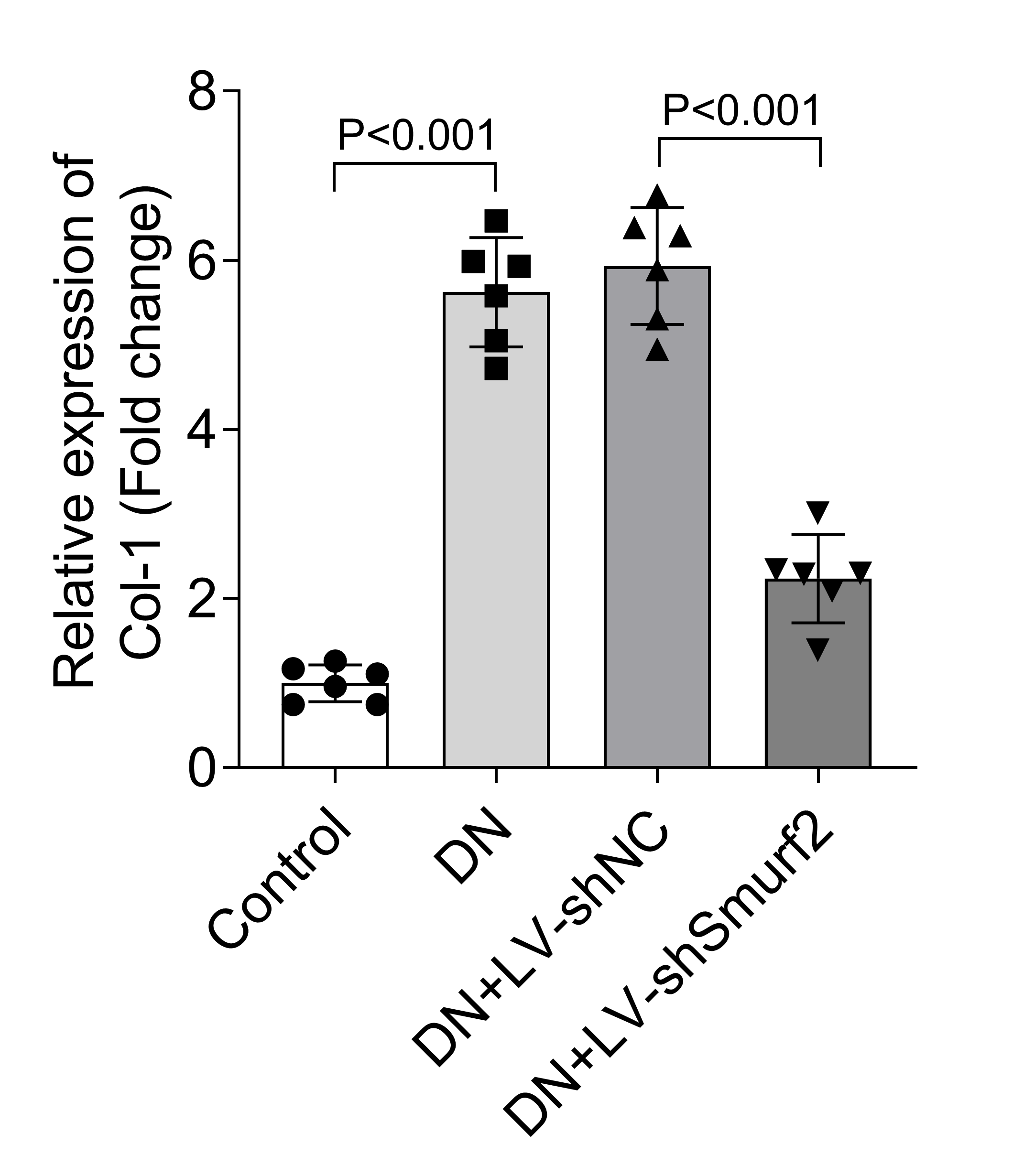

Supplement: figures (1).zip [file IRNF_A_2520904_SM9339.zip › Fig.5/5J.tif]

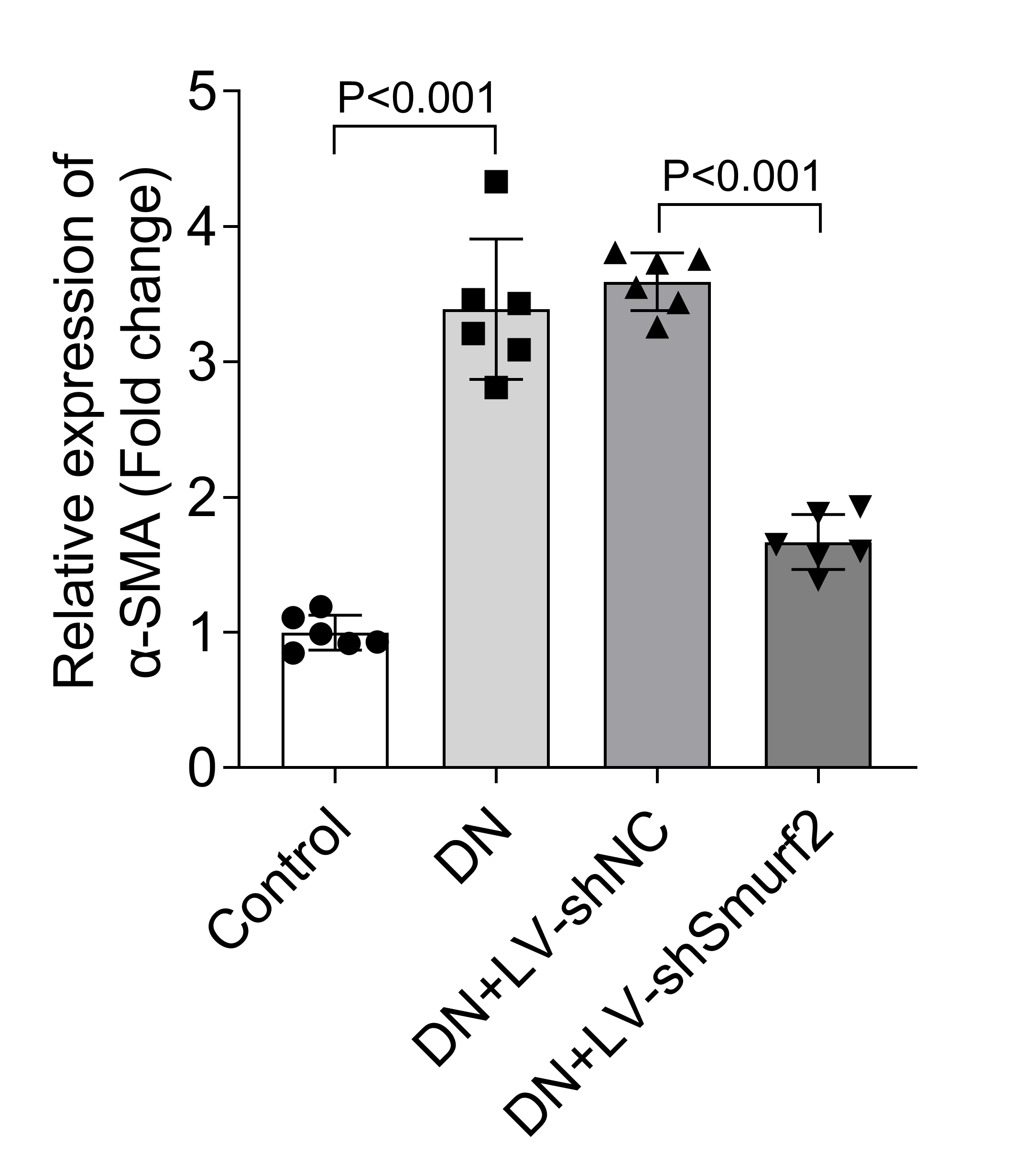

Supplement: figures (1).zip [file IRNF_A_2520904_SM9339.zip › Fig.5/5K.tif]

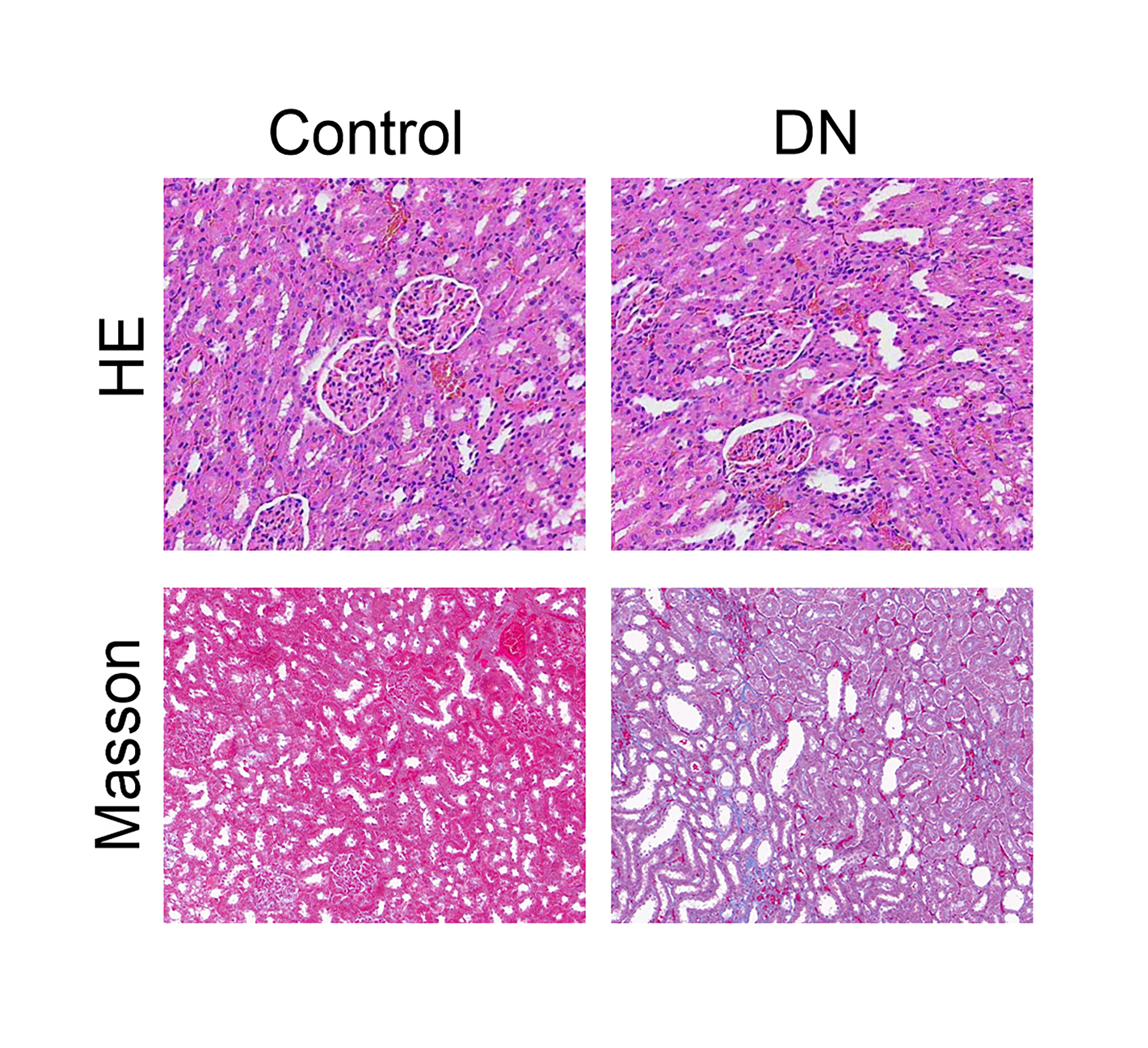

Supplement: figures (1).zip [file IRNF_A_2520904_SM9339.zip › Fig.S1/S1A.tif]

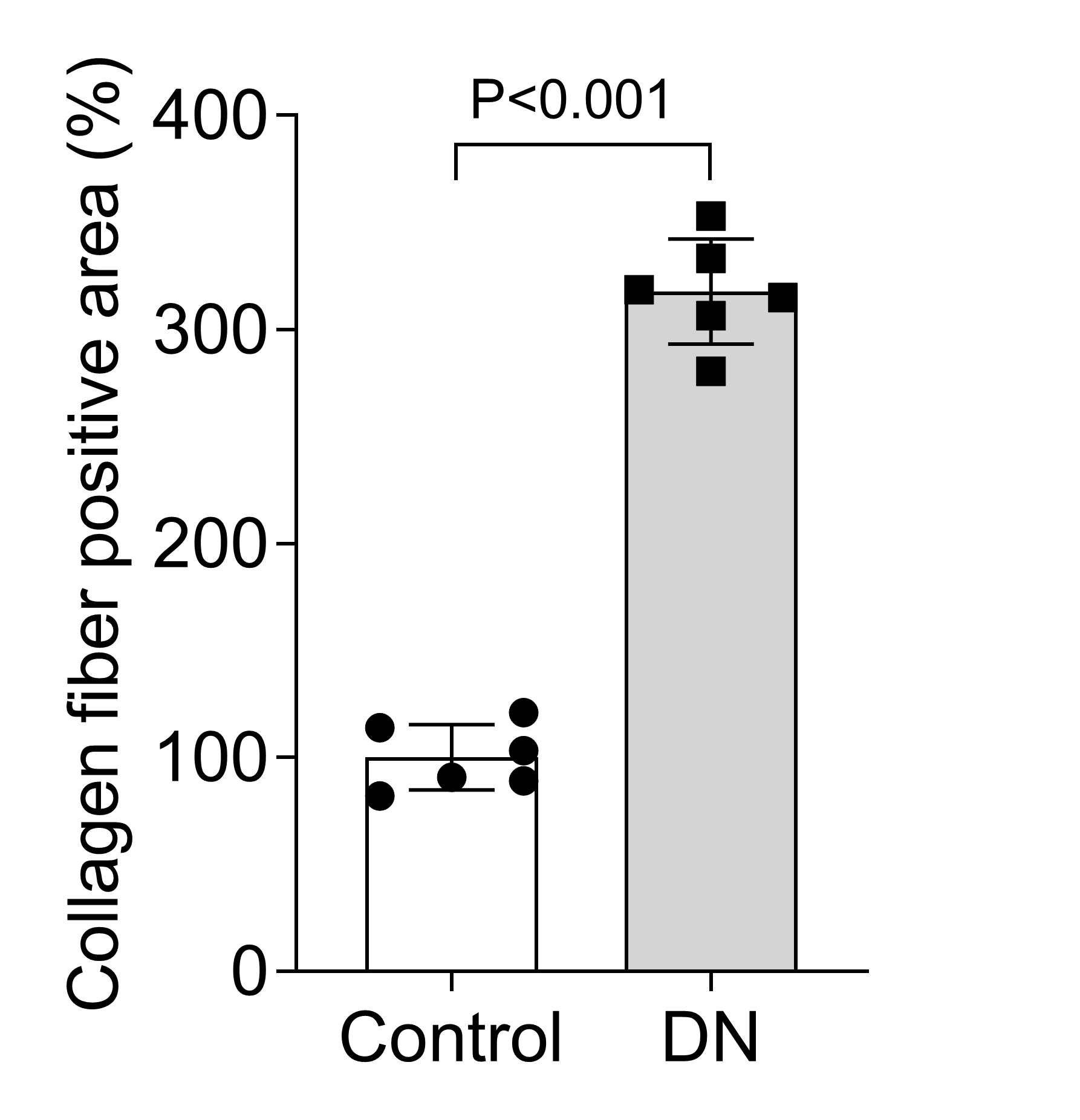

Supplement: figures (1).zip [file IRNF_A_2520904_SM9339.zip › Fig.S1/S1B.tif]

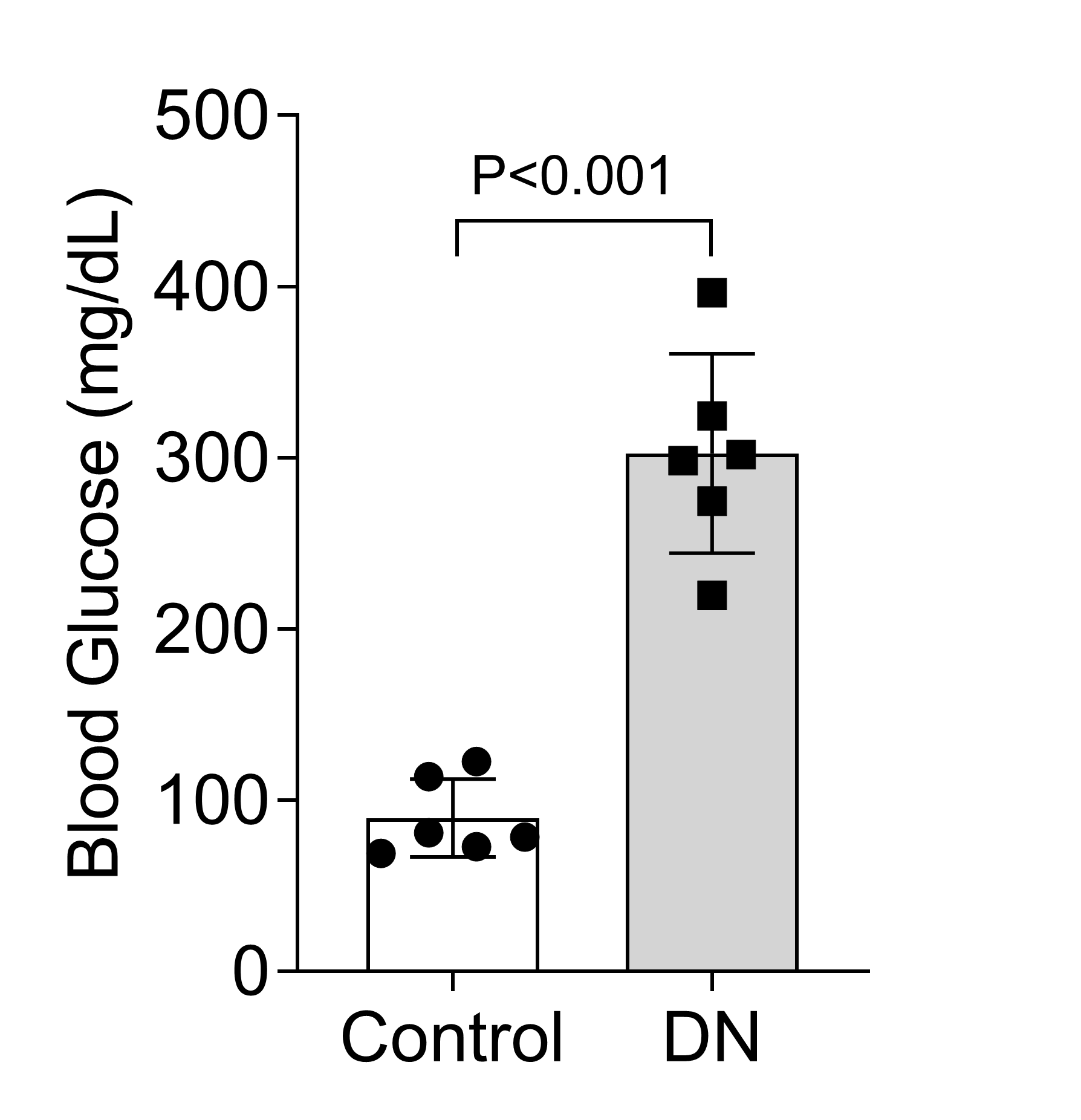

Supplement: figures (1).zip [file IRNF_A_2520904_SM9339.zip › Fig.S1/S1C.tif]

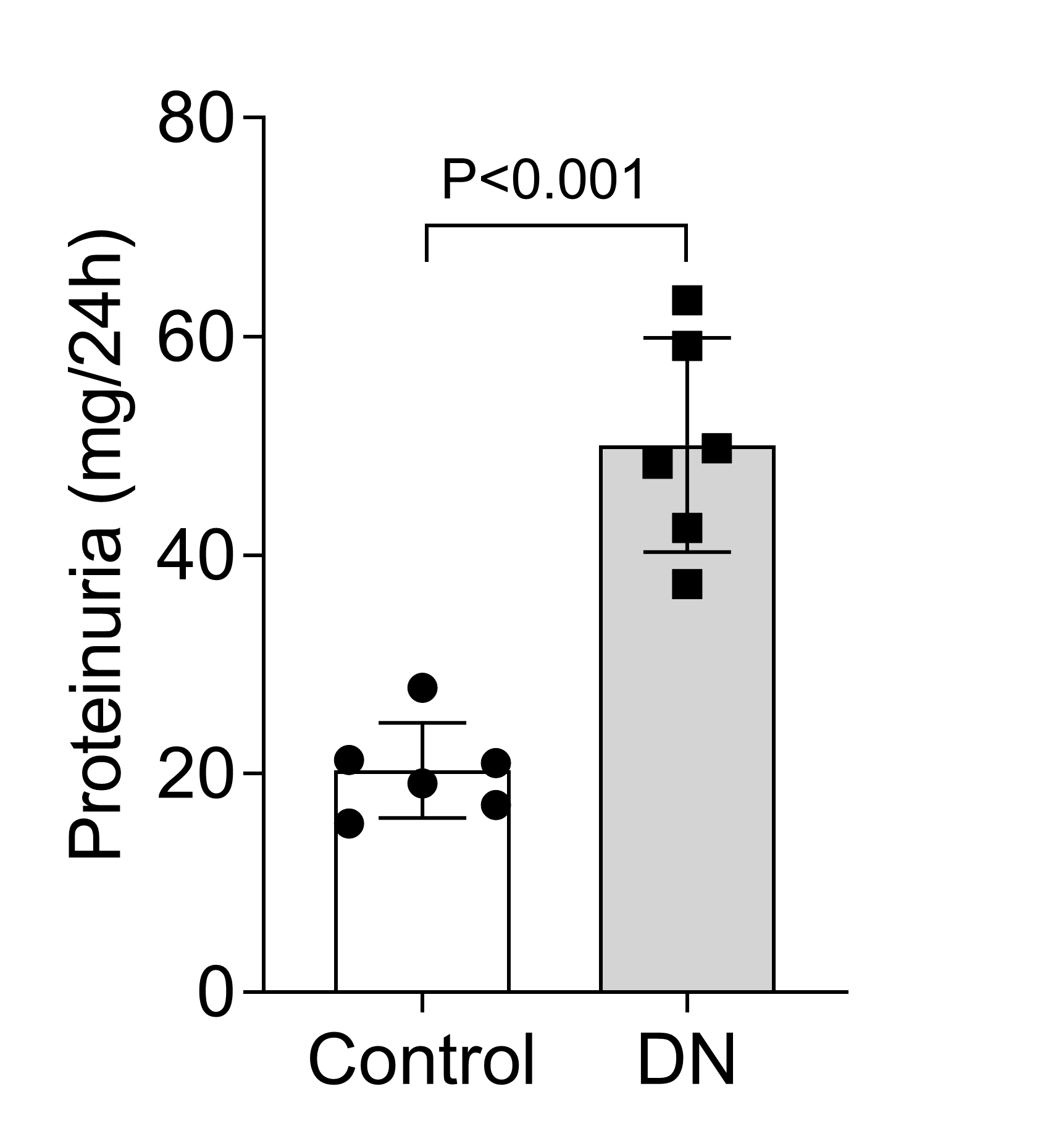

Supplement: figures (1).zip [file IRNF_A_2520904_SM9339.zip › Fig.S1/S1D.tif]

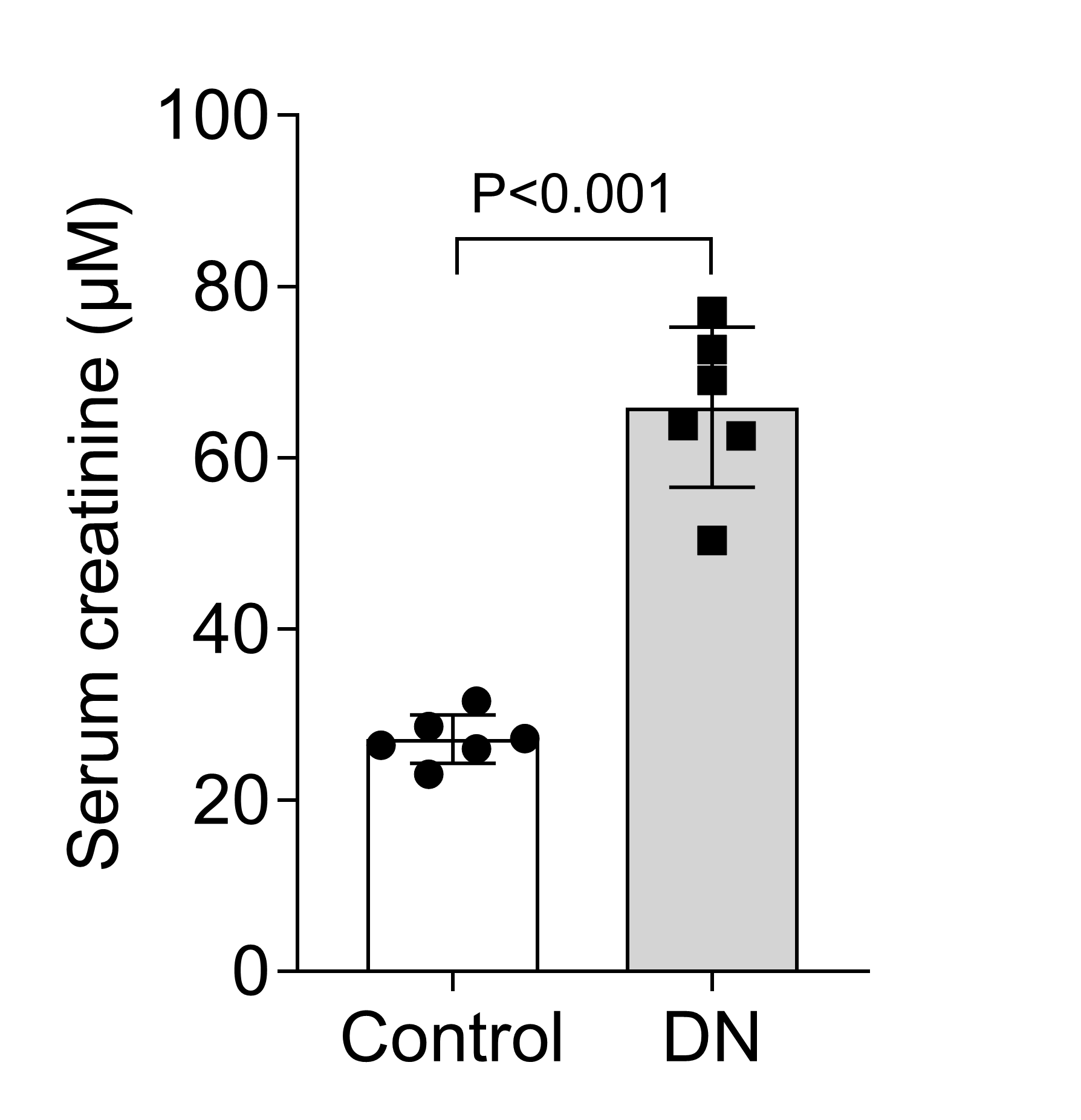

Supplement: figures (1).zip [file IRNF_A_2520904_SM9339.zip › Fig.S1/S1E.tif]

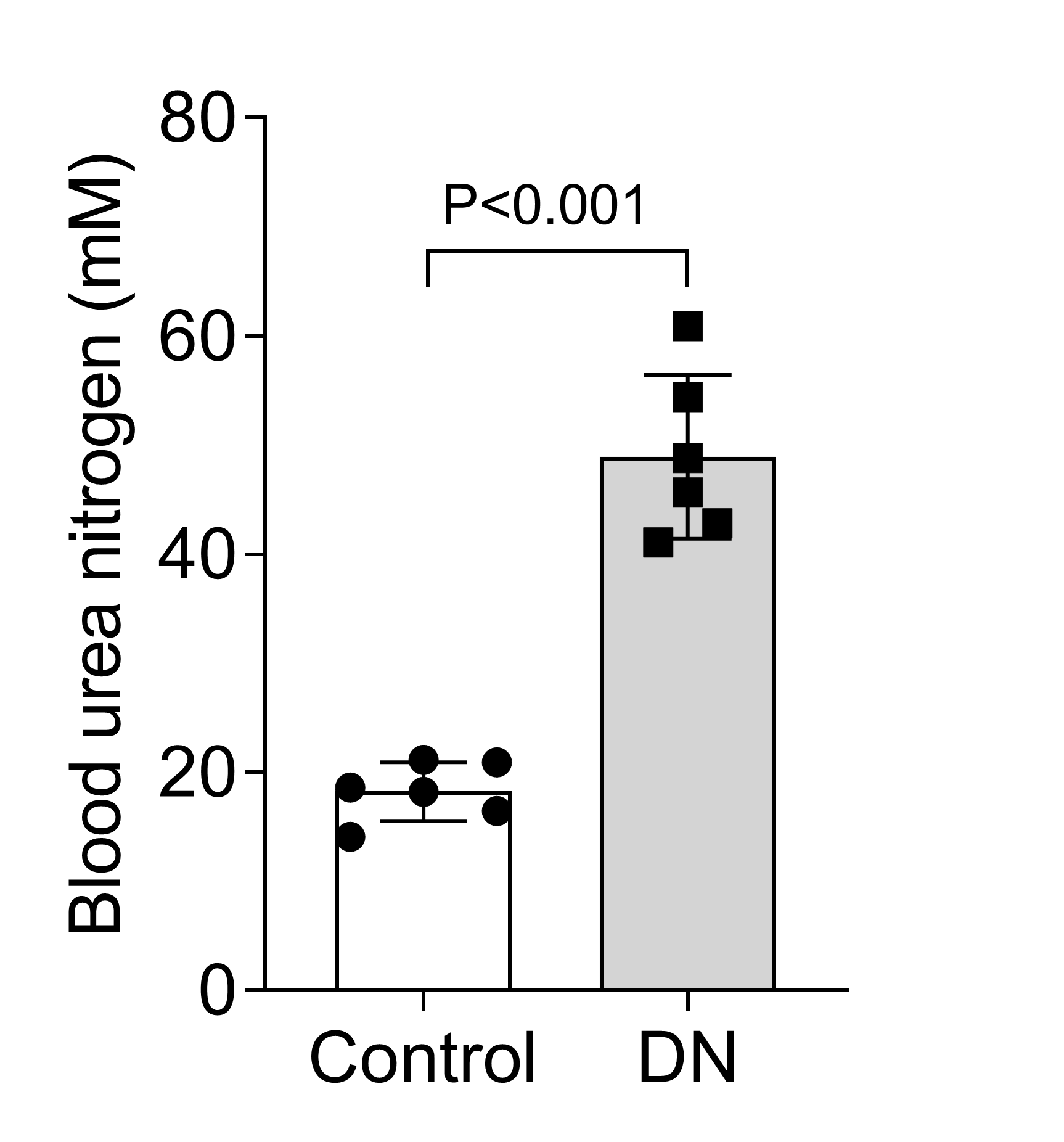

Supplement: figures (1).zip [file IRNF_A_2520904_SM9339.zip › Fig.S1/S1F.tif]

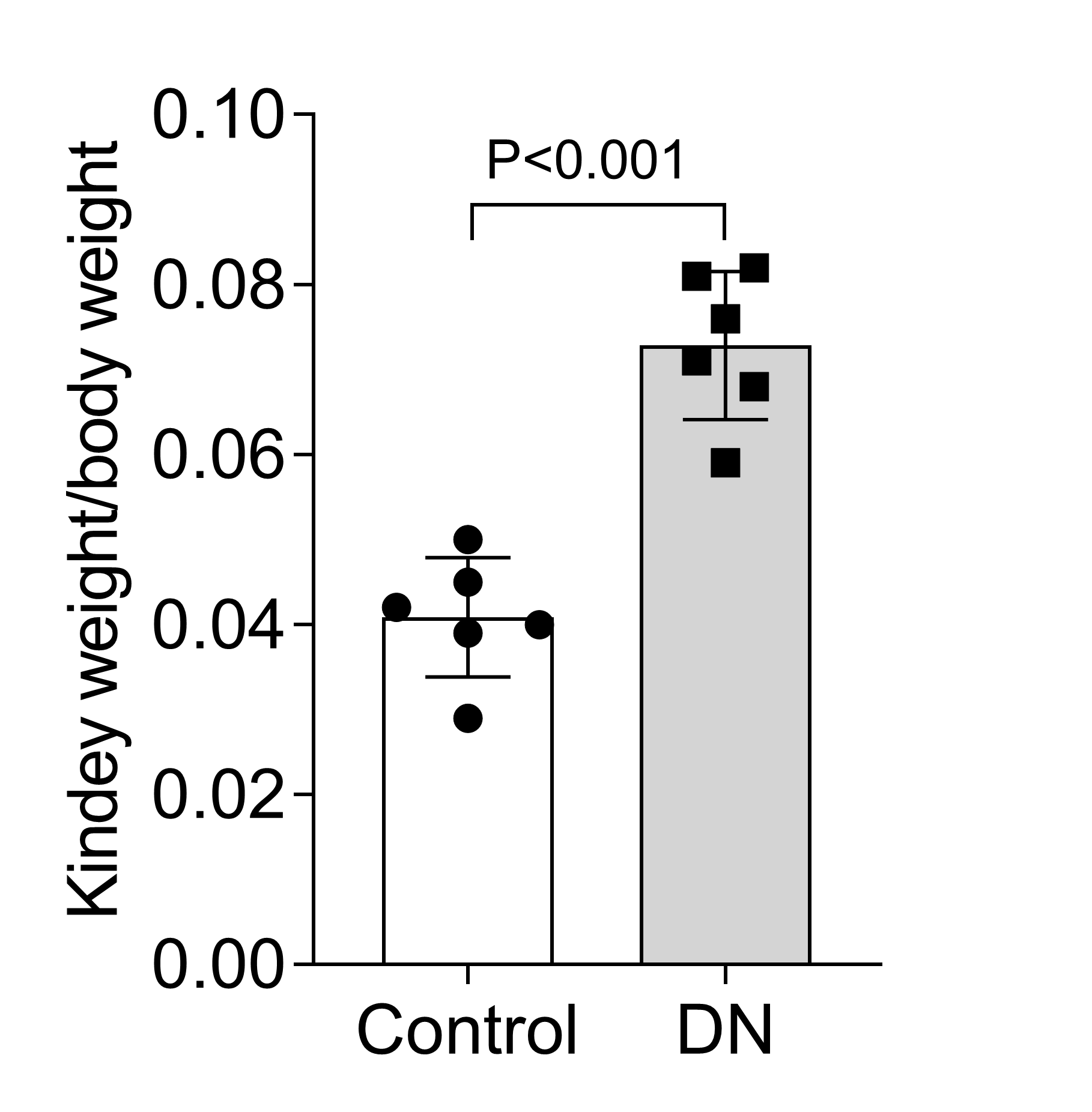

Supplement: figures (1).zip [file IRNF_A_2520904_SM9339.zip › Fig.S1/S1G.tif]

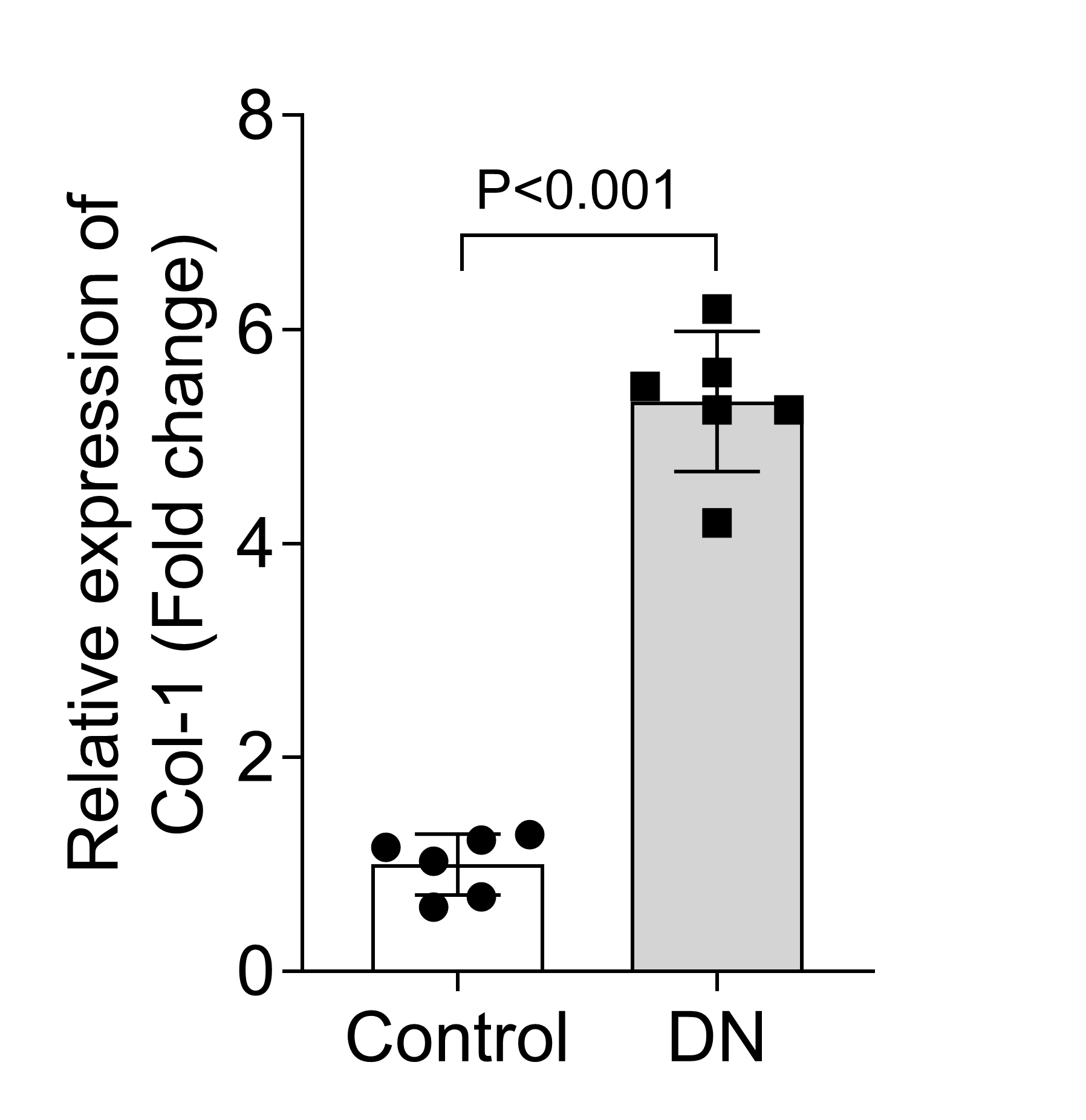

Supplement: figures (1).zip [file IRNF_A_2520904_SM9339.zip › Fig.S1/S1H.tif]

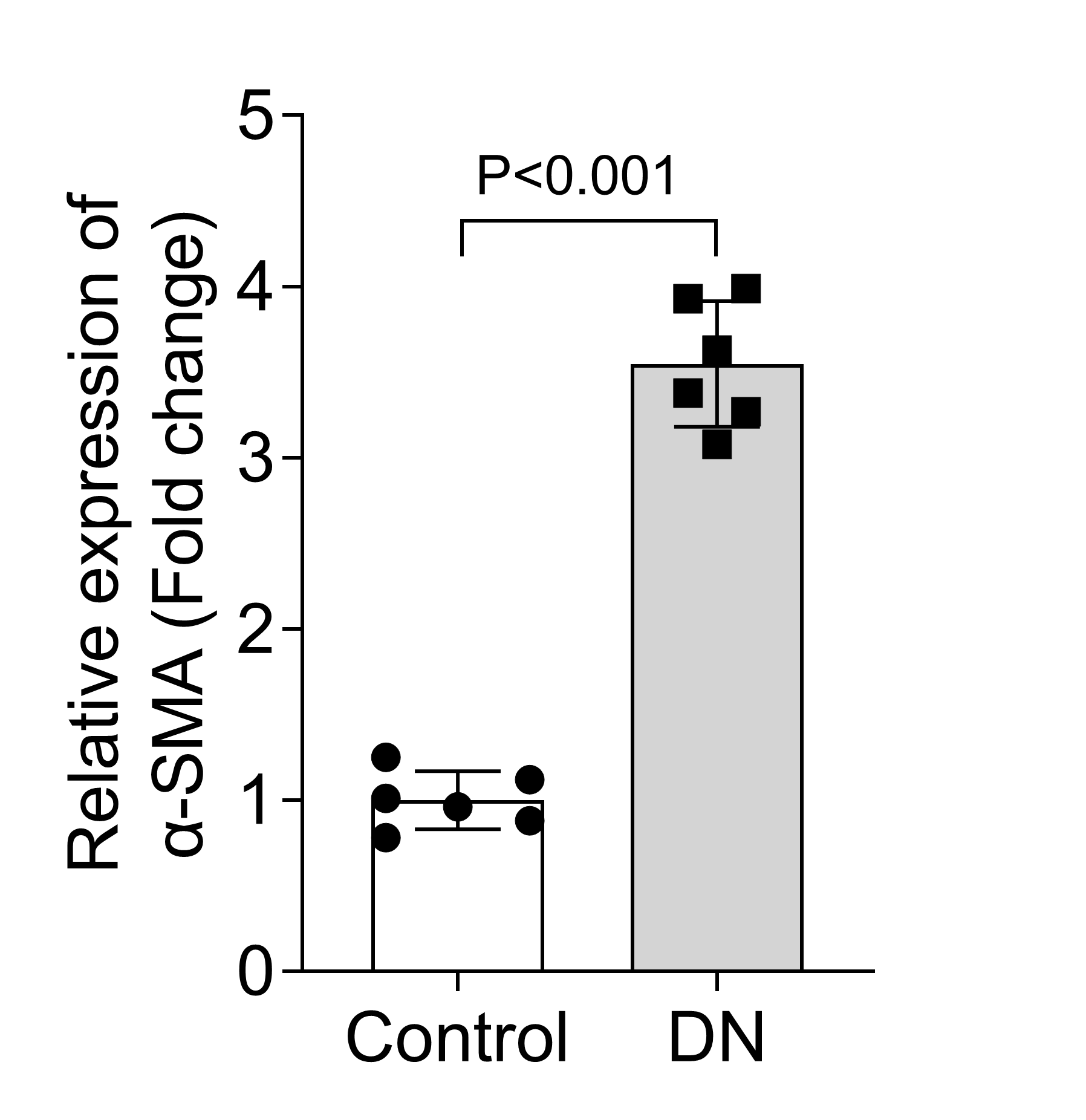

Supplement: figures (1).zip [file IRNF_A_2520904_SM9339.zip › Fig.S1/S1I.tif]

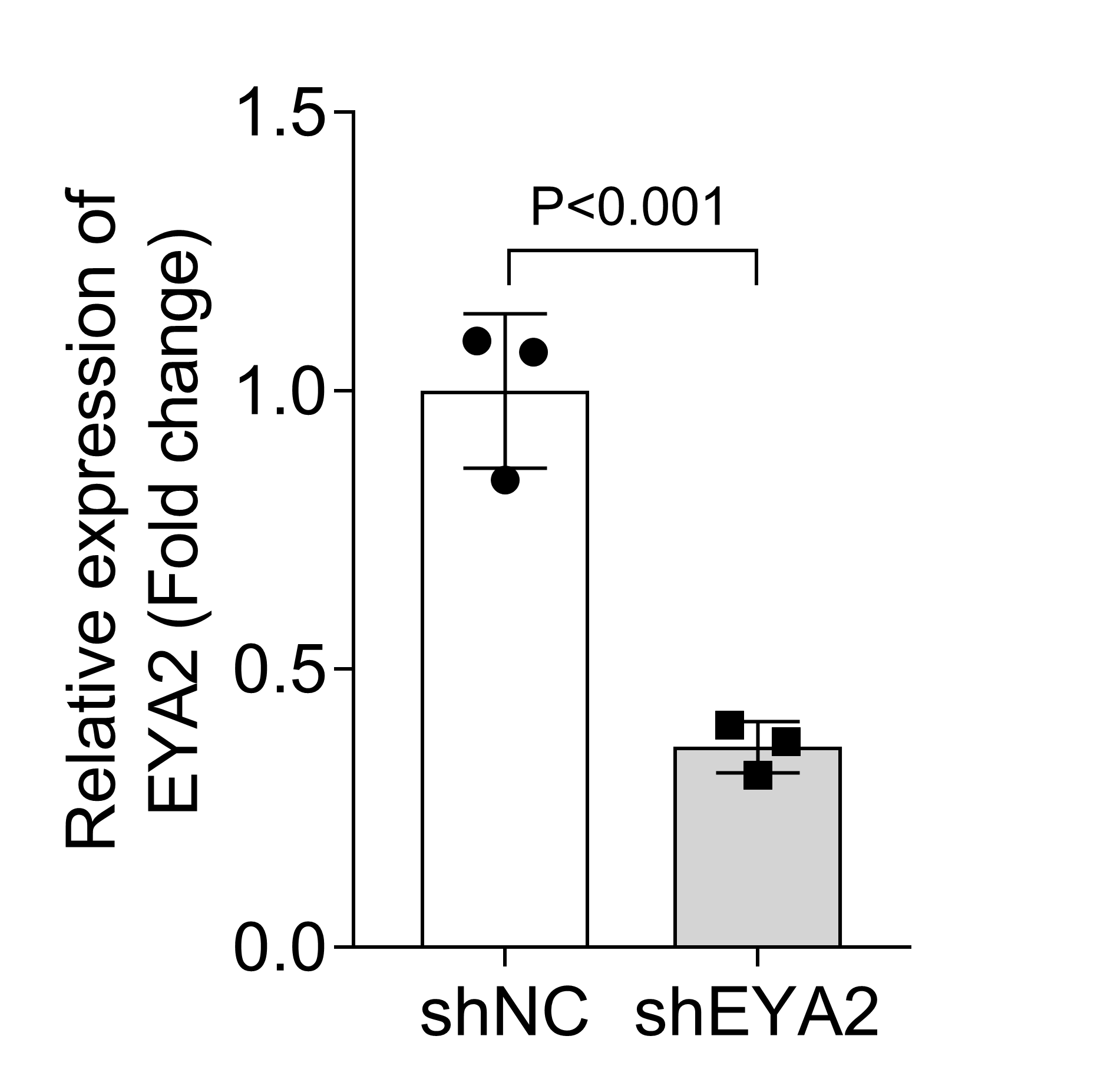

Supplement: figures (1).zip [file IRNF_A_2520904_SM9339.zip › Fig.S2/S2A.tif]

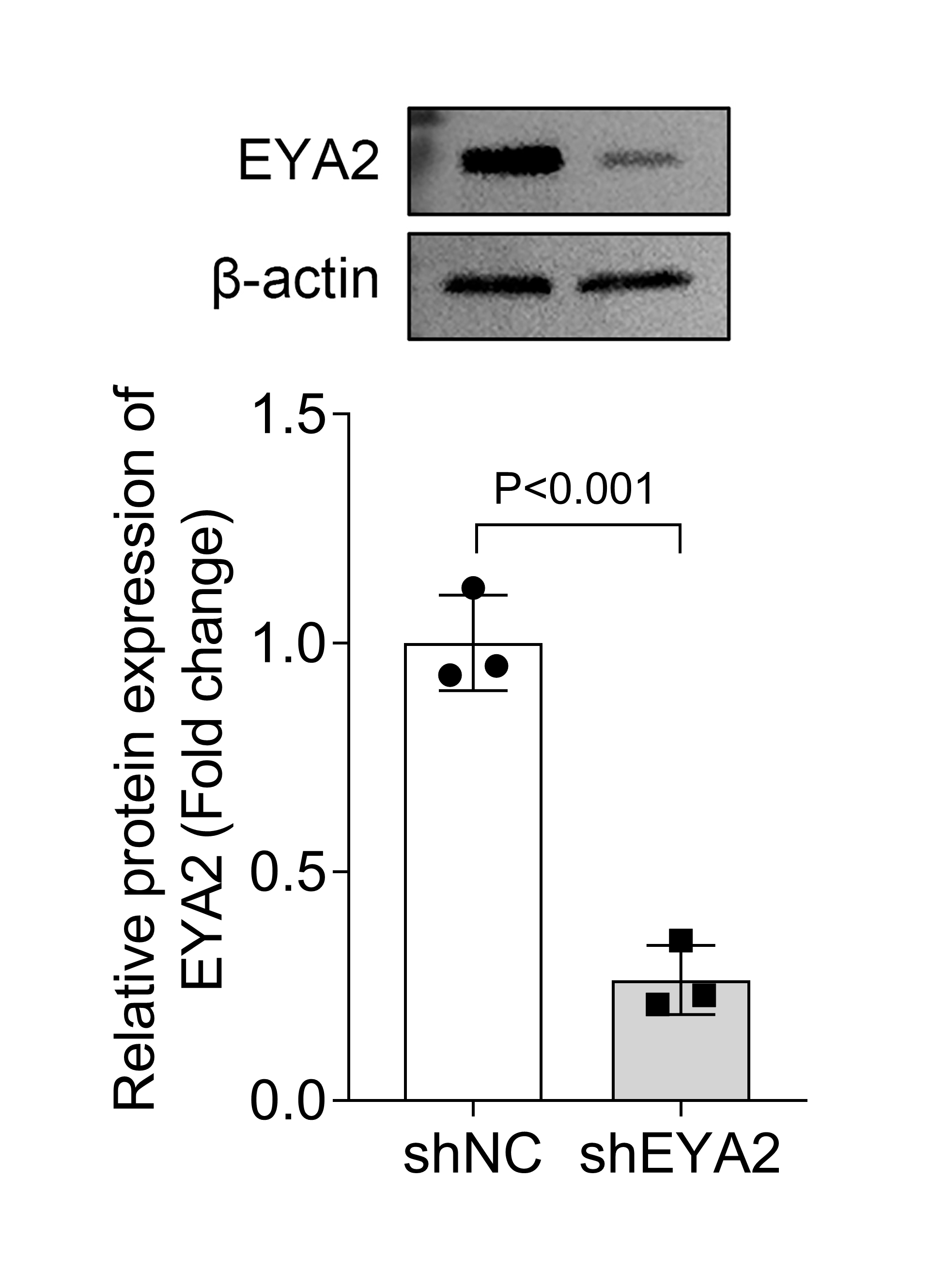

Supplement: figures (1).zip [file IRNF_A_2520904_SM9339.zip › Fig.S2/S2B.tif]

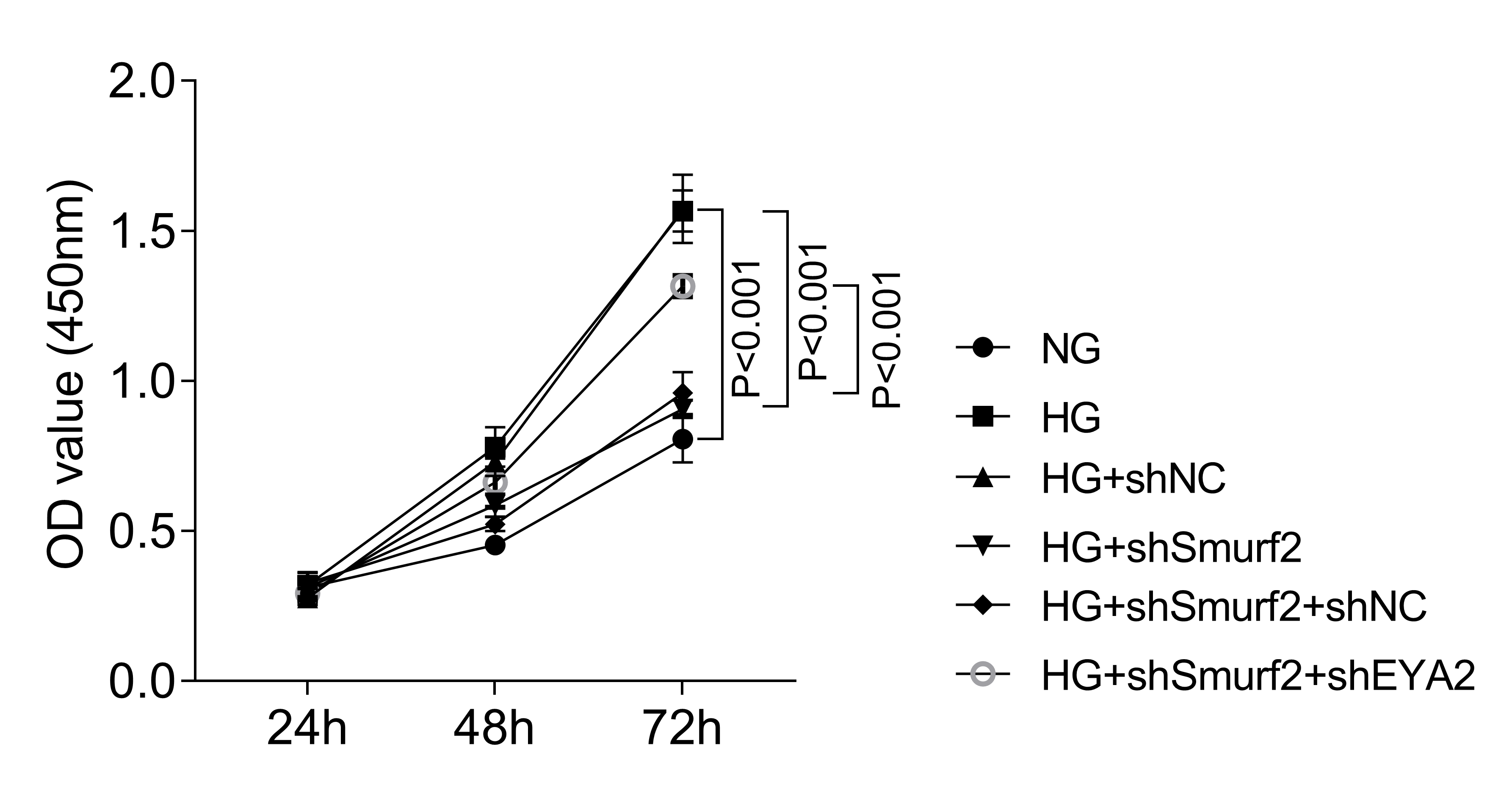

Supplement: figures (1).zip [file IRNF_A_2520904_SM9339.zip › Fig.S2/S2C.tif]

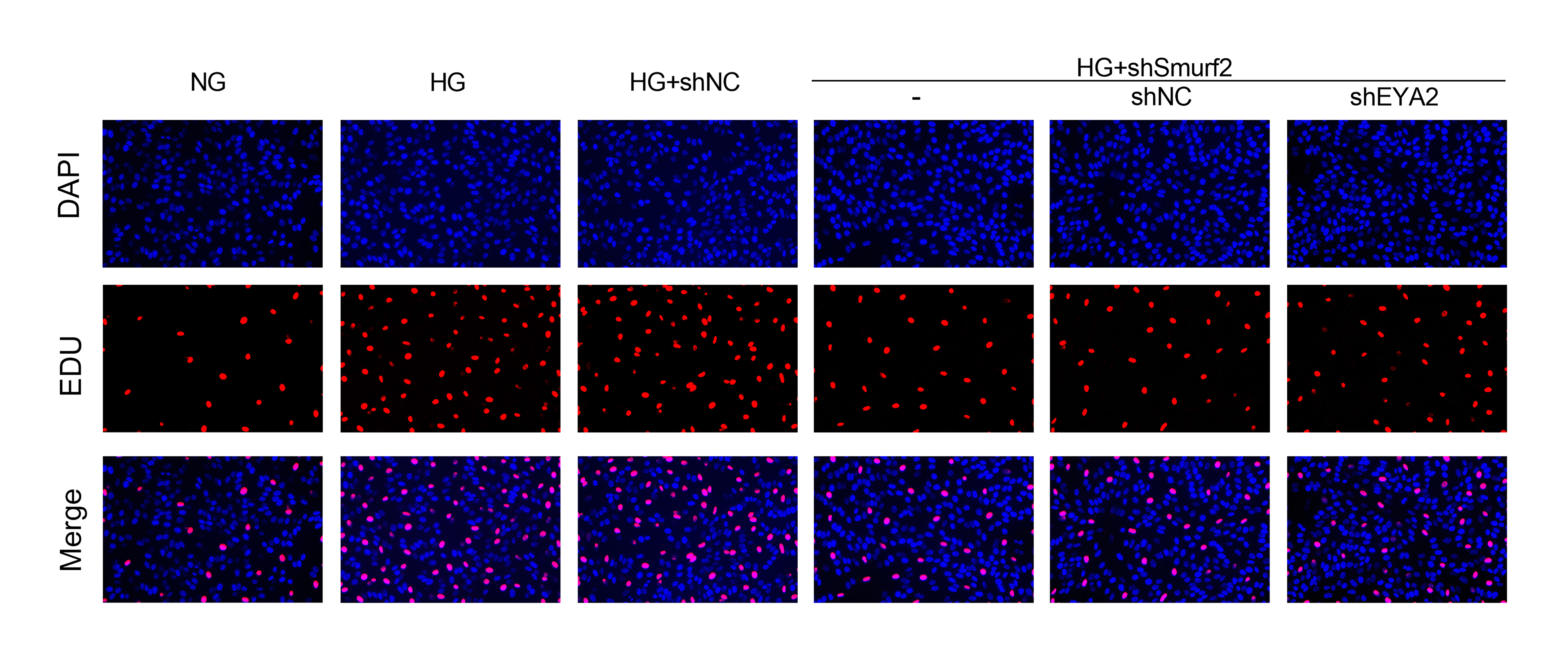

Supplement: figures (1).zip [file IRNF_A_2520904_SM9339.zip › Fig.S2/S2D.tif]

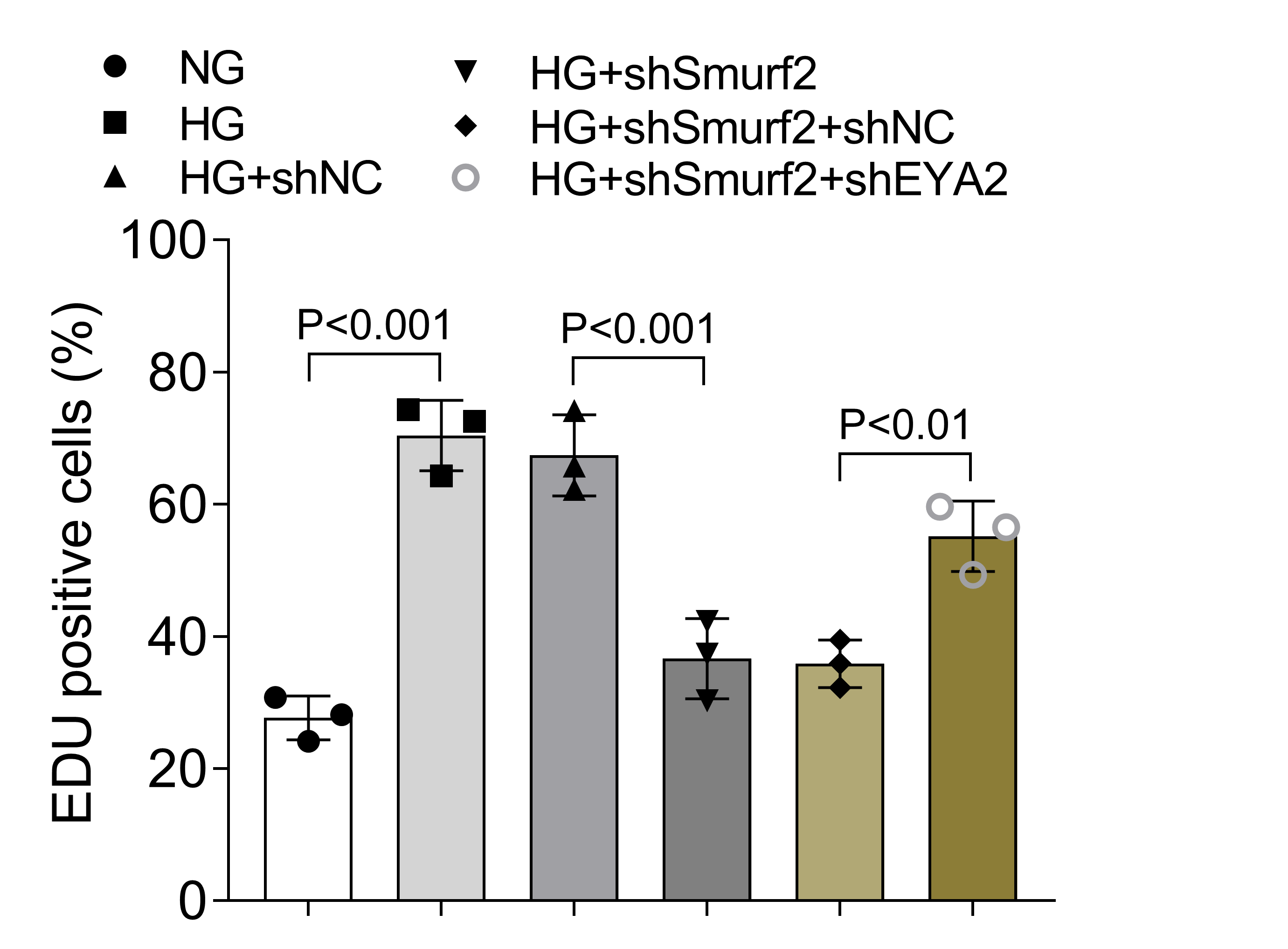

Supplement: figures (1).zip [file IRNF_A_2520904_SM9339.zip › Fig.S2/S2E.tif]

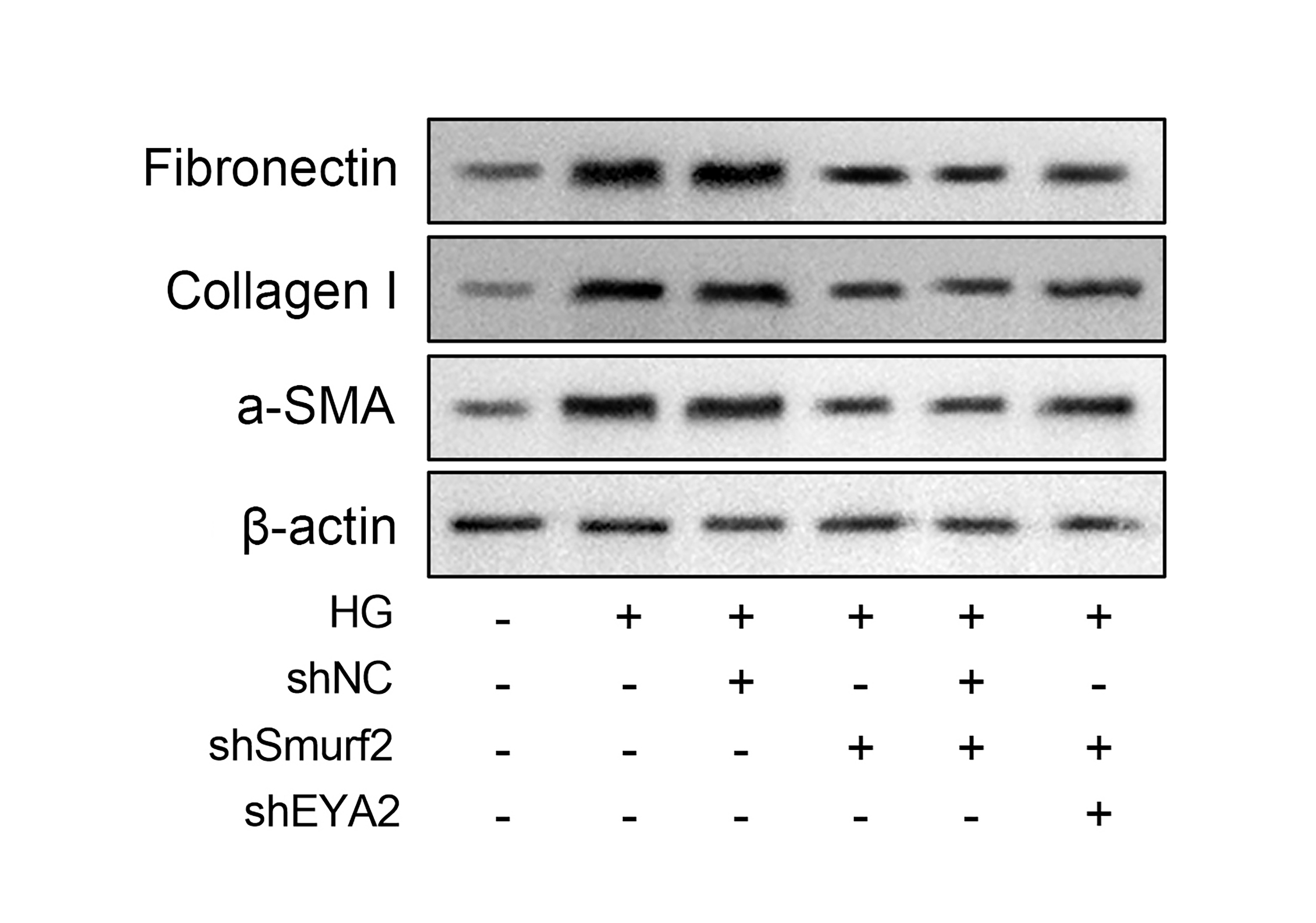

Supplement: figures (1).zip [file IRNF_A_2520904_SM9339.zip › Fig.S2/S2F.tif]
